# Acyl Donor Intermediates in N‐Heterocyclic Carbene Catalysis: Acyl Azolium or Azolium Enolate?

**DOI:** 10.1002/anie.202010348

**Published:** 2021-01-18

**Authors:** Animesh Biswas, Jörg‐M. Neudörfl, Nils E. Schlörer, Albrecht Berkessel

**Affiliations:** ^1^ Department of Chemistry (Organic Chemistry) University of Cologne Greinstraße 4 50939 Cologne Germany

**Keywords:** esters, N-heterocyclic carbenes, reaction mechanisms, structure elucidation, X-ray diffraction

## Abstract

Azolium enolates and acyl azolium cations have been proposed as intermediates in numerous N‐heterocyclic carbene (NHC) catalyzed transformations. Acetyl azolium enolates were generated from the reaction of 2‐propenyl acetate with both saturated (SIPr) and aromatic (IPr) NHCs, isolated, and characterized (NMR, XRD). Protonation with triflic acid gave the corresponding acetyl azolium triflates which were isolated and characterized (NMR, XRD). Acyl azolium cations have been proposed as immediate precursors of the ester product, for example, in the redox esterification of α,β‐enals. Studies with *d*_3_‐acetyl azolium triflate suggest that ester formation originates instead from an azolium enolate intermediate. Furthermore, the acetyl azolium enolate selectively reacted with alcohol nucleophiles in the presence of amines. While the acetyl azolium cation did not react with alcohols, an ester‐selective reaction was induced by addition of base, by intermediate formation of the acetyl azolium enolate.

## Graphical abstract

Acetyl azolium enolates resulted from the treatment of the N‐heterocyclic carbenes IPr and SIPr with 2‐propenyl acetate. Protonation converts them into acetyl azolium cations. The latter react with amines faster than with alcohols, while the azolium enolates reacted readily with alcohols, but sluggishly with amines. The use of a *d_3_*‐acetyl azolium triflate indicated that alcohol acetylation occurs at the azolium enolate stage.

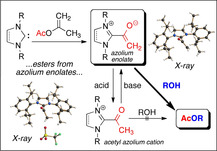

In recent years, the synthetic application of N‐heterocyclic carbenes (NHCs) has been extended beyond classical a^1^‐d^1^ umpolung of simple aldehydes:[^1^, ^2^, ^3^, ^4^] As shown in Scheme 1 a, a^3^‐d^3^‐umpolung of α,β‐unsaturated aldehydes with NHCs opens a pathway to homoenolate chemistry, through the formation of the diaminodienols **I**.^[5]^ Additionally, an OH‐C_γ_ proton shift in the diamino dienol **I** leads to the azolium enolate **II**. The latter behaves as an enolate equivalent and serves as the source of yet another broad spectrum of products.^[6]^

**Scheme 1:**
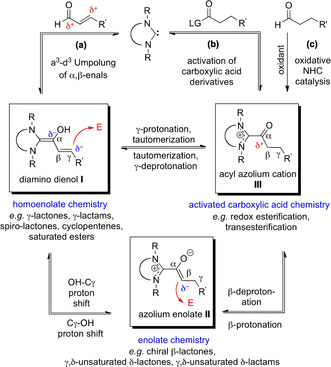
Manifold of intermediates formed from NHCs and α,β‐enals (a), activated carboxylic acid derivatives (b), through oxidative NHC catalysis (c), and their interconversion by proton transfer steps.

Besides applications in umpolung strategies, NHCs have also served as nucleophilic catalysts, for example, in the transesterification of activated esters.[^7^, ^8^, ^9^] As shown in Scheme 1 b, the acyl azolium cation **III** is believed to result from the interaction of the NHC catalyst with an activated carboxylic acid derivative. Note that a crossover exists between the two types of NHC‐catalysis shown in Scheme 1 a and b: γ‐protonation/tautomerization may equilibrate the diamino dienol **I** [pathway (a)] with the acyl azolium cation **III** [pathway (b)]. Another “cross‐over point” is the azolium enolate **II** which is accessible from both the diamino dienol **I** (by OH‐C_γ_ proton shift) and the acyl azolium cation **III** (by β‐deprotonation). Finally, the acyl azolium cation **III** can also be accessed by oxidative NHC catalysis, that is, from aldehydes in the presence of a suitable oxidant [Scheme 1, pathway (c)].^[10]^

In 2005, Scheidt et al.[^11a^, ^11b^, ^11c^] and Bode et al.[^11d^, ^11e^] reported the NHC‐catalyzed redox esterification of enals, leading from α,β‐unsaturated aldehydes to saturated esters (Scheme 2). The mechanism proposed by Scheidt and Bode for this transformation is shown in Scheme 2, pathway **A**: As a key step, γ‐protonation of the initially formed diamino dienol **I** affords the azolium enol **IV**. Tautomerization of the latter gives the acyl azolium cation **III**. Ester formation is completed by attack of the alcohol nucleophile on the latter. Note, however, that the occurrence of acyl azolium cations **III** in redox esterification has not been substantiated by isolation, or spectroscopically.^[11]^ We wondered whether ester formation may instead proceed through the azolium enolate stage (**II**, Scheme 2, pathway **B**). From **II**, a single‐step reaction with the alcohol component to the product ester can be formulated, with regeneration of the NHC catalyst.

**Scheme 2:**
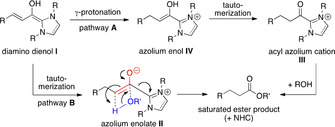
Mechanistic alternatives for the redox esterification of enals.

In 2012, Chi et al. exploited a reverse crossover and reported that NHC catalysis can be used for the generation of azolium enolates **II** from activated esters.^[12]^ After reaction with the NHC catalyst, β‐deprotonation of the initially formed acyl azolium cation **III** affords an azolium enolate **II** (Scheme 1 b). The latter can be reacted with various electrophiles, affording for example, γ,δ‐unsaturated δ‐lactams with N‐tosyl imines.^[12]^ Again, none of the intermediates postulated for such reverse crossover reactions had been characterized.

To probe the acyl transfer chemistry discussed above, we envisaged the acetate‐based azolium enolates **1**–**3 ae** (Scheme 3) and the acyl azolium cations **1,2 aa** (Scheme 4) as model systems. This choice was based on the simplicity of the acyl residue, and on our earlier experience that the use of SIPr, IPr as the carbene component provides sufficient stability for the characterization and even isolation of intermediates postulated for NHC catalysis. This approach had enabled us earlier to generate and probe Breslow intermediates involved in the NHC‐catalyzed umpolung of simple aldehydes,^[4]^ related intermediates of α,β‐enal umpolung,^[13]^ and even later stages of azolium enolate chemistry.^[13]^ In an elegant study by Maji and Mayr, azolium enolates have been prepared earlier by the reaction of NHCs with ketenes, and analyzed thoroughly with regard to their structure and reactivity towards benzhydrylium ions.^[14]^

**Scheme 3:**
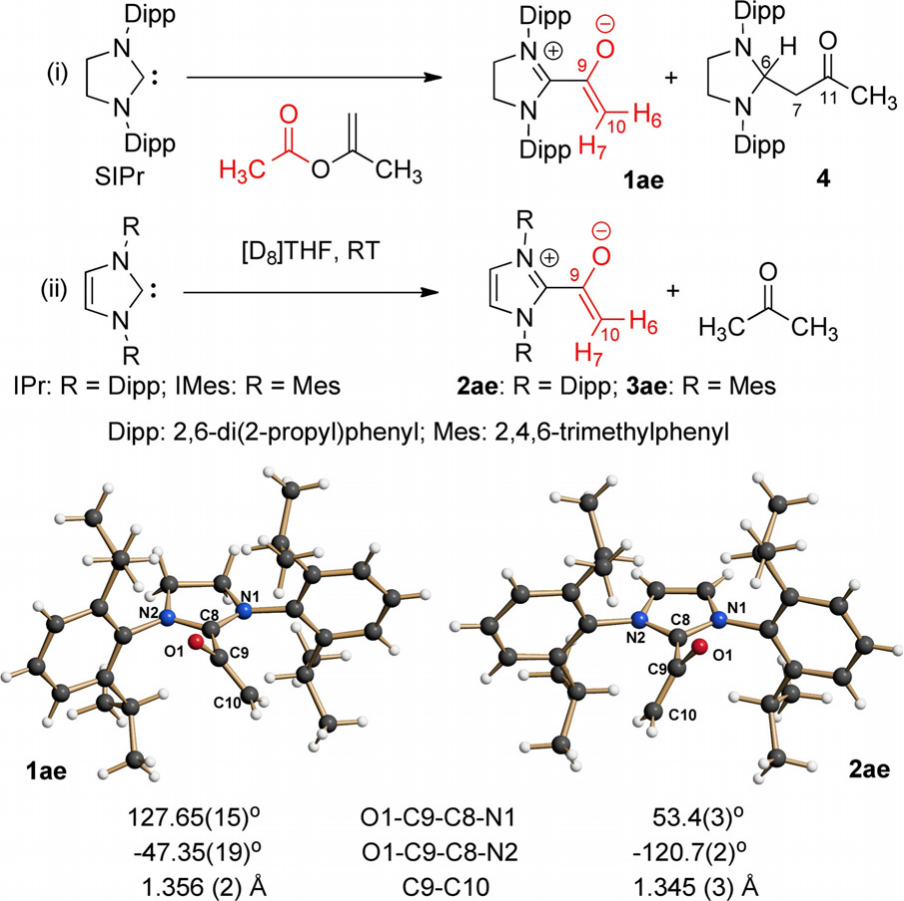
Preparation and X‐ray crystal structures of the azolium enolates **1**–**3 ae**.^[19]^

**Scheme 4:**
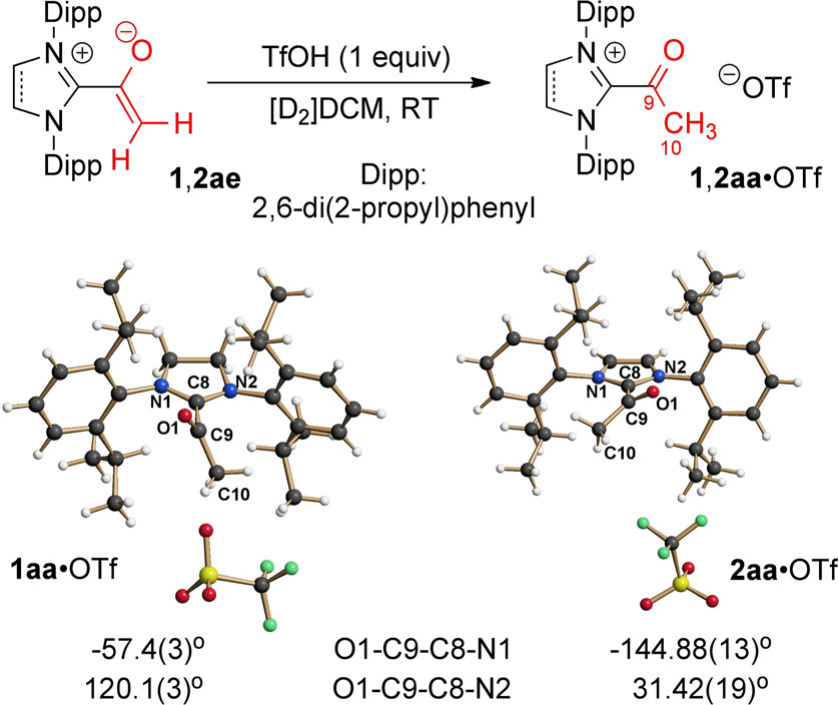
Preparation and X‐ray crystal structures of the acyl azolium triflates **1 aa**⋅OTf and **2 aa**⋅OTf.^[19]^

NMR monitoring revealed that the addition of 2‐propenyl acetate to a solution of the saturated imidazolidin‐2‐ylidene SIPr in [D_8_]THF at room temperature resulted in the smooth formation of the azolium enolate **1 ae**, together with the acetone adduct of SIPr, **4** as a 1:1 mixture [Scheme 3, (i)]. In [D_8_]THF, the azolium enolate **1 ae** displayed two characteristic singlets in its ^1^H NMR at *δ*=3.03 (s, 1 H, H6) and 2.65 (s, 1 H, H7), and ^13^C NMR resonances at *δ*=76.5 (C10) and 153.2 (C9) ppm. The azolium enolate **1 ae** crystallized from the reaction mixture, and single crystals suitable for X‐ray crystallography could be obtained (Scheme 3, bottom left).^[15]^ As a typical azolium enolate feature,[^13^, ^14^] the planar enolate moiety and the imidazolinium ring are tilted relative to one another, by ca. 47°. The C=C distance of the enolate moiety nicely reflects its double‐bond character [1.356(2) Å]. The SIPr‐acetone adduct **4** was clearly identified by NMR (see the Supporting Information). A control experiment revealed that the strongly basic^[16]^ NHC SIPr reacts smoothly with acetone in [D_8_]THF at RT, yielding exclusively the 1:1 adduct **4**.

When the unsaturated imidazolin‐2‐ylidene IPr was reacted with 2‐propenyl acetate [Scheme 3, (ii)] in [D_8_]THF at room temperature, acetone was liberated which, however, did not react further with IPr. Again, the azolium enolate **2 ae** crystallized from the reaction mixture. X‐ray diffraction (Scheme 3, bottom right) confirmed the constitution of the azolium enolate. The planar enolate moiety and the (planar) imidazolium ring are strongly tilted relative to one another, by ca. 53°, and the C=C distance [1.345(3) Å] within the enolate moiety proves its double bond character. The solubility of **2 ae** in [D_8_]THF turned out to be so low that the crystalline material had to be redissolved in [D_3_]MeCN for NMR characterization. In the latter solvent, **2 ae** displayed characteristic singlets in its ^1^H NMR at *δ*=3.09 ppm (s, 1 H, H6) and 2.75 ppm (s, 1 H, H7) and ^13^C NMR resonances at *δ*=77.7 (C10) and 132.7 (C9) ppm. When IMes was used as the NHC component, NMR analogously indicated the formation of the azolium enolate **3 ae** (see the Supporting Information). Unfortunately, no crystals of **3 ae** suitable for XRD could be obtained.

As summarized in Scheme 4, the acetyl azolium cations **1 aa** and **2 aa** were prepared, as triflates, from the azolium enolates **1 ae** and **2 ae** by protonation with trifluoromethanesulfonic acid (TfOH). In [D_2_]DCM, the instantaneous disappearance of the enolate proton resonances and appearance of a new singlet at *δ*=1.95 ppm (3 H, H10) in the ^1^H NMR, and of new ^13^C resonances at 186.4 (C9) and 29.3 (C10) ppm in the ^13^C NMR indicated the formation of the acetyl azolium salt **1 aa**⋅OTf. Its unsaturated counterpart **2 aa**⋅OTf shows almost identical new resonances. We succeeded in crystallizing both acetyl azolium triflates **1 aa**⋅OTf and **2 aa**⋅OTf, and their X‐ray crystal structures are shown in Scheme 4. In both cases, the acetyl moiety is again significantly tilted relative to the heterocyclic ring. In the case of the saturated acetyl azolium salt **1 aa**⋅OTf, the dihedral angle O1‐C9‐C8‐N1 amounts to ca. −57.4°, and somewhat smaller, yet significant, in the case of the aromatic azolium salt **2 aa**⋅OTf [O1‐C9‐C8‐N2 ca. 31.4°].

When exposed to benzyl alcohol (1 equiv) in [D_8_]THF (^1^H NMR observation) at RT, the azolium enolate **1 ae** was instantaneously converted to benzyl acetate (Scheme 5). The adduct of benzyl alcohol with SIPr (**5**) was formed as by‐product. The analogous reaction of **2 ae** with benzyl alcohol was studied in [D_2_]DCM, for solubility reasons. Again, ester formation was instantaneous, with IPr (as its DCl salt)^[17]^ being formed as by‐product.

**Scheme 5:**
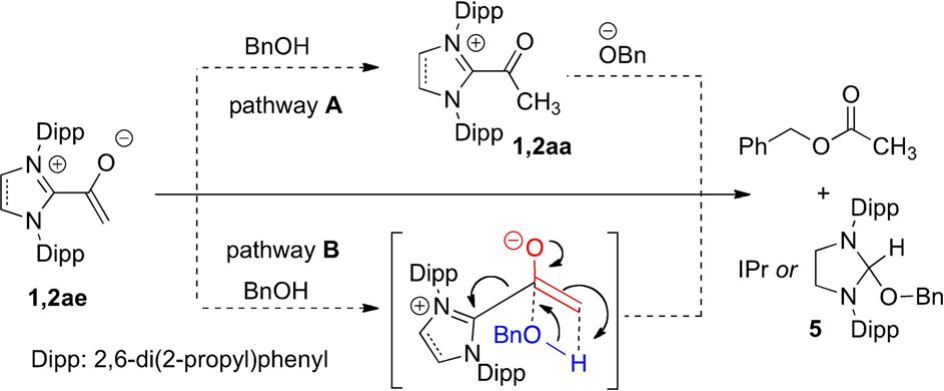
Mechanistic alternatives for the esterification of BnOH by the azolium enolates **1**,**2 ae**.

Mechanistically, the ester formation may proceed either by a discrete proton transfer from the alcohol to **1**,**2 ae**, affording the acyl azolium cation **1**,**2 aa** as intermediate (Scheme 5, pathway **A**). Our NMR monitoring did not indicate accumulation of any intermediate which, however, does not exclude this possibility, as the formation of **1**,**2 aa** may be rate‐limiting. Alternatively, a concerted proton/acyl‐transfer may be envisaged (Scheme 5, pathway **B**). When **1 ae** was exposed to BnOD instead of BnOH, a moderate kinetic isotope effect of ca. 1.4 was observed (see Tables S1 and S2 in the Supporting Information for *k*_H_/*k*_D_ data).

While neither one of the two results above allows a clear distinction, the following set of experiment advocates for the azolium enolate as the immediate ester precursor: We first established that in the absence of base, the acetyl azolium salt **1 aa**⋅OTf does not react with benzyl alcohol (or other alcohols). Stoichiometric addition of DBU, however, results in instantaneous ester formation. Again, it may be argued whether the base deprotonates the alcohol, or converts the acetyl azolium salt **1 aa**⋅OTf into the azolium enolate **1 ae** (as in Scheme 5). We addressed the latter question by using the trideuterated acetyl azolium triflate **1 aa**‐*d*_3_⋅OTf (Scheme 6). ^1^H NMR monitoring of this transformation clearly showed that in the resulting benzyl acetate, exactly one of the three acetyl deuterons had been exchanged for a proton (see the Supporting Information for ^1^H NMR spectral data). As depicted in Scheme 6, this formation of benzyl acetate‐*d*_2_ is compatible only with deprotonation of the acetyl azolium cation (to the azolium enolate **1 ae**‐*d*_2_; pathway **A**), and not with alcohol deprotonation (pathway **B**). In the latter case, full D‐retention, that is, formation of benzyl acetate‐*d*_3_ should have been expected. NMR monitoring showed that no concomitant H/D‐exchange occurs at the acetyl group's α‐position in the course of the ester formation. Another control experiment, in the absence of benzyl alcohol, confirmed that treatment with DBU cleanly and instantaneously converts the acetyl azolium triflate **1 aa**‐*d*_3_⋅OTf to the azolium enolate **1 ae**‐*d*_2_.

**Scheme 6:**
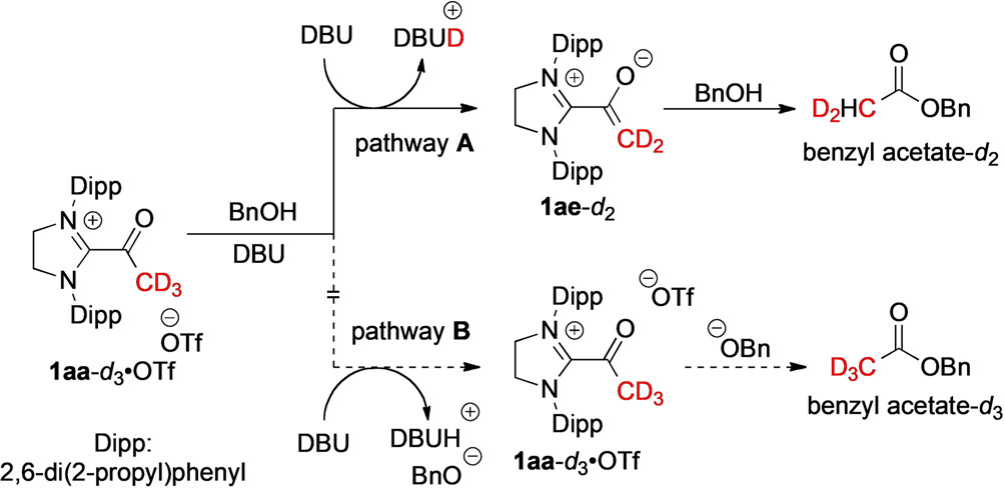
H/D‐Exchange in the esterification of BnOH by the acetyl azolium salt **1 aa‐*d***_**3**_⋅OTf, in the presence of DBU.

We conclude from the above studies that for the acetyl system **1**,**2 aa**⋅OTf/**1**,**2 ae**—and analogously for other acyl azolium ions carrying at least one α‐proton—ester formation most likely proceeds via the azolium enolate state. For the redox esterification of α,β‐enals, a modified, and in fact simplified mechanistic picture results (Scheme 7): In the first step, the diamino dienol **I** is generated from the substrate enal and the NHC catalyst. Tautomerization of the latter by OH‐C_γ_‐shift gives the azolium enolate **II** which can react directly with the alcohol component to the saturated ester product, with regeneration of the catalyst. We are well aware that this simple scheme does not explain the often complex influence of the nature and amount of base used for transforming azolium precatalysts to their active form. It is clear, however, that the equilibria NHC/NHC‐H^+^ and **I**/**II** alone bear sufficient potential for pronounced influence by acids and bases.

**Scheme 7:**
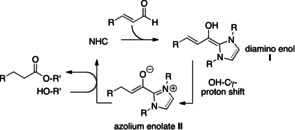
Simplified mechanistic proposal for the NHC‐catalyzed redox esterification of α,β‐enals.

In 2010, Studer et al. reported their intriguing observation that alcohols can selectively be cinnamoylated, in the presence of amines, under conditions of oxidative NHC‐catalysis.[^10^, ^18^, ^19^] With this in mind, we decided to evaluate the ester/amide selectivity of our azolium enolate/acetyl azolium pair **1 ae**/**1 aa**⋅OTf. We studied their reactivity towards benzyl alcohol (BnOH) and benzyl amine (BnNH_2_) by ^1^H NMR in [D_2_]DCM, the results are summarized in Table 1. Exposure of the azolium enolate **1 ae** to BnOH (entry 1) and BnNH_2_ (entry 2) resulted in smooth and quick ester formation, and sluggish amide formation, respectively. When **1 ae** was exposed to an equimolar mixture of BnOH and BnNH_2_, formation of benzyl acetate was favored by a factor of 5.5 over amidation (entry 3). Control experiments established that there is no secondary ester‐to‐amide transformation (see the Supporting Information). Therefore, the ester‐to‐amide ratio reflects the kinetic preference for esterification. On the basis of the proposed single‐step conversion of the azolium enolate (Scheme 5, pathway **B**), its preference for esterification can be explained by the higher acidity of RO−H versus RNH−H.

**Table 1:** Reactivity of the azolium enolate **1 ae** and of the acetyl azolium triflate **1 aa**⋅OTf towards BnOH and BnNH_2_.

Entry	Reagent	Nucleophile	Ester/ amide
1^[a]^	**1 ae**	BnOH (1.5 equiv)	100:0
2^[b]^	**1 ae**	BnNH_2_ (1.5 equiv)	0:100
3^[c]^	**1 ae**	BnOH : BnNH_2_ (1:1)	5.5:1
4	**1 aa**⋅OTf	BnOH (1.5 equiv)	No reaction
5^[d]^	**1 aa**⋅OTf	BnNH_2_ (1.5 equiv)	0:100
6^[e]^	**1 aa**⋅OTf	BnOH (1.5 equiv) DBU (1 equiv)	100:0
7^[c]^	**1 aa**⋅OTf	BnOH : BnNH_2_ (1:1) DBU (1 equiv)	5.5:1
8^[c,f]^	**1 aa**⋅OTf	BnOH : BnNH_2_ (1:1)	1:8

Reaction conditions: 0.023 mmol of **1 ae**/**1 aa**⋅OTf, 0.034 mmol of BnOH/ BnNH_2_, 0.5 mL [D_2_]DCM, 18 h at RT. [a] Full conversion was observed after 7 h. [b] 28 % conversion was observed after 18 h. [c] 0.023 mmol of BnOH and BnNH_2_ each. [d] 86 % Conversion was observed after 18 h. [e] Full conversion was observed after 6 h. [f] 82 % Conversion was observed after 18 h.

In contrast, the reactivity pattern of the acetyl azolium triflate **1 aa**⋅OTf is simply that of an activated carboxylic acid derivative. In line with earlier observations by Studer et al.,^[18]^ **1 aa**⋅OTf did not react with BnOH (Table 1, entry 4), while exposure of **1 aa**⋅OTf to BnNH_2_ resulted in instantaneous formation of BnNHAc (entry 5). When the acetyl azolium salt **1 aa**⋅OTf was treated with benzyl alcohol in the presence of DBU (entry 6), rapid conversion to the ester occurred. When exposed to an equimolar mixture of BnOH and BnNH_2_, in the presence of DBU (entry 7), exactly the same ester‐to‐amide ratio resulted as it was found before for the azolium enolate **1 ae** (entry 3). This result is in line with our earlier conclusion that in the presence of base, the acetyl azolium cation **1 aa** is first deprotonated to the azolium enolate **1 ae**, and that ester/amide formation proceed from the latter (Scheme 6). In the absence of DBU, an amide‐to‐ester ratio of 8:1 was observed (Table 1, entry 8). As no free SIPr results in the course of the amidation/esterification of **1 ae** (instead, the imidazolinium triflate of SIPr), the ester formation may be promoted by H‐bonding of the alcohol to the amine.[^18^, ^19^] Studer et al. also reported that for ester‐over‐amide selective acetylation, 2‐propenyl acetate can be used with IMes as catalyst, and the corresponding acetyl azolium cation was proposed as reactive intermediate.^[18b]^ However, according to our results (Scheme 3), also the IMes‐catalysis of ester formation from alcohols and 2‐propenyl acetate should proceed via the azolium enolate **3 ae**.

In summary, several acetyl azolium enolates and azolium triflates have been prepared, characterized, and employed to probe the mechanism of ester formation in NHC catalyzed transformations. Our study shows that these azolium enolates react readily with alcohols and suggests that ester formation in fact proceeds via the azolium enolate stage. Furthermore, the azolium enolate studied showed pronounced ester‐over‐amide selectivity. The selectivity observed in NHC catalyzed acetylations with vinyl acetates thus corresponds to the selectivity of the primarily formed acetyl azolium enolate. We finally wish to reiterate that for stability reasons, our study was carried out with Dipp/Mes‐substituted imidazole/imidazoline based azolium enolates and acyl azolium cations. NHC catalysts applied in practical synthesis are typically of different, for example, triazolium, types. It appears reasonable to assume that the conclusions drawn here do apply to the latter classes of NHC catalysts as well. Nevertheless, mechanistic analysis will ultimately be required in each individual case to scrutinize this assumption.

## Conflict of interest

The authors declare no conflict of interest.

## Supporting information

As a service to our authors and readers, this journal provides supporting information supplied by the authors. Such materials are peer reviewed and may be re‐organized for online delivery, but are not copy‐edited or typeset. Technical support issues arising from supporting information (other than missing files) should be addressed to the authors.

Supplementary
