# Supplementary material for: Acyl Donor Intermediates in N‐Heterocyclic Carbene Catalysis: Acyl Azolium or Azolium Enolate?
Source: Angew Chem Int Ed Engl. 2021 Jan 18;60(9):4507–11. doi: 10.1002/anie.202010348 (PMC7986403; doi:10.1002/anie.202010348)
Supplement: Supplementary file 1 — Supplementary [file ANIE-60-4507-s001.pdf]

Angewandte Chemie
Eine Zeitschrift der Gesellschaft Deutscher Chemiker

Supporting Information

Acyl Donor Intermediates in N-Heterocyclic Carbene Catalysis: Acyl Azolium or Azolium Enolate?

*Animesh Biswas, Jörg-M. Neudörfl, Nils E. Schlörer, and Albrecht Berkessel**

anie_202010348_sm_miscellaneous_information.pdf

Author Contributions

J.-M. Neudörfl: X-Ray Crystallography; N. E. Schlörer: NMR Spectroscopy.

Table of Contents

1 General……………………………………………………………….……………………………………….4
2 Interaction of SIPr with acetone……………………………………………………………………………….5
3 Interaction of IPr with acetone………………………………….…………………………………….....5
4 Azolium enolates……………………………………..……………………………………………………….6
4.1 Preparation of {1,3-bis[2,6-di(propan-2-yl)phenyl]-4,5-dihydro-1*H*-imidazol-3-ium-2-yl}ethenolate **1ae**……………………………………………………………………………………………..6
4.2 Preparation of {1,3-bis[2,6-di(propan-2-yl)phenyl]-1*H*-imidazol-3-ium-2-yl}ethenolate **2ae**………6
4.3 Preparation of {1,3-bis(2,4,6-trimethylphenyl)-1*H*-imidazol-3-ium-2-yl}ethenolate **3ae**………..….7
5 Acyl azolium triflates……………..…………………….………………………………………………..8
5.1 Preparation of 1-{1,3-bis[2,6-di(propan-2-yl)phenyl]-4,5-dihydro-1*H*-imidazol-3-ium-2-yl}-ethanone trifluoromethanesulfonate. **1aa**•OTf...8
5.2 Preparation of 1-{1,3-bis[2,6-di(propan-2-yl)phenyl]-1*H*-imidazol-3-ium-2-yl}-ethanone trifluoromethanesulfonate **2aa**•OTf……………...8
6 NMR studies of ester formation from azolium enolates ……………………………………………….....9
6.1 Reaction of the azolium enolate **1ae** with 8 equiv. of benzyl alcohol………………………………...9
6.2 Reaction of the azolium enolate **1ae** with 8 equiv. of deuterated benzyl alcohol…………………11
7 NMR studies and reaction kinetics of ester formation from acyl azolium triflates in the presence of DBU…13
7.1 Reaction of the deuterated acyl azolium salt **1aa-*d*$_3$**•OTf with 1.5 equiv. of benzyl alcohol in the presence of DBU ……………………………………………………………………………….....13
7.2 Generation of the azolium enolate **1ae** from the acyl azolium triflate **1aa**•OTf by treatment with DBU………………………………………………………………………………..……………...15
8 NMR studies on the ester/amide selectivity of azolium enolate **1ae** and acyl azolium triflate **1aa.OTf**…….16
8.1 Reaction of the azolium enolate **1ae** with (1:1) equiv. of benzyl alcohol and benzylamine…..........17
8.2 Reaction of the acyl azolium triflate **1aa.OTf** with (1:1) equiv. of benzyl alcohol and benzylamine.18
9 Control experiment for secondary ester-to-amide transformation……………………………………………19
10 NMR Spectra……………………………………………………………………………………………………21
10.1 1D and 2D NMR spectra of 1-(1,3-bis(2,6-diisopropylphenyl)imidazolidin-2-yl)propan-2-one **4**...21
10.2 1D and 2D NMR spectra of 1-{1,3-bis[2,6-di(propan-2-yl)phenyl]-4,5-dihydro-1*H*-imidazol-3-ium-2-yl}-ethenolate **1ae**………………………...24
10.3 1D and 2D NMR spectra of {1,3-bis[2,6-di(propan-2-yl)phenyl]-1*H*-imidazol-3-ium-2-yl}ethenolate **2ae**……………………………………………..27
10.4 1D and 2D NMR spectra of {1,3-bis(2,4,6-trimethylphenyl)-1*H*-imidazol-3-ium-2-yl}ethenolate **3ae**……………………………………………………………………………………………………30
10.5 1D and 2D NMR spectra of 1-{1,3-bis[2,6-di(propan-2-yl)phenyl]-4,5-dihydro-1*H*-imidazol-3-ium-2-yl}-ethanone trifluoromethanesulfonate **1aa**•OTf………………………………………………...33
10.6 1D and 2D NMR spectra of 1-{1,3-bis[2,6-di(propan-2-yl)phenyl]-4,5-dihydro-1*H*-imidazol-3-ium-2-yl}-ethanone trifluoromethanesulfonate **2aa**•OTf…………………………………….................37
10.7 ^{1}H NMR of deuterated benzyl alcohol…………………………………………………………………40

11 X-ray data: Crystal data and structure refinement, ORTEPs……………………………………………………41

11.1 X-ray data of 1-{1,3-bis[2,6-di(propan-2-yl)phenyl]-4,5-dihydro-1*H*-imidazol-3-ium-2-yl}-ethenolate **1ae**……………………………………………………………………………………………41

11.2 X-ray data of {1,3-bis[2,6-di(propan-2-yl)phenyl]-1*H*-imidazol-3-ium-2-yl}ethenolate **2ae**……….43

11.3 X-ray data of 1-{1,3-bis[2,6-di(propan-2-yl)phenyl]-4,5-dihydro-1*H*-imidazol-3-ium-2-yl}-ethanone trifluoromethanesulfonate **1aa**•OTf..…………………………………………………………………….45

11.4 X-ray data of of 1-{1,3-bis[2,6-di(propan-2-yl)phenyl]-4,5-dihydro-1*H*-imidazol-3-ium-2-yl}-ethanone trifluoromethanesulfonate **2aa**•OTf……..47

12 References………………………………………………………………………...49

1 General

1,3-Bis(2,6-diisopropylphenyl)imidazolidin-2-ylidene (>98.0 %, SIPr) and 1,3-bis(2,6-diisopropylphenyl)imidazolin-2-ylidene (>98.0 %, IPr) were purchased from TCI. 1,3-Bis(2,4,6-trimethylphenyl)imidazolin-2-ylidene (97.0 %, IMes). Iso-propenyl acetate was purchased from Acros Organics. Deuterated iso-propenyl acetate was provided by BASF SE, Ludwigshafen. Aldehydes, iso-propenyl acetate, amines and alcohols were distilled and stored in a glovebox. [D_8]THF and [D_2]DCM were passed through neutral aluminum oxide (Brockmann activity 1), degassed by several freeze-pump-thaw cycles and stored over 4 Å molecular sieves in a glovebox. N-Benzylacetamide,[1] benzyl acetate[2] and deuterated benzyl alcohol[3] were prepared according to literature procedures. All reactions were performed under argon atmosphere in a glovebox.

Nuclear magnetic resonance (NMR) spectra were recorded on a Bruker Avance II 600 instrument (^{1}H: 600.20 MHz, ^{13}C: 150.92 MHz), on a Bruker AV 400 instrument (^{1}H: 400.13 MHz, ^{13}C: 100.61 MHz), or on a Bruker AV 300 instrument (^{1}H: 300.13 MHz, ^{13}C: 75.46 MHz, ^{19}F: 282.404 MHz). Spectra were recorded at room temperature unless otherwise stated. Chemical shifts (δ) are reported in parts per million relative to tetramethyl silane (TMS) or solvent residual signals. In case of ^{19}F NMR, chemical shifts (δ) are reported in parts per million relative to $CFCl_3$. The following abbreviations were used for chemical shift multiplicities in ^{1}H NMR spectra: brs = broad singlet, s = singlet, d = doublet, t = triplet, q = quartet, sept = septet, m = multiplet. NMR signals were assigned based on of 1D and 2D NMR data (^{1}H,^{1}H COSY, ^{1}H,^{1}H NOESY, ^{1}H,^{13}C HSQC, ^{1}H,^{13}C HMBC). Reaction kinetics data were analyzed by Dynamics Center 2.5 (https://www.bruker.com/products/mr/nmr/software/dynamics-center.html). Melting points were measured on a Büchi 535 apparatus in a sealed capillary (samples were prepared and sealed in a glovebox) and the values are uncorrected.

2 Interaction of SIPr with acetone

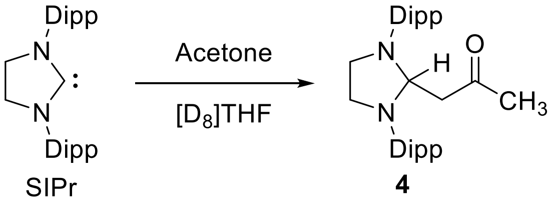

In a glovebox, an NMR tube was charged with 30 mg (76 µmol, 1.0 equiv) of SIPr in [D$_8$]THF (0.5 mL) and sealed with a septum. Acetone (1.0 equiv, 4.46 mg, 5.6 µL) was added with a syringe and the reaction was followed using ^{1}H NMR spectroscopy at room temperature. Clean formation of the SIPr-acetone adduct **4** was observed in an overnight measurement at RT.

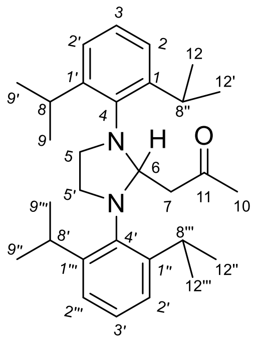

4

^{1}H NMR (600 MHz, [D$_8$]THF): δ = 7.23-7.19 (m, 2H, H3, H3’), 7.18-7.13 (m, 4H, H2, H2’, H2’’, H2’’’), 5.42 (t, 1H, $^3J_{HH}$ = 5.2 Hz, H6), 3.88 (sept, 2H, $^3J_{HH}$ = 6.9 Hz, H8, H8’), 3.73-3.7 (m, 2H, H5, H5’), 3.62-3.59 (m, 2H, H5, H5’), 3.47 (sept, 2H, $^3J_{HH}$ = 6.9 Hz, H8’’, H8’’’), 2.38 (d, 2H, $^3J_{HH}$ = 5.2 Hz, H7),1.39 (d, 6H, $^3J_{HH}$ = 6.9 Hz, H12, H12’), 1.38 (s, 3H, H10), 1.34 (d, 6H, $^3J_{HH}$ = 6.9 Hz, H9, H9’), 1.28 (d, 6H, $^3J_{HH}$ = 6.9 Hz, H12’’, H12’’’), 1.25 (d, 6H, $^3J_{HH}$ = 6.9 Hz, H9’’, H9’’’) ppm. **^{13}C NMR (150 MHz, [D$_8$]THF):** δ = 204.0 (1C, C11), 150.6 (2C, C8’’, C8’’’), 150.1 (2C, C8, C8’), 139.1 (2C, C4, C4’), 127.1 (2C, C3, C3’), 124.3 (2C, C1, C1’’), 123.4 (2C, C1’, C1’’’), 77.6 (1C, C6) , 51.8 (2C, C5, C5’), 48.2 (1C, C7), 29.4 (1C, C10), 28.7 (2C, C8, C8’), 27.8 (2C, C8’’, C8’’’), 25.6 (2C, C9’’, C9’’’), 24.8 (2C, C12’’, C12’’’), 22.9 (2C, C12, C12’), 22.7 (2C, C9, C9’) ppm.

3 Interaction of IPr with acetone

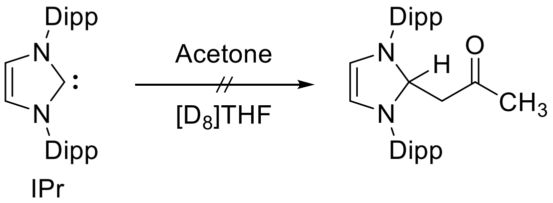

In a glovebox, an NMR tube was charged with 14.5 mg (37 µmol, 1.0 equiv) of IPr in [D$_8$]THF (0.5 mL) and sealed with a septum. Acetone (1.0 equiv, 2.1 mg, 2.7 µL) was added with a syringe and the reaction was followed using ^{1}H NMR spectroscopy at room temperature. No consumption of the starting materials was observed in an overnight measurement at RT.

4 Azolium enolates

4.1 Preparation of {1,3-bis[2,6-di(propan-2-yl)phenyl]-4,5-dihydro-1*H*-imidazol-3-ium-2-yl}ethenolate 1ae

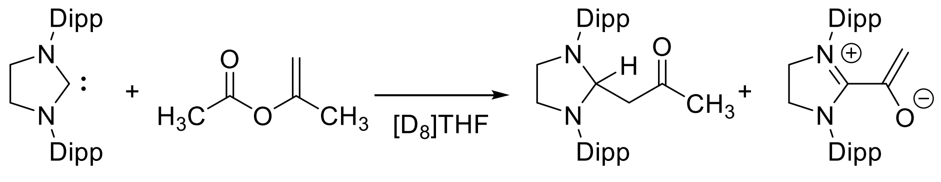

SIPr **4** **1ae**

In a glovebox, an NMR tube was charged with 30 mg (76 µmol, 1.0 equiv) of SIPr in $[D_8]$THF (0.5 mL) and sealed with a septum. Iso-propenyl acetate (0.5 equiv, 4.2 µL) was added with a syringe and the reaction was followed using ^{1}H NMR spectroscopy at room temperature. The color changes from colorless to yellow. After 8 h at RT, yellow crystals were observed inside the NMR tube. In a glovebox, the crystals were filtered off from the solution and washed with $[D_8]$THF to obtain 14 mg (32 µmol, 42%) of **1ae** as yellow crystals.

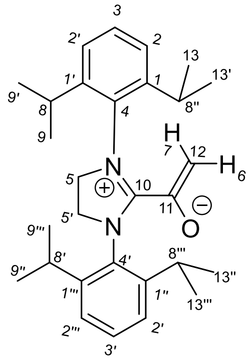

1ae

1ae: {1,3-Bis[2,6-di(propan-2-yl)phenyl]-4,5-dihydro-1*H*-imidazol-3-ium-2-yl} ethenolate.

mp.: decomposition at 198-199 °C; **^{1}H NMR (600 MHz, $[D_8]$THF):** δ = 7.34-7.30 (m, 2H, H3, H3’), 7.23-7.20 (m, 4H, H2, H2’, H2’’, H2’’’), 4.15 (s, 4H, H5, H5’), 3.26 (sept, 4H, $^3J_{HH}$ = 6.9 Hz, H8, H8’, H8’’, H8’’’), 3.03 (s, 1H, H6), 2.65 (s, 1H, H7), 1.41 (d, 12H, $^3J_{HH}$ = 6.9 Hz, H9, H9’, H9’’, H9’’’), 1.32 (d, 12H, $^3J_{HH}$ = 6.9 Hz, H13, H13’, H13’’, H13’’’) ppm. **^{13}C NMR (150 MHz, $[D_8]$THF):** δ = 172.4 (1C, C10), 153.2 (1C, C11), 146.2 (4C, C1, C1’, C1’’, C1’’’), 133.7 (2C, C4, C4’), 128.8 (2C, C3, C3’), 124.0 (4C, C2, C2’, C2’’, C2’’’), 76.5 (1C, C12), 52.0 (2C, C5, C5’), 28.8 (4C, C8, C8’, C8’’, C8’’’), 24.6 (4C, C13, C13’, C13’’, C13’’’), 23.5 (4C, C9, C9’, C9’’, C9’’’) ppm.

4.2 Preparation of {1,3-bis[2,6-di(propan-2-yl)phenyl]-1*H*-imidazol-3-ium-2-yl}ethenolate 2ae

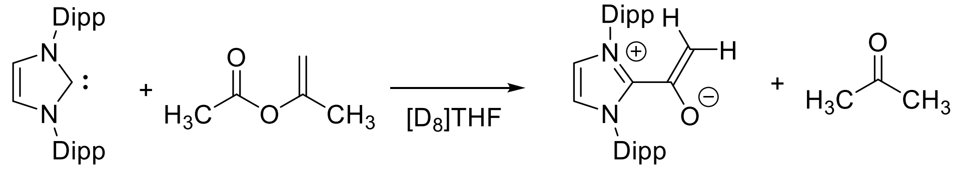

IPr **2ae**

In a glovebox, an NMR tube was charged with 17.5 mg (45 µmol, 1.0 equiv.) of IPr in $[D_8]$THF (0.5 mL) and sealed with a septum. Iso-propenyl acetate (1 equiv, 4.9 µL) was added with a syringe and the reaction was followed using ^{1}H NMR spectroscopy at room temperature. The color changes from colorless to yellow. After 30 min yellow crystals were observed inside the NMR tube. In a glovebox, the crystals were isolated from the solution and washed with $[D_8]$THF to obtain 19 mg (44 µmol, 98%) of **2ae** as yellow crystalline needles.

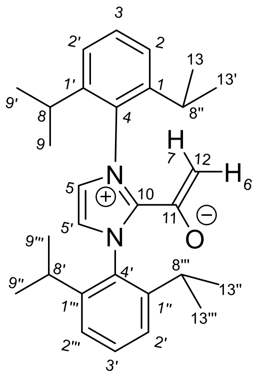

2ae

2ae: {1,3-Bis[2,6-di(propan-2-yl)phenyl]-1*H*-imidazol-3-ium-2-yl}ethenolate.

mp.: decomposition at 224-225 °C; **^{1}H NMR (400 MHz, [D$_3$]MeCN):** δ = 7.53-7.51 (m, 2H, H3, H3'), 7.43-7.31 (m, 6H, H5, H5', H2, H2', H2'', H2'''), 3.09 (s, 1H, H6), 2.75 (s, 1H, H7), 2.67 (sept, 4H, $^3J_{HH}$ = 6.9 Hz, H8, H8', H8'', H8'''), 1.32 (d, 12H, $^3J_{HH}$ = 6.8 Hz, H9, H9', H9'', H9'''), 1.20 (d, 12H, $^3J_{HH}$ = 6.9 Hz, H13, H13', H13'', H13''') ppm. **^{13}C NMR (100 MHz, [D$_3$]MeCN):** δ = 153.0 (1C, C10), 145.6 (6C, C1, C1', C1'', C1''', C4, C4'), 132.7 (1C, C11), 130.4 (2C, C3, C3'), 124.0 (4C, C2, C2', C2'', C2'''), 122.3 (2C, C5, C5'), 77.7 (1C, C12), 28.9 (4C, C8, C8', C8'', C8'''), 24.1 (4C, C13, C13', C13'', C13'''), 22.3 (4C, C9, C9', C9'', C9''') ppm.

4.3 Preparation of {1,3-bis(2,4,6-trimethylphenyl)-1*H*-imidazol-3-ium-2-yl}ethenolate 3ae

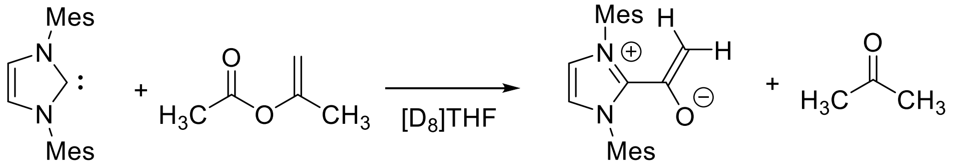

IMes **3ae**

In a glovebox, an NMR tube was charged with 20 mg (65 µmol, 1.0 equiv.) of IMes in [D$_8$]THF (0.6 mL) and sealed with a septum. Iso-propenyl acetate (1 equiv, 7.2 µL) was added with a syringe and the reaction was followed using ^{1}H NMR spectroscopy at room temperature. After 10 min, a white precipitate was observed inside the NMR tube. In a glovebox, the precipitate was filtered and washed with [D$_8$]THF to obtain 20 mg (57.7 µmol, 88%) of **3ae** as white solid.

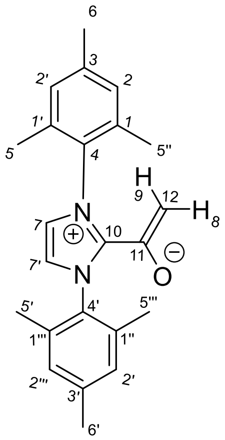

3ae

3ae: {1,3-Bis(2,4,6-trimethylphenyl)-1*H*-imidazol-3-ium-2-yl}ethenolate.

mp.: decomposition at 208-209 °C; **^{1}H NMR (600 MHz, [D$_3$]MeCN):** δ = 7.27 (s, 2H, H7, H7'), 7.04 (s, 4H, H2, H2', H2'', H2'''), 2.96 (s, 1H, H8), 2.76 (s, 1H, H9), 2.31 (s, 6H, H6, H6'), 2.16 (s, 12H, H5, H5', H5'', H5''') ppm. **^{13}C NMR (150 MHz, [D$_3$]MeCN):** δ = 154.8 (1C, C10), 153.0 (1C, C11), 140.9 (2C, C3, C3'), 136.3 (4C, C1, C1', C1'', C1'''), 133.9 (2C, C4, C4'), 129.9 (4C, C2, C2', C2'', C2'''), 122.3 (2C, C7, C7'), 76.7 (1C, C12), 21.1 (2C, C6, C6'), 17.9 (4C, C5, C5', C5'', C5''') ppm.

5 Acyl azolium triflates

5.1 Preparation of 1-{1,3-bis[2,6-di(propan-2-yl)phenyl]-4,5-dihydro-1*H*-imidazol-3-ium-2-yl}-ethanone trifluoromethanesulfonate 1aa•OTf

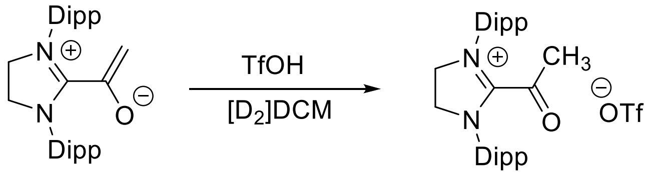

1ae **1aa**•OTf

In a glovebox, the azolium enolate **1ae** was generated and crystallized as described before. An NMR tube was charged with 7 mg (16 µmol, 1.0 equiv.) of **1ae** in [D_2]DCM (0.5 mL) and sealed with a septum. Trifluoromethanesulfonic acid (1 equiv,1.4 µL) was added with a syringe. The color of the solution changed from yellow to light yellow instantaneously and the quantitative formation of the acyl azolium salt **1aa**•OTf was observed by NMR spectroscopy. The contents of the NMR tube were poured into a small open vial, 0.5 mL THF was added, and left for slow evaporation of the solvent. After a day, colorless crystals had formed.

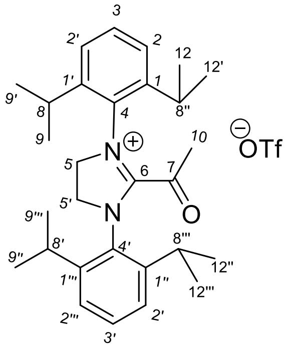

1aa•OTf

1aa•OTf: 1-{1,3-Bis[2,6-di(propan-2-yl)phenyl]-4,5-dihydro-1*H*-imidazol-3-ium-2-yl}-ethanone trifluoromethanesulfonate.

mp.: 289-290 °C; **^{1}H NMR (600 MHz, [D_2]DCM):** δ = 7.55 (t, $^3J_{HH}$ = 7.8 Hz, 2H, H3, H3’), 7.34 (d, $^3J_{HH}$ = 7.8 Hz, 4H, H2, H2’, H2’’, H2’’’), 4.66 (s, 4H, H5, H5’), 3.00 (sept, 4H, $^3J_{HH}$ = 6.8 Hz, H8, H8’, H8’’, H8’’’), 1.95 (s, 3H, H10), 1.39 (d, 12H, $^3J_{HH}$ = 6.7 Hz, H9, H9’, H9’’, H9’’’), 1.34 (d, 12H, $^3J_{HH}$ = 6.8 Hz, H12, H12’, H12’’, H12’’’) ppm. **^{13}C NMR (150 MHz, [D_2]DCM):** δ = 186.4 (1C, C7), 161.3 (1C, C6), 146.15 (4C, C1, C1’, C1’’, C1’’’), 132.1 (2C, C4, C4’), 128.2 (2C, C3, C3’), 125.7 (6C, C4, C4’, C4’’, C4’’’, C2, C2’, C2’’, C2’’’), 54.5 (2C, C5, C5’), 29.4 (4C, C8, C8’, C8’’, C8’’’), 29.3 (1C, C10), 25.7 (4C, C12, C12’, C12’’, C12’’’), 23.0 (4C, C9, C9’, C9’’, C9’’’) ppm. **^{19}F NMR (282 MHz, [D_2]DCM) :** δ = −78.92 (OTf) ppm.

5.2 Preparation of 1-{1,3-bis[2,6-di(propan-2-yl)phenyl]-1*H*-imidazol-3-ium-2-yl}-ethanone trifluoromethanesulfonate 2aa•OTf

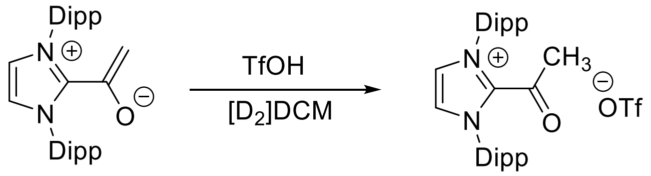

2ae **2aa**•OTf

In a glovebox, the azolium enolate **2ae** was generated and crystallized as described before. An NMR tube was charged with 7 mg (16 µmol, 1.0 equiv.) of **2ae** in [D$_2$]DCM (0.5 mL) and sealed with a septum. Trifluoromethanesulfonic acid (1 equiv, 1.43 µL) was added with a syringe. The color of the solution changed to light yellow instantaneously and the quantitative formation of the acyl azolium salt **2aa•**OTf was observed by NMR spectroscopy. The contents of the NMR tube were poured into a small open vial, 0.5 mL THF was added, and left for slow evaporation of the solvent. After a day, colorless crystals had formed.

2aa•OTf: 1-{1,3-Bis[2,6-di(propan-2-yl)phenyl]-1*H*-imidazol-3-ium-2-yl}-ethanone trifluoromethanesulfonate.

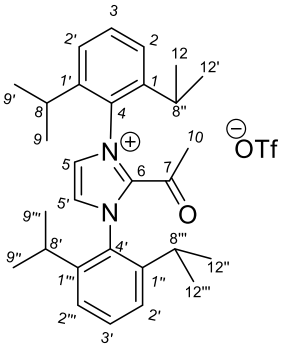

2aa•OTf

mp.: 210-211 °C; **^{1}H NMR (600 MHz, [D$_2$]DCM):** δ = 8.09 (s, 2H, H5, H5'), 7.69 (t, $^3J_{HH}$ = 7.80 Hz, 2H, H3, H3'), 7.45 (d, 4H, $^3J_{HH}$ = 8.21 Hz, H2, H2', H2'', H2'''), 2.29 (sept, 4H, $^3J_{HH}$ = 6.91 Hz, H8, H8', H8'', H8'''), 1.92 (s, 3H, H10), 1.29 (d, 12H, $^3J_{HH}$ = 6.81 Hz, H9, H9', H9'', H9'''), 1.22 (d, 12H, $^3J_{HH}$ = 7.09 Hz, H12, H12', H12'', H12''') ppm. **^{13}C NMR (150 MHz, [D$_2$]DCM):** δ = 182.1 (1C, C7), 144.3 (4C, C1, C1', C1'', C1'''), 139.0 (1C, C6), 132.7 (2C, C3, C3'), 130.2 (2C, C4, C4'), 128.6 (2C, C5, C5'), 125.4 (4C, C2, C2', C2'', C2'''), 29.4 (4C, C8, C8', C8'', C8'''), 29.5 (5C, C10, C8, C8', C8'', C8'''), 24.5 (4C, C12, C12', C12'', C12'''), 22.8 (4C, C9, C9', C9'', C9''') ppm. **^{19}F NMR (282 MHz, [D$_2$]DCM) :** δ = −79.06 (OTf) ppm.

6 NMR studies of ester formation from azolium enolates

6.1 Reaction of the azolium enolate 1ae with 8 equiv. of benzyl alcohol

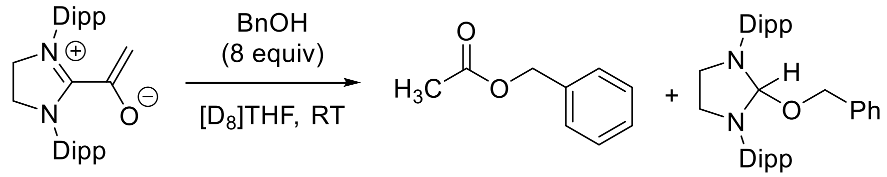

1ae **5**

In a glovebox, the azolium enolate **1ae** was generated and crystallized as described before. An NMR tube was charged with 7 mg (16 µmol, 1.0 equiv.) of **1ae** in [D$_8$]THF (0.5 mL) and sealed with a septum. Benzyl alcohol (8 equiv, 13.5 µL) was added with a syringe and the reaction was followed by ^{1}H NMR spectroscopy. The formation of benzyl acetate and of the SIPr-alcohol adduct **5** was observed instantaneously.

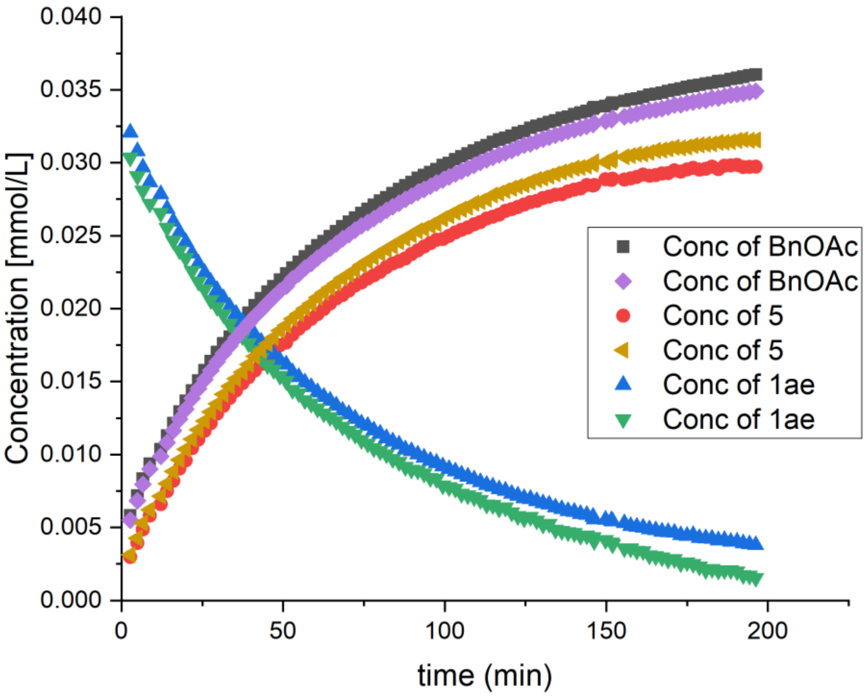

Figure S1. Concentration vs time profile of the ester formation from **1ae** in the presence of 8 equiv. of BnOH.

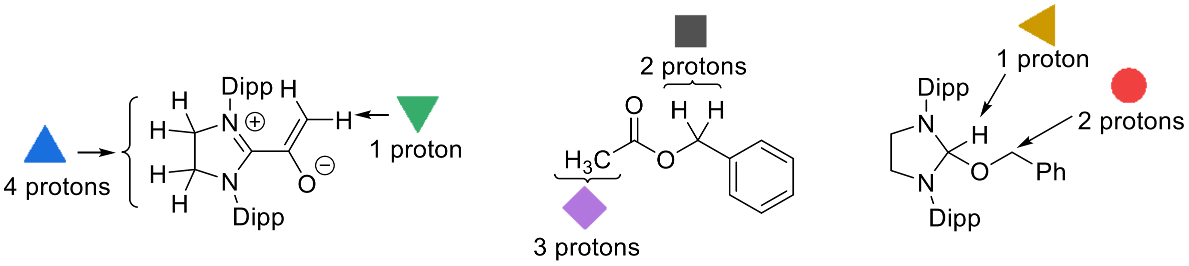

1ae Benzyl acetate **5**

Figure S2. Protons integrated for monitoring the reaction kinetics and rate constant (k_H) values.

Table S1. Rate constant (k_H) values for each component (under pseudo first order condition).

Peak designation	δ [ppm]	Rate constant, k_H [1/min]	Error [1/min]
**BnOAc**, ■	5.09	1.46E-2	6E-5
**BnOAc**, ◆	2.05	1.46E-2	3E-5
**5**, ●	6.64	1.50E-2	8E-5
**5**, ◀	5.55	1.49E-2	1E-4
**1ae**, ▲	4.22	1.56E-2	4E-5
**1ae**, ▼	3.04	1.39E-2	7E-5

6.2 Reaction of the azolium enolate 1ae with 8 equiv. of deuterated benzyl alcohol

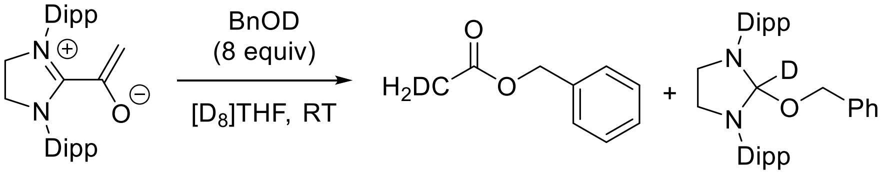

In a glovebox, the azolium enolate **1ae** was generated and crystallized as described before. An NMR tube was charged with 7.5 mg (17 µmol, 1.0 equiv.) of **1ae** in [D8]THF (0.5 mL) and sealed with a septum. Deuterated benzyl alcohol (8 equiv, 14.5 µL) was added with a syringe and the reaction was followed by ^{1}H NMR spectroscopy. The formation of deuterated benzyl acetate and of the SIPr-alcohol adduct **5-*d*** was observed instantaneously.

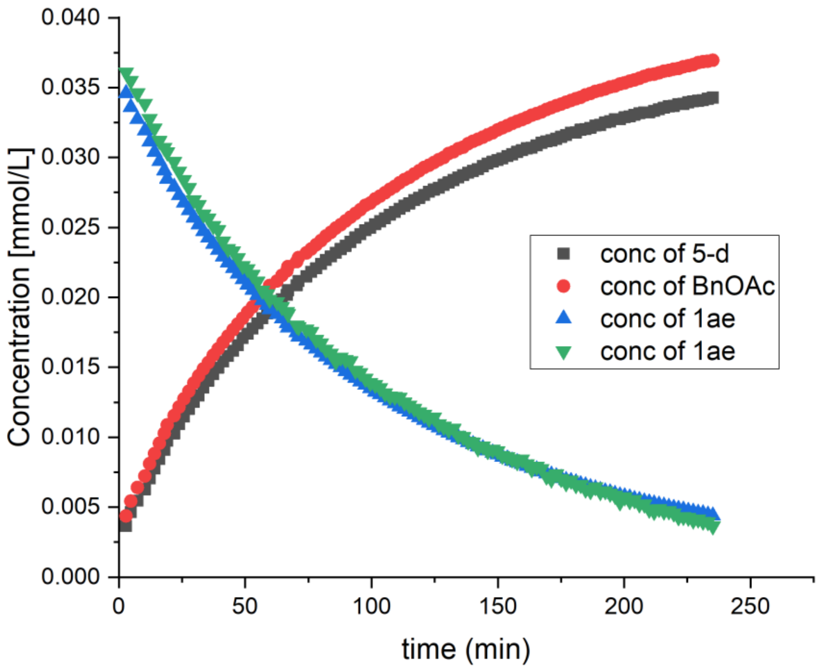

Figure S3. Concentration vs time profile of the ester formation from **1ae** in the presence of 8 equiv. of BnOD.

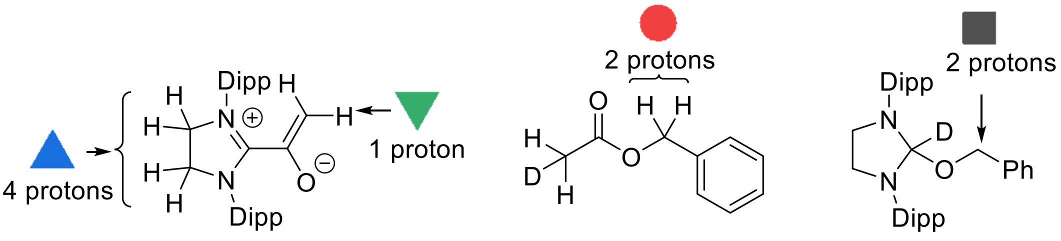

Figure S4. Protons integrated for monitoring the reaction kinetics and rate constant (k_D) values.

Table S2. Rate constant (k_D) values (two sets of data from two separate reactions) for each component (pseudo first order conditions).

Peak designation	δ [ppm]	Rate constant, k_D [1/min]	Error [1/min]
**5-*d*$_1$**, ■	6.63	1.05E-2	8E-6
**BnOAc-*d*$_1$**, ●	5.10	1.01E-2	8E-6
**1ae**, ▲	4.21	1.06E-2	3E-6
**1ae**, ▼	3.03	1.03E-2	5E-6
**5-*d*$_1$**, ■	6.64	1.04E-2	2E-5
**BnOAc-*d*$_1$**, ●	5.10	1.02E-2	1E-5
**1ae**, ▲	4.21	1.08E-2	4E-6
**1ae**, ▼	3.02	1.04E-2	6E-5

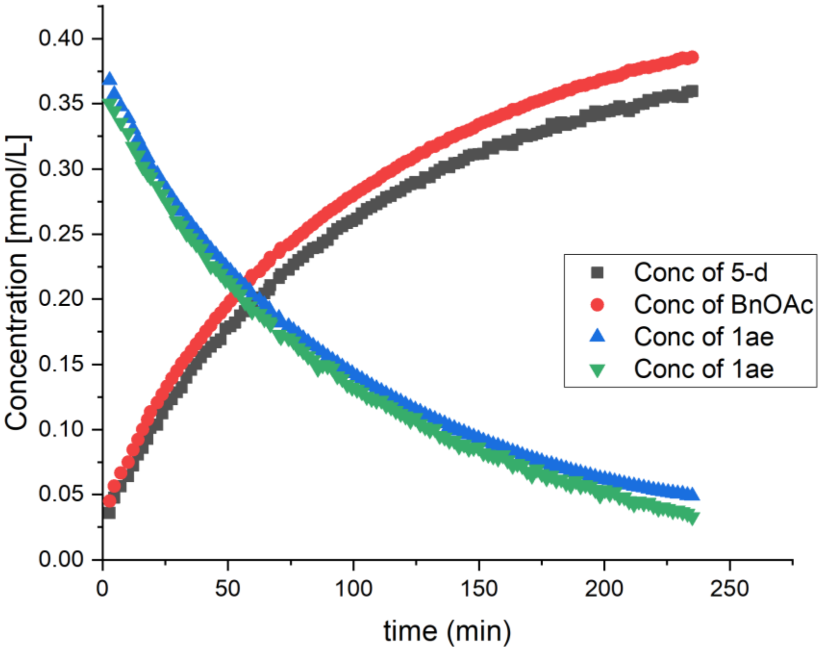

Figure S5. Concentration vs time profile of the ester formation from **1ae** in the presence of 8 equiv. of BnOD. Conditions are the same as in **Figure S3**.

Primary kinetic isotope effects were determined from the ratio of rate constant values (k_H from **Table S1,** entry **1ae**, ▲ and k_D from **Table S2,** entries **1ae**, ▲) for the ester formation from **1ae** in the presence of BnOH/BnOD.

$$KIE^1 = k_H/k_{D1} = 1.56E\text{-}2/1.06E\text{-}2 = 1.47$$
$$KIE^2 = k_H/k_{D2} = 1.56E\text{-}2/1.08E\text{-}2 = 1.44$$

7 NMR studies and reaction kinetics of ester formation from acyl azolium triflates in the presence of DBU

7.1 Reaction of the deuterated acyl azolium triflate 1aa-*d₃*•OTf with 1.5 equiv. of benzyl alcohol in the presence of DBU

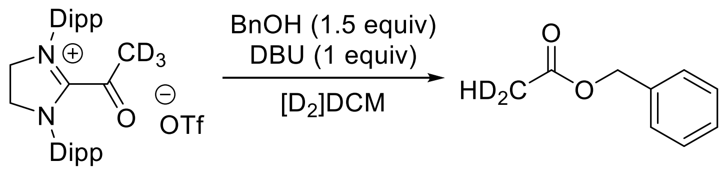

In a glovebox, the deuterated acyl azolium triflate **1aa-d_3•**OTf was prepared by reacting perdeuterated iso-propenyl acetate with SIPr followed by deuteration with deuterated triflic acid. The crude acyl azolium triflate **1aa-d_3•**OTf was then crystallized from a THF:DCM (1:1) mixture. An NMR tube was charged with 20 mg (34 µmol, 1.0 equiv.) of **1aa-d_3•**OTf in [D_2]DCM (0.5 mL), sealed with a septum, and 1.5 equiv. of benzyl alcohol (51 µmol, 5.3 µL) was added with a syringe. The reaction was followed by ^{1}H NMR spectroscopy. The formation of benzyl acetate was not observed. After addition of 1.0 equiv. of DBU (34 µmol, 5 µL), the formation of benzyl acetate-d_2 was observed. The kinetic experiment was conducted up to 50 min reaction time; however, the reaction took 6 h to reach complete conversion.

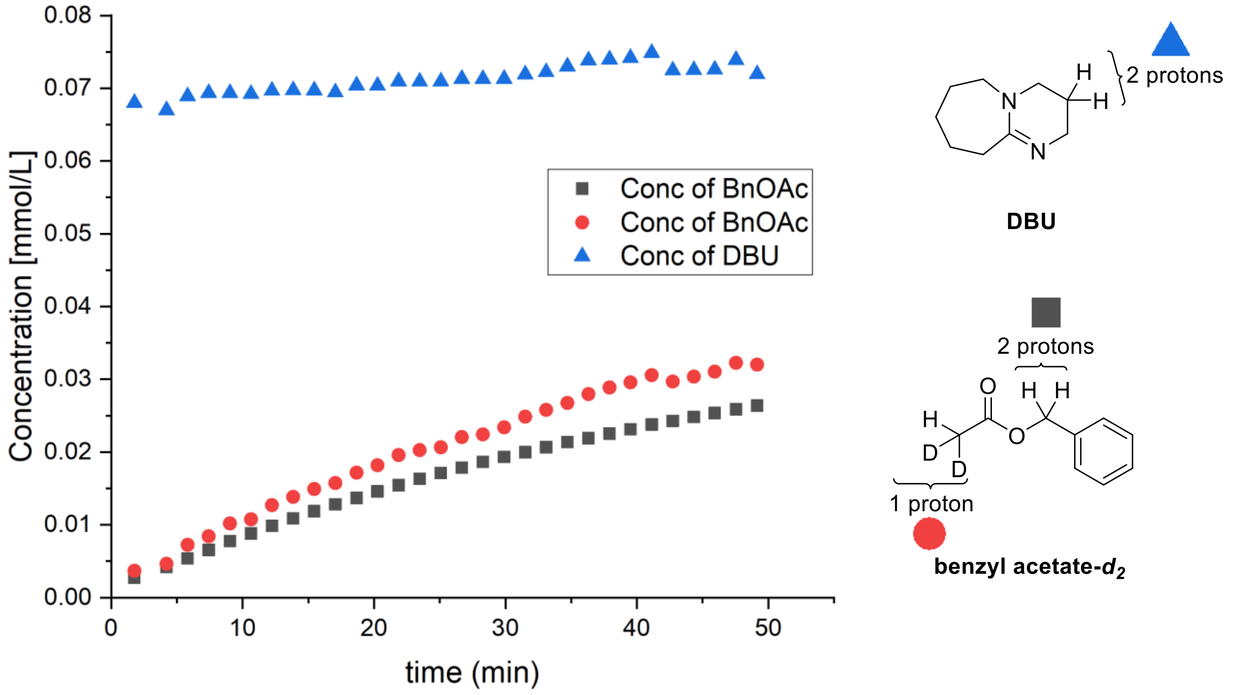

Figure S6. Reaction kinetics of the ester formation reaction from **1aa-d_3•**OTf in the presence of 1 equiv. of DBU.

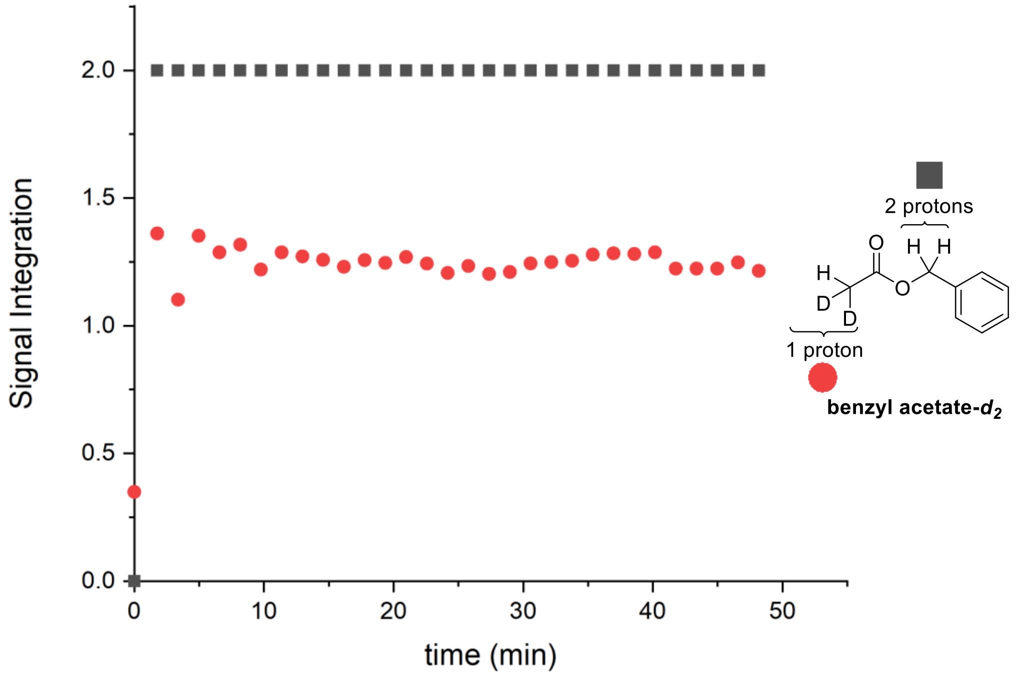

Figure S7. Time course of the integral of the **CD_2H** proton (red dots) in the course of the formation of benzyl acetate-*d*$_2$; integral of the two benzylic protons (black squares) arbitrarily set to two (no concomitant H/D-exchange was observed in the course of the reaction).

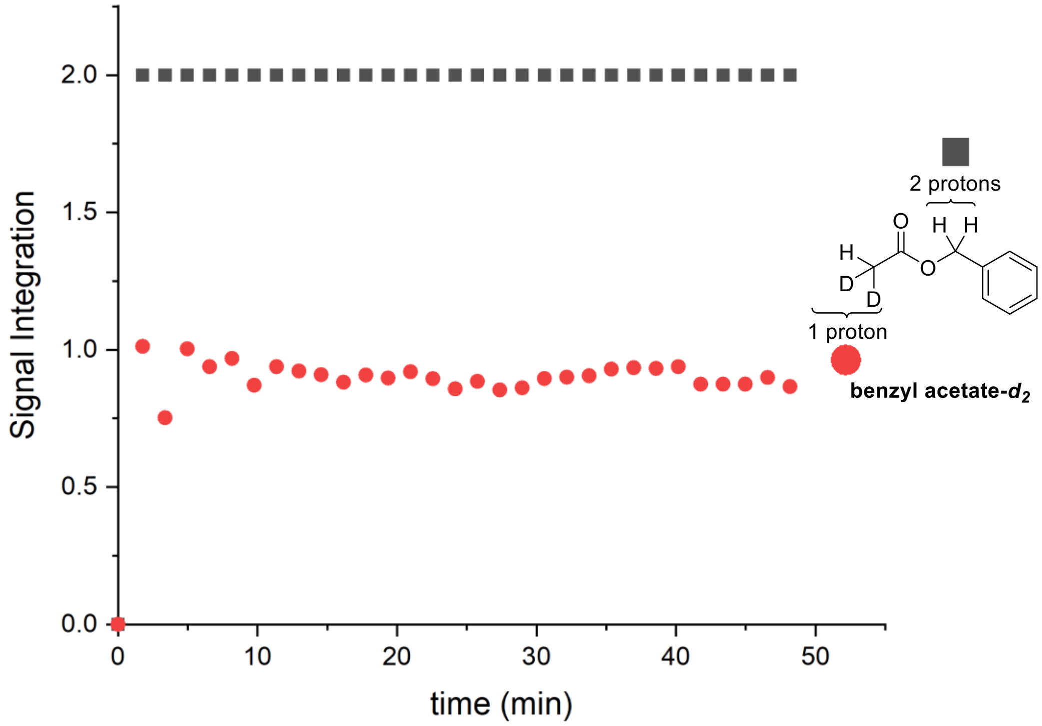

Figure S8. Same as **Figure S7**, except that the proton impurity of **CD_3** of the starting material **1aa-*d*$_3$**•OTf was subtracted (one proton introduced to benzyl acetate-*d*$_2$).

7.2 Generation of the azolium enolate 1ae from the acyl azolium triflate 1aa•OTf by treatment with DBU

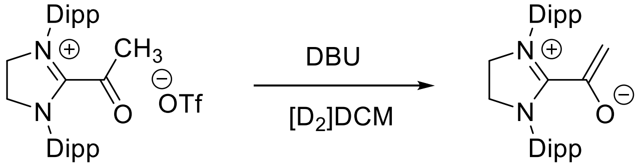

1aa•OTf **1ae**

An NMR tube was charged with 6 mg (10 µmol, 1.0 equiv.) of **1aa**•OTf in $[D_2]DCM$ (0.5 mL) and sealed with a septum. DBU (1 equiv, 1.5 µL) was added with a syringe and the reaction was followed by ^1H NMR spectroscopy. Clean formation of azolium enolate **1ae** was observed.

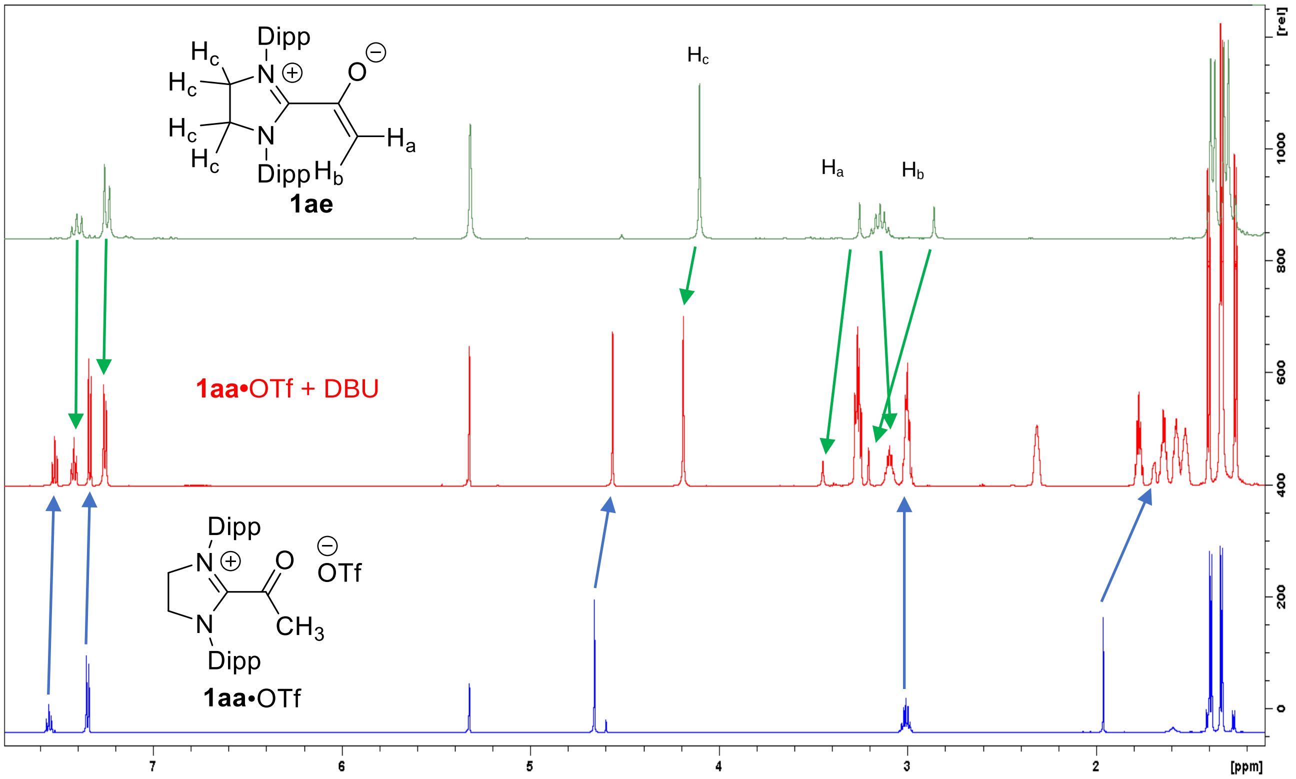

Figure S9. Top: ^1H NMR spectrum of **1ae** ($[D_2]DCM$, 300 MHz, 298 K). Middle: ^1H NMR spectrum of the reaction mixture immediately after addition of DBU ($[D_2]DCM$, 600 MHz, 298 K). Bottom: ^1H NMR spectrum of **1aa**•OTf ($[D_2]DCM$, 600 MHz, 298 K).

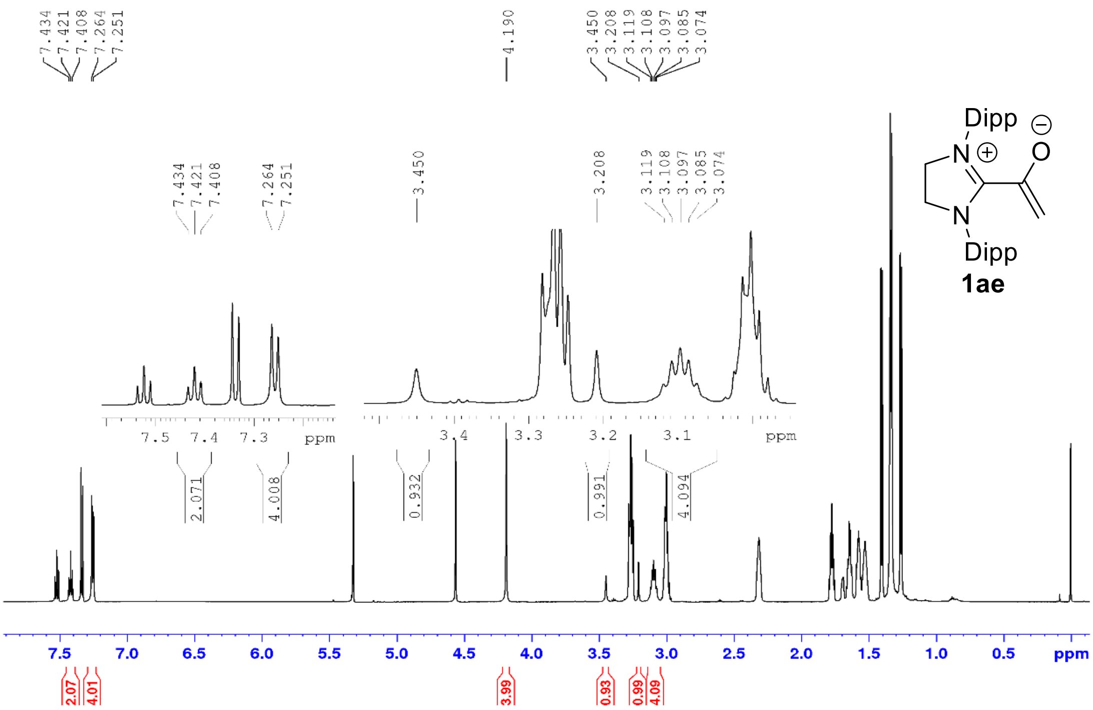

Figure S10. Same as **Figure S9**, middle, with integrals and insets of enlarged signals.

8 NMR studies on the ester/amide selectivity of azolium enolate 1ae and acyl azolium triflate 1aa.OTf

8.1 Reaction of the azolium enolate 1ae with (1:1) equiv. of benzyl alcohol and benzylamine

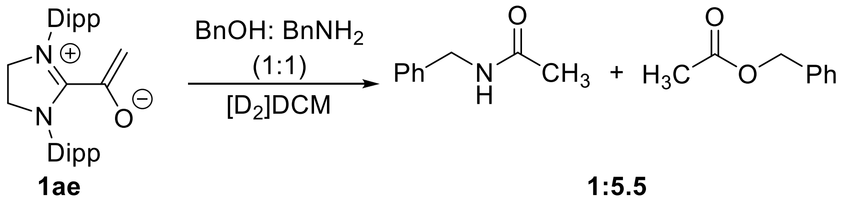

In a glovebox, the azolium enolate **1ae** was generated and crystalized as described before. An NMR tube was charged with 8 mg (18 µmol, 1.0 equiv) of **1ae** in [D$_2$]DCM (0.5 mL) and sealed with a septum. Benzyl alcohol (1 equiv, 1.9 µl) and benzylamine (1 equiv, 2.02 µl) were added with a syringe and the reaction was followed by ^{1}H NMR spectroscopy. The formation of benzyl acetate and N-benzylacetamide was observed in a 5.5:1 ratio after 18 h at RT. For comparison, benzyl acetate[2] and N-benzylacetamide[1] were prepared according to literature procedures.

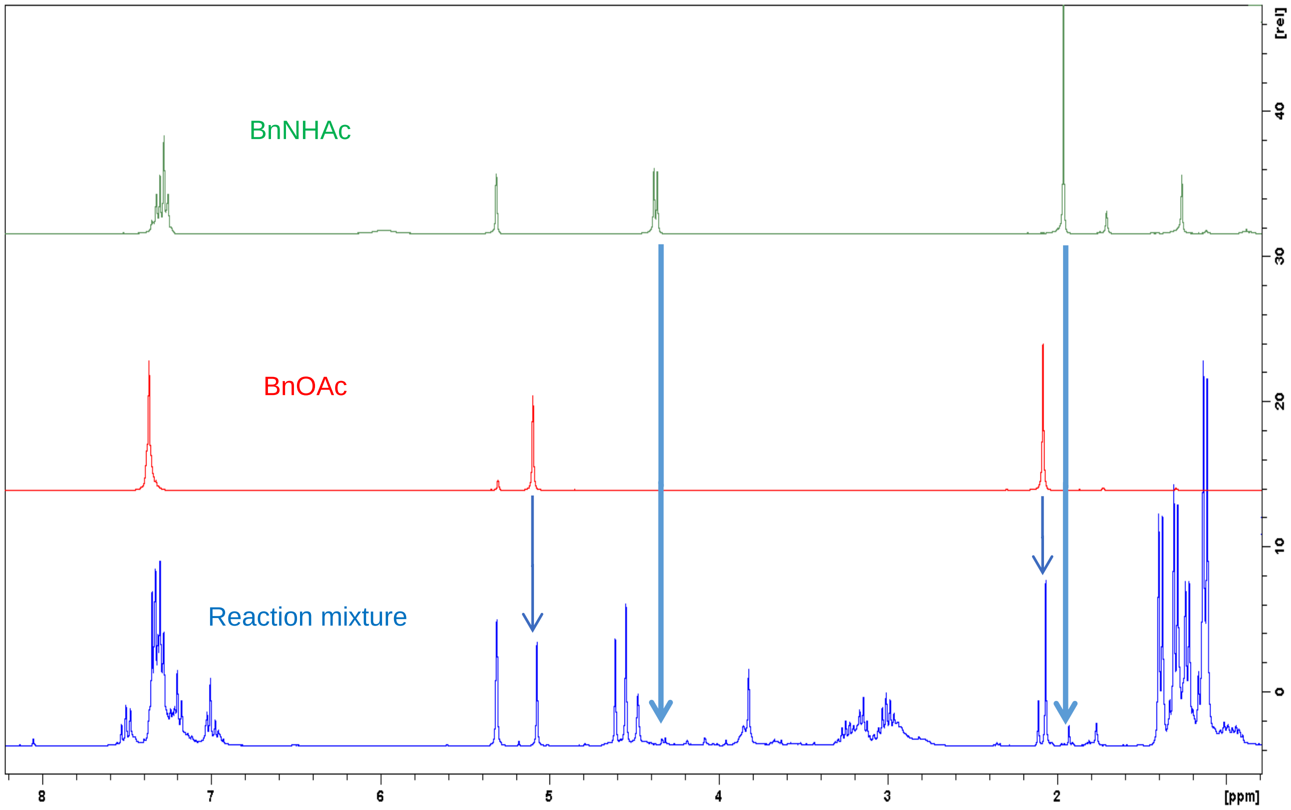

Figure S11. Top: ^{1}H NMR spectrum of N-benzylacetamide ([D2]DCM, 300 MHz, 298 K). Middle: ^{1}H NMR spectrum of benzyl acetate ([D2]DCM, 300 MHz, 298 K). Bottom: ^{1}H NMR spectrum (recorded after 18 h in [D2]DCM, 300 MHz, 298 K) resulting from the reaction of the azolium enolate **1ae** with 1:1 BnOH and $BnNH_2$.

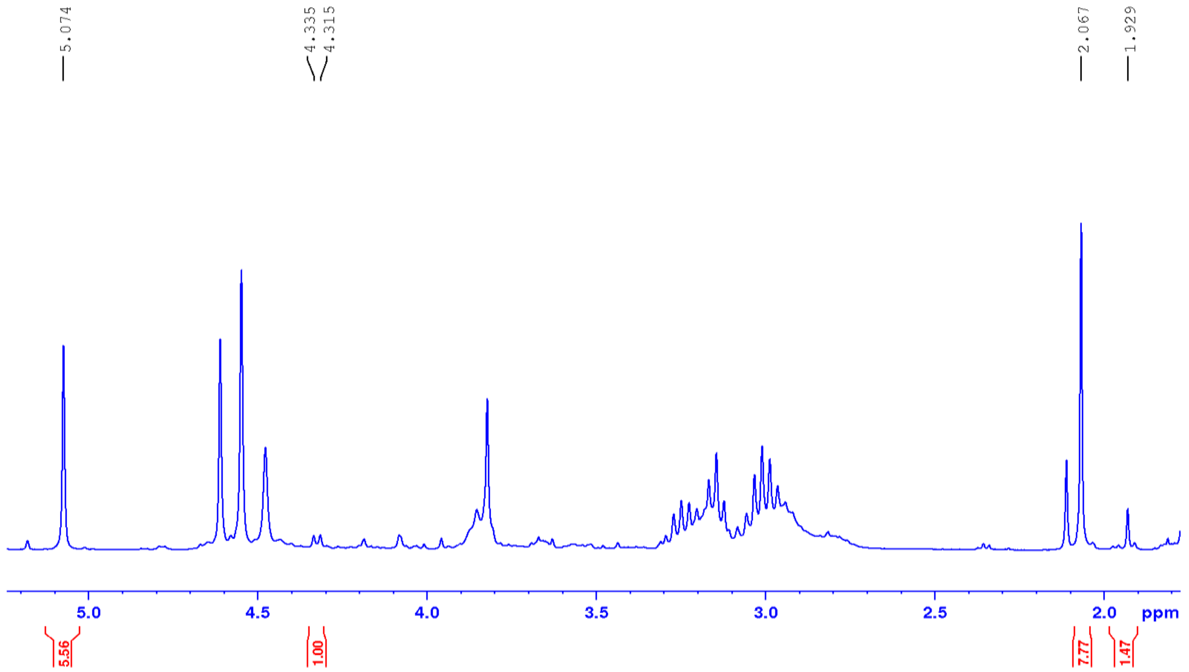

Figure S12. Same as **Figure S11**, bottom, enlarged and with integrals.

8.2 Reaction of the acyl azolium triflate 1aa•OTf with (1:1) equiv. of benzyl alcohol and benzylamine

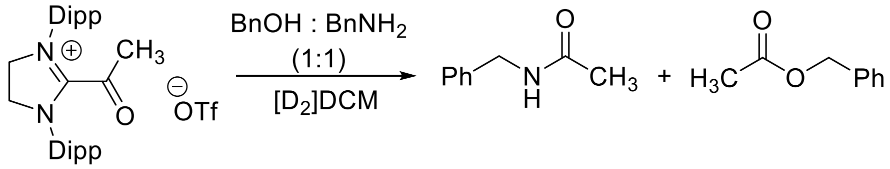

1aa•OTf

8.9:1

In a glovebox, the acyl azolium triflate **1aa**•OTf was generated and crystalized as described before. An NMR tube was charged with 20 mg (34 µmol, 1.0 equiv) of **1aa**•OTf in [D$_2$]DCM (0.5 mL) and sealed with a septum. Benzyl alcohol (1 equiv, 3.5 µl) and benzylamine (1 equiv, 3.7 µl) were added with a syringe and the reaction was followed by ^{1}H NMR spectroscopy. The formation of benzyl acetate and N-benzylacetamide was observed in a 1:8 ratio after 18 h at RT.

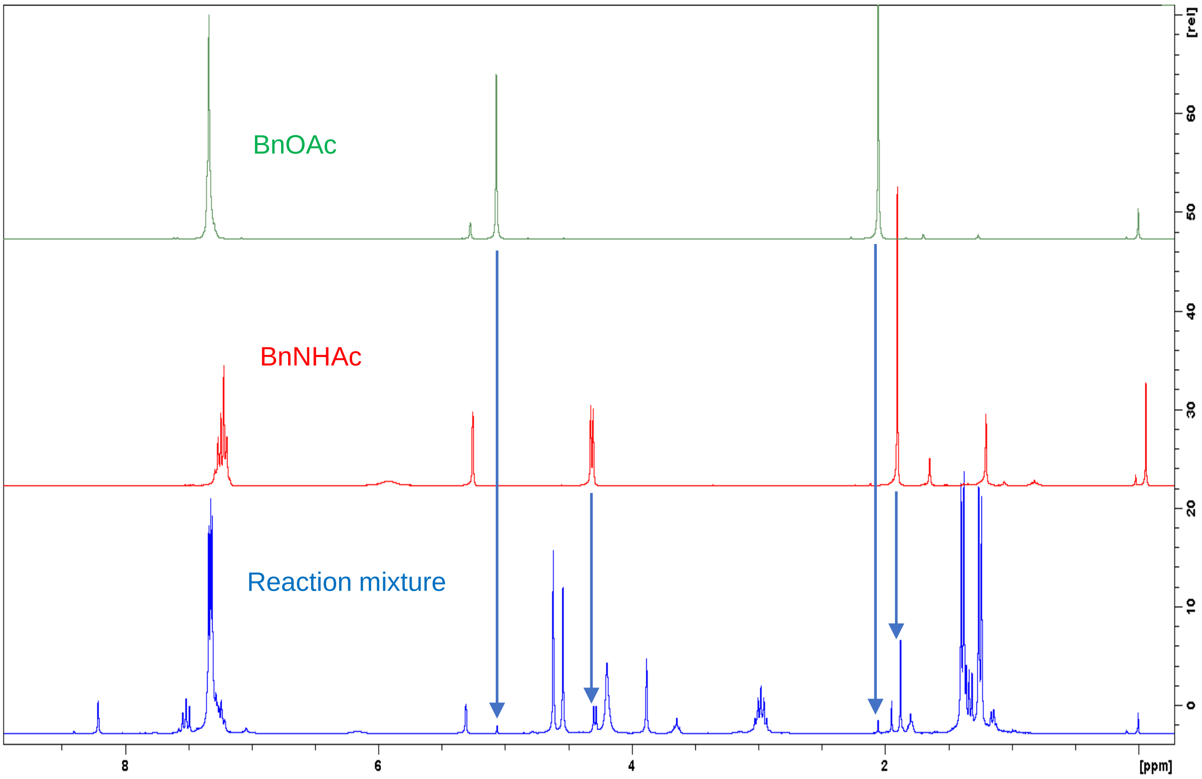

Figure S13. Top: ^{1}H NMR spectrum of benzyl acetate ([D$_2$]DCM, 300 MHz, 298 K). Middle: ^{1}H NMR spectrum of N-benzylacetamide ([D$_2$]DCM, 300 MHz, 298 K). Bottom: ^{1}H NMR spectrum (recorded after 18 h in [D$_2$]DCM, 300 MHz, 298 K) resulting from the reaction of the acyl azolium triflate **1aa**•OTf with 1:1 BnOH and BnNH$_2$.

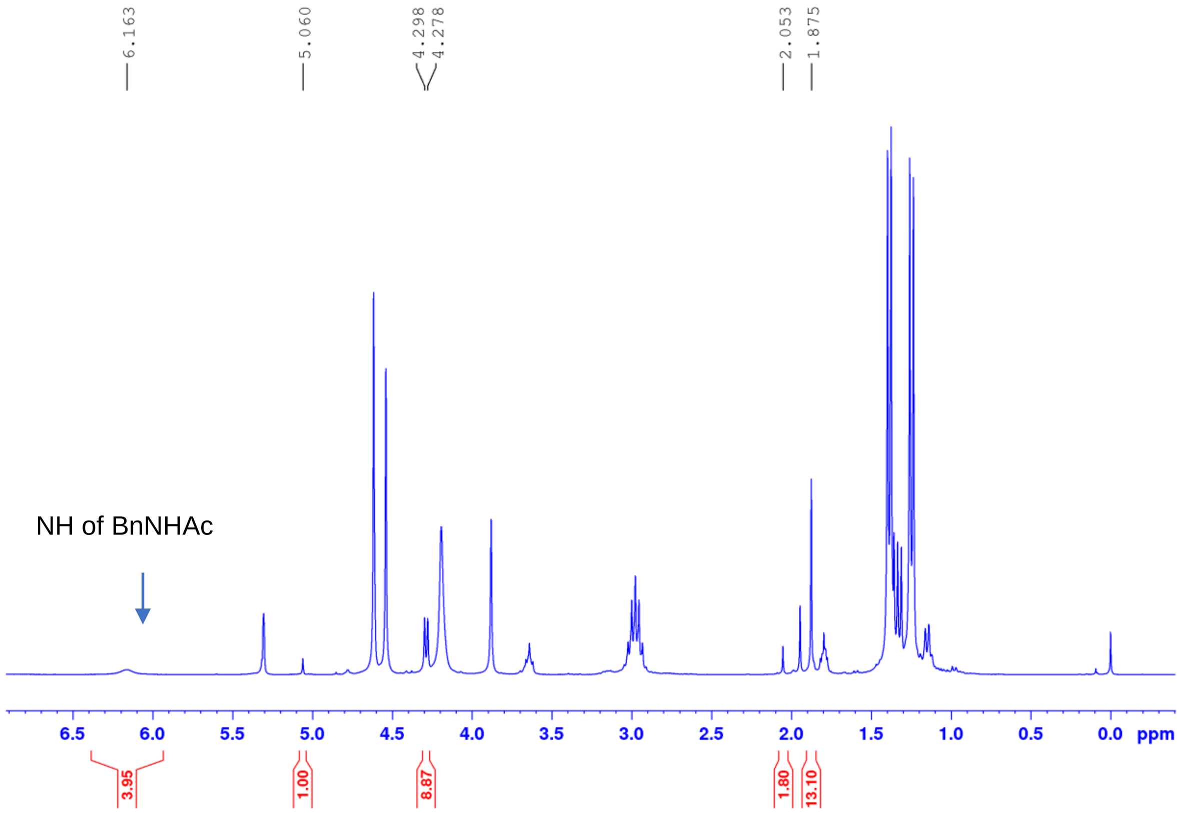

Figure S14. Same as **Figure S13**, bottom, enlarged and with integrals.

9 Control experiment for secondary ester-to-amide transformation

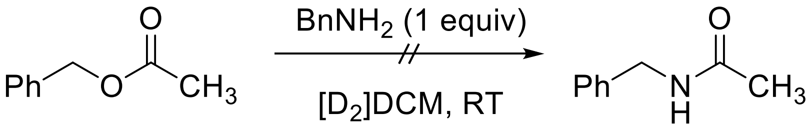

An NMR tube was charged with 8.7 mg (58 µmol, 1.0 equiv.) of benzyl acetate in [D$_2$]DCM (0.5 mL), and sealed with a septum. Benzylamine (1 equiv, 6.3 µL) was added with a syringe and the reaction was followed by ^{1}H NMR spectroscopy. No reaction was observed in an overnight measurement at RT. For comparison, N-benzylacetamide was prepared according to a literature procedure.[1]

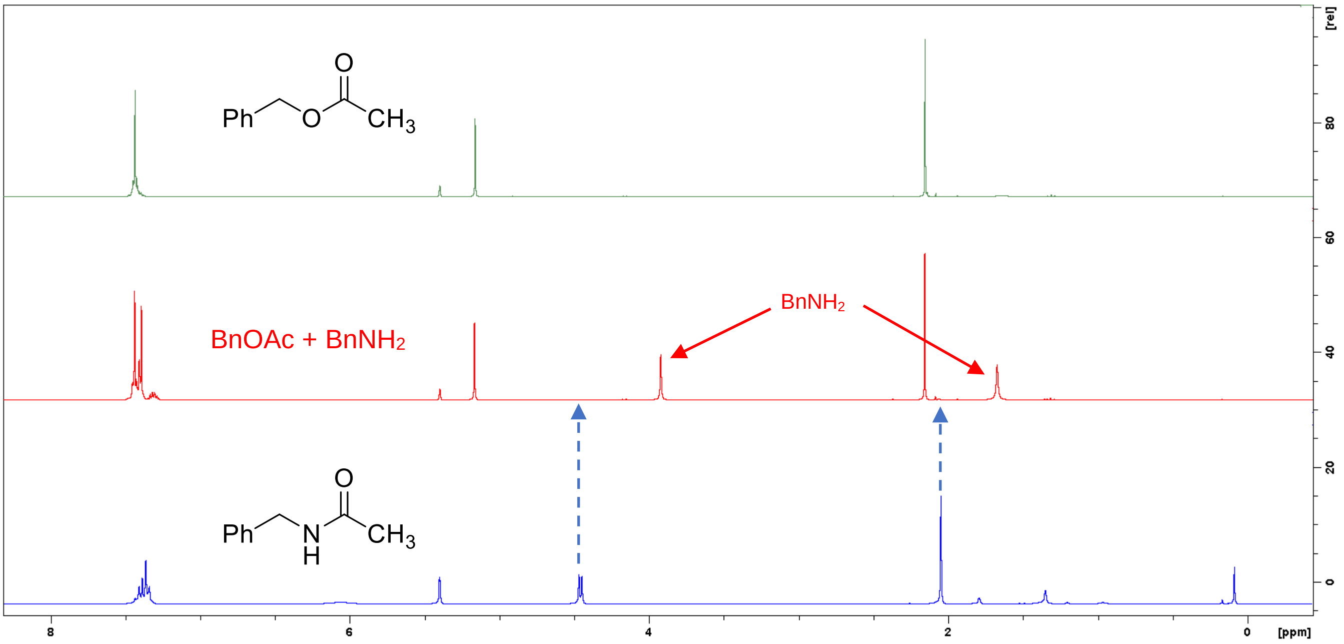

Figure S15. Top: ^{1}H NMR spectrum of benzyl acetate ([D$_2$]DCM, 300 MHz, 298 K). Middle: ^{1}H NMR spectrum recorded 14 h after addition of benzylamine to benzyl acetate ([D$_2$]DCM, 300 MHz, 298 K). Bottom: ^{1}H NMR spectrum of N-benzylacetamide ([D$_2$]DCM, 300 MHz, 298 K).

10 NMR Spectra

10.1 1D and 2D NMR spectra of 1-(1,3-bis(2,6-diisopropylphenyl)imidazolidin-2-yl)propan-2-one 4

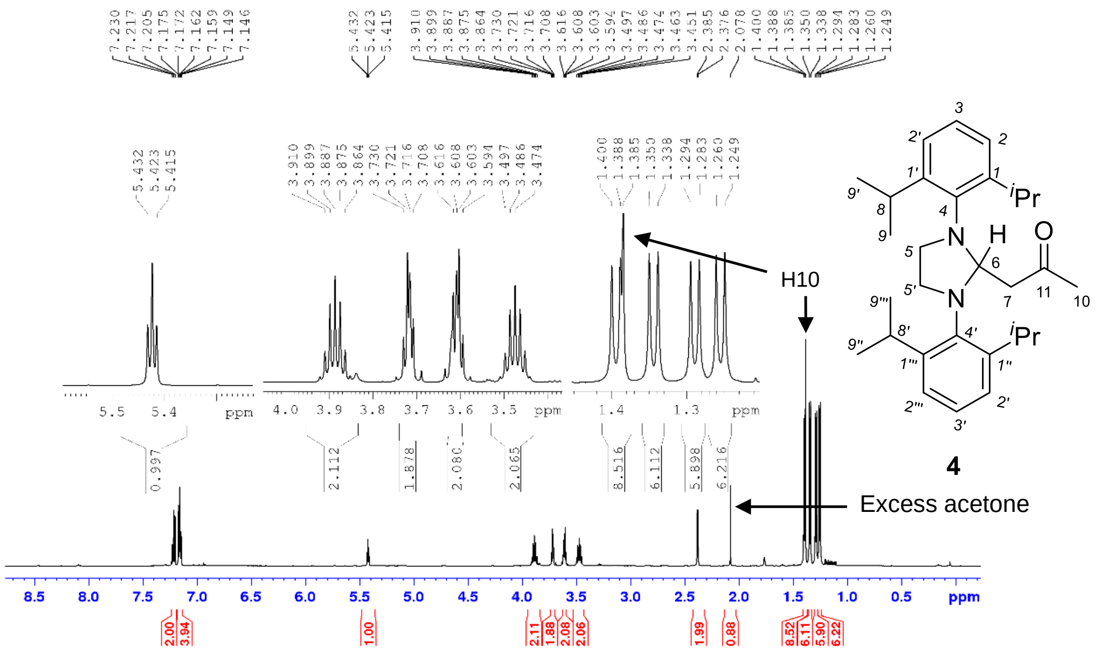

Figure S16. ^{1}H(600 MHz) NMR spectrum of **4** ([D$_8$]THF, 298 K).

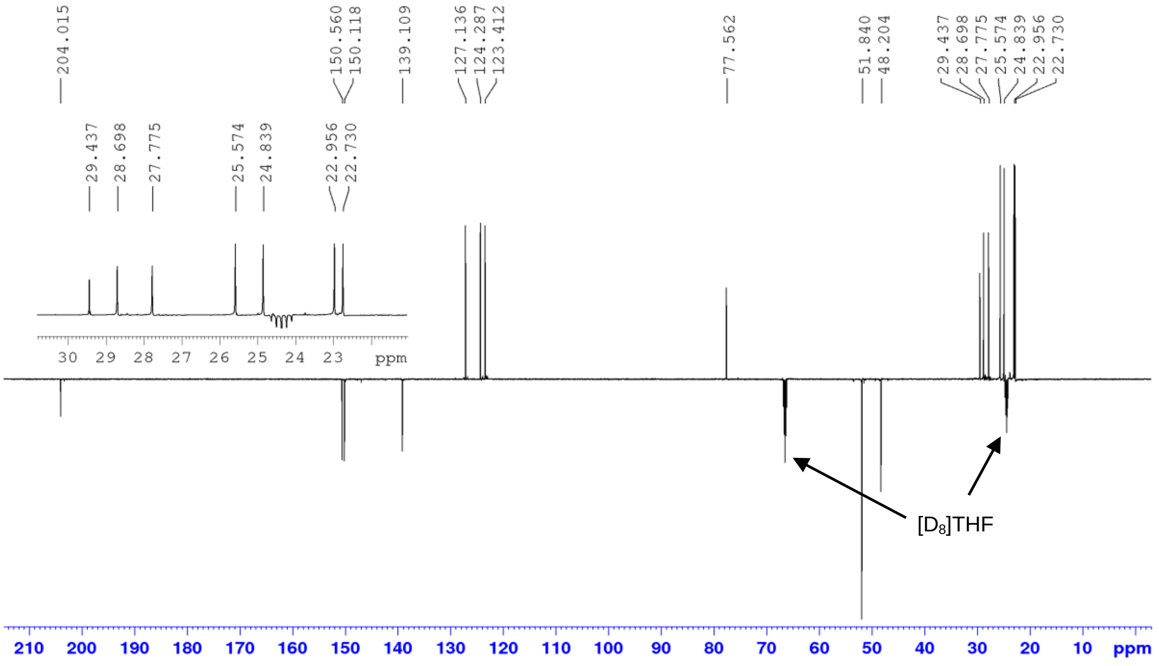

Figure S17. ^{13}C(150 MHz) DEPTQ NMR spectrum of **4** ([D$_8$]THF, 298 K).

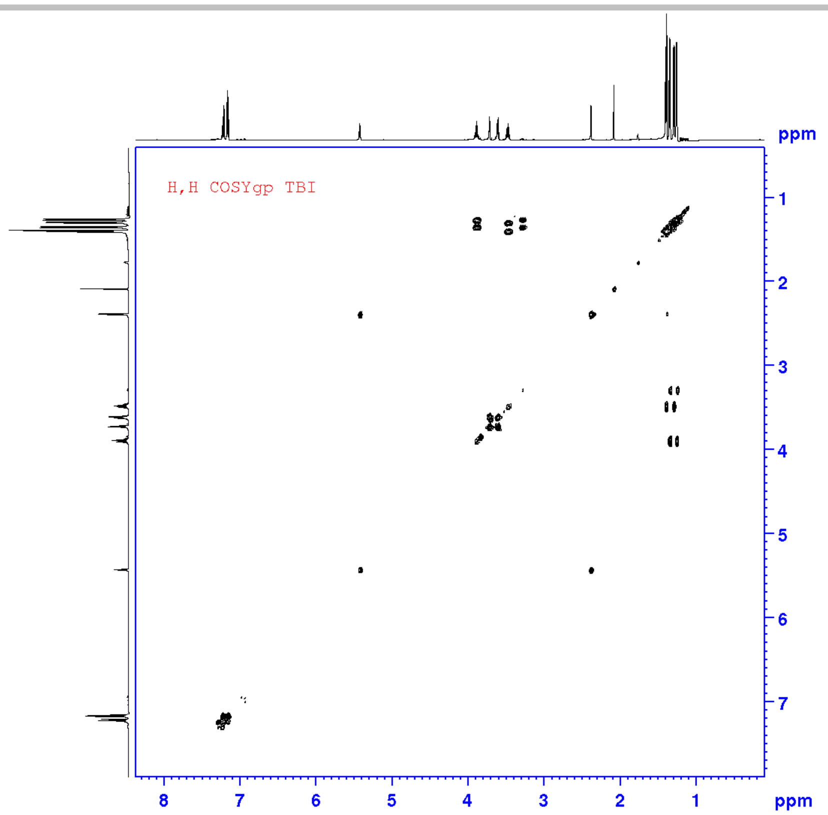

Figure S18. ^{1}H, ^{1}H(600MHz) COSY NMR spectrum of **4** ([D8]THF, 298 K).

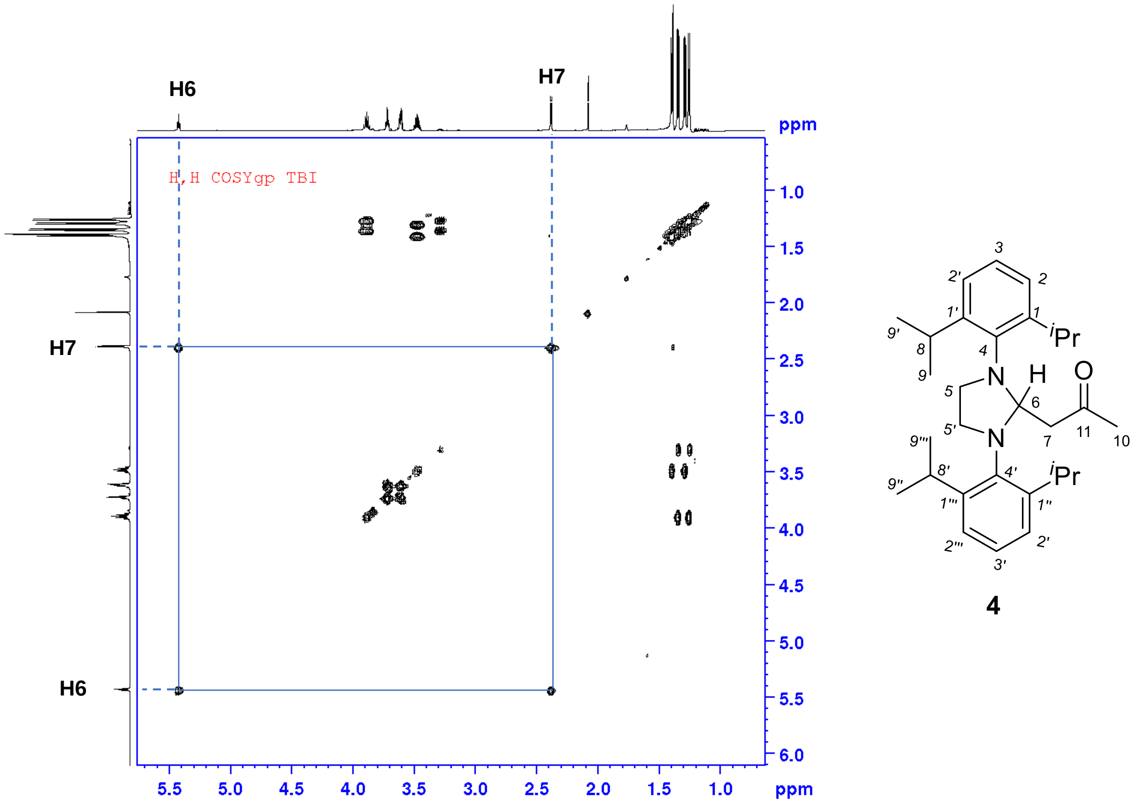

Figure S19. Part of ^{1}H, ^{1}H(600MHz) COSY NMR spectrum of **4** ([D8]THF, 298 K).

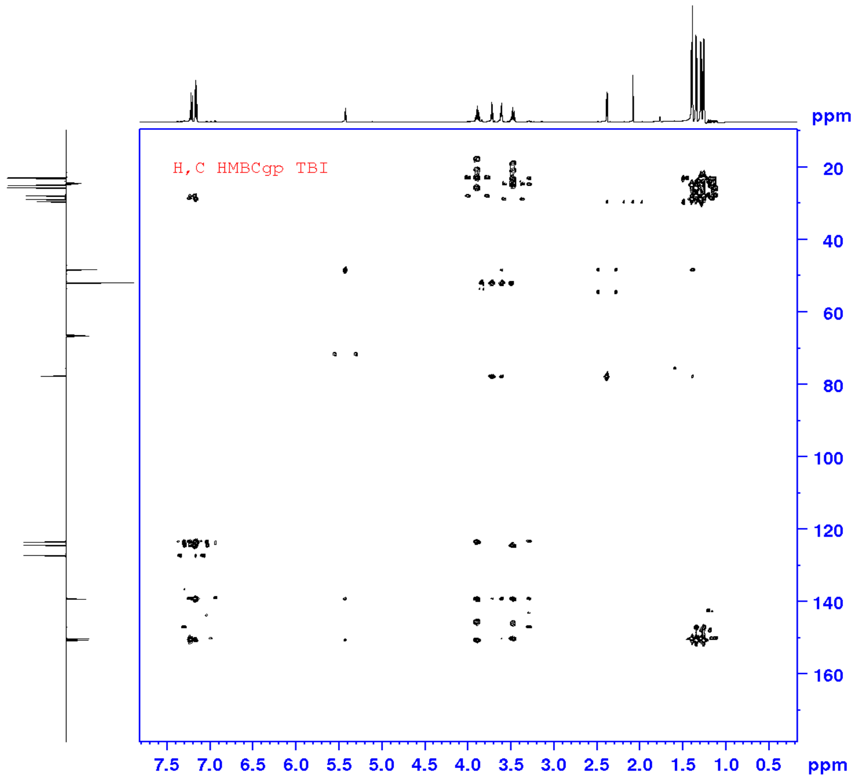

Figure S20. ^{1}H(600 MHz),^{13}C(150 MHz) HMBC NMR spectrum of **4** ([D$_8$]THF, 298 K).

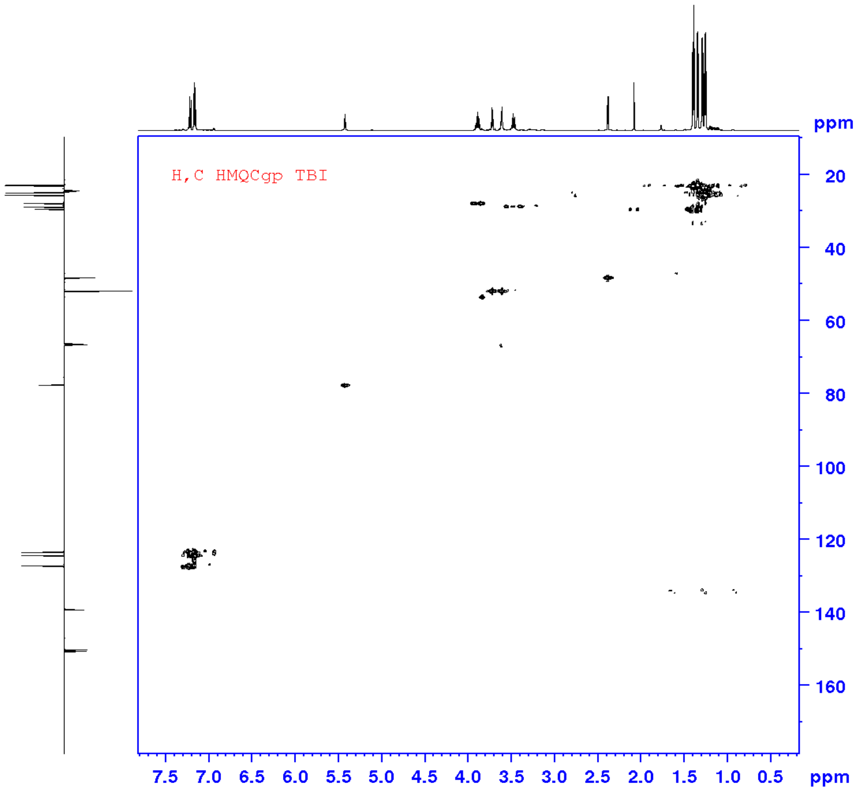

Figure S21. ^{1}H(600 MHz),^{13}C(150 MHz) HSQC NMR spectrum of **4** ([D$_8$]THF, 298 K).

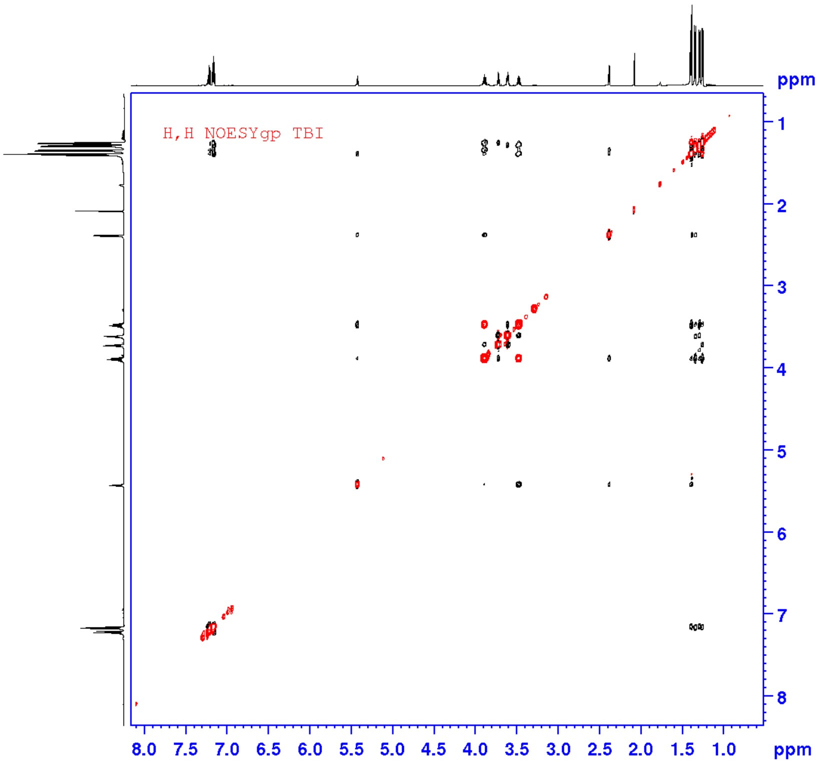

Figure S22. ^{1}H, ^{1}H(600MHz) NOESY NMR spectrum of **4** ([D8]THF, 298 K).

10.2 1D and 2D NMR spectra of 1-{1,3-bis[2,6-di(propan-2-yl)phenyl]-4,5-dihydro-1*H*-imidazol-3-ium-2-yl}-ethenolate 1ae

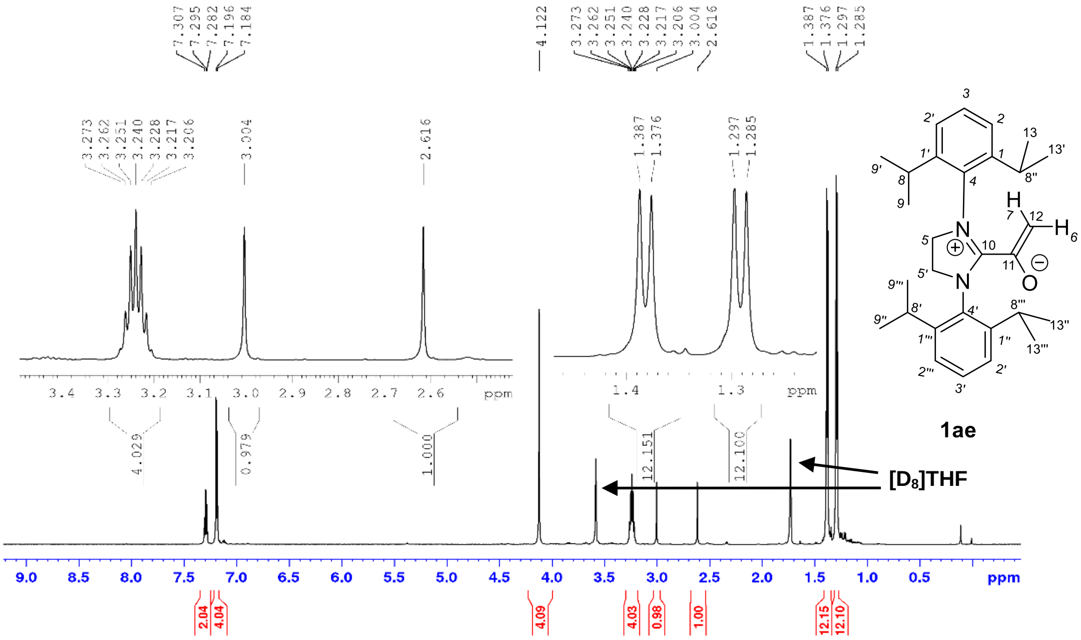

Figure S23. ^{1}H(600 MHz) NMR spectrum of **1ae** ([D8]THF, 298 K).

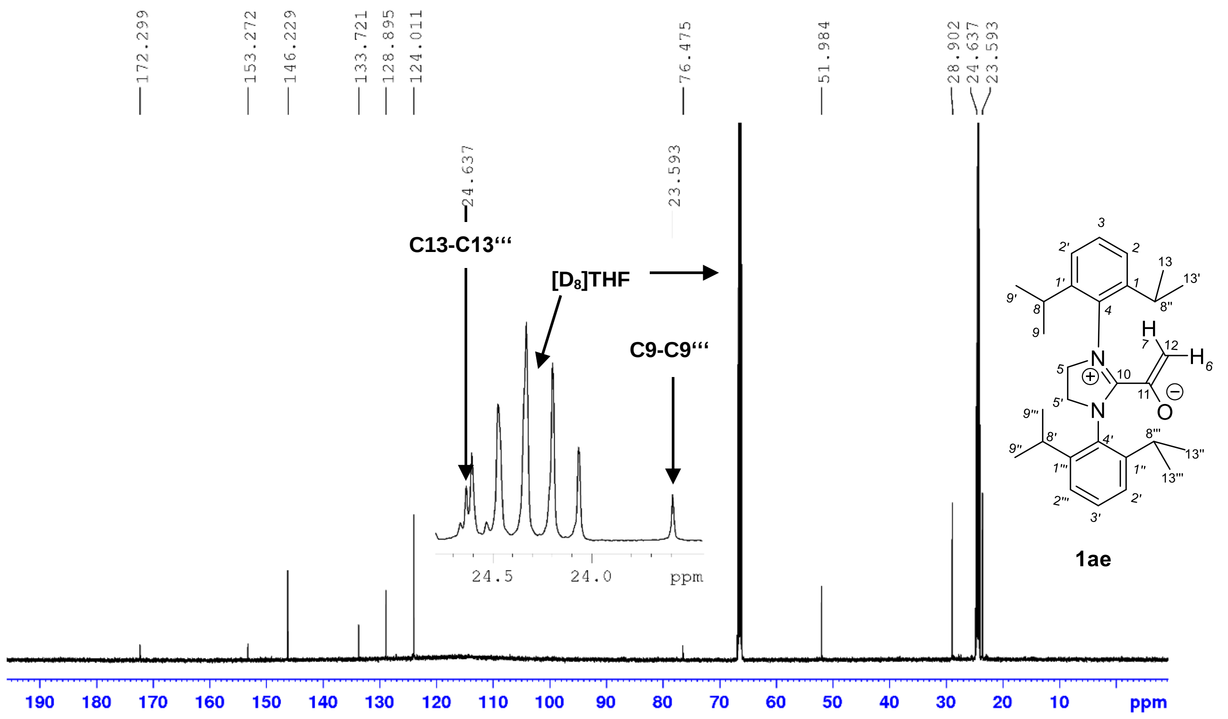

Figure S24. ^{13}C(150 MHz) NMR spectrum of **1ae** ([D$_8$]THF, 298 K).

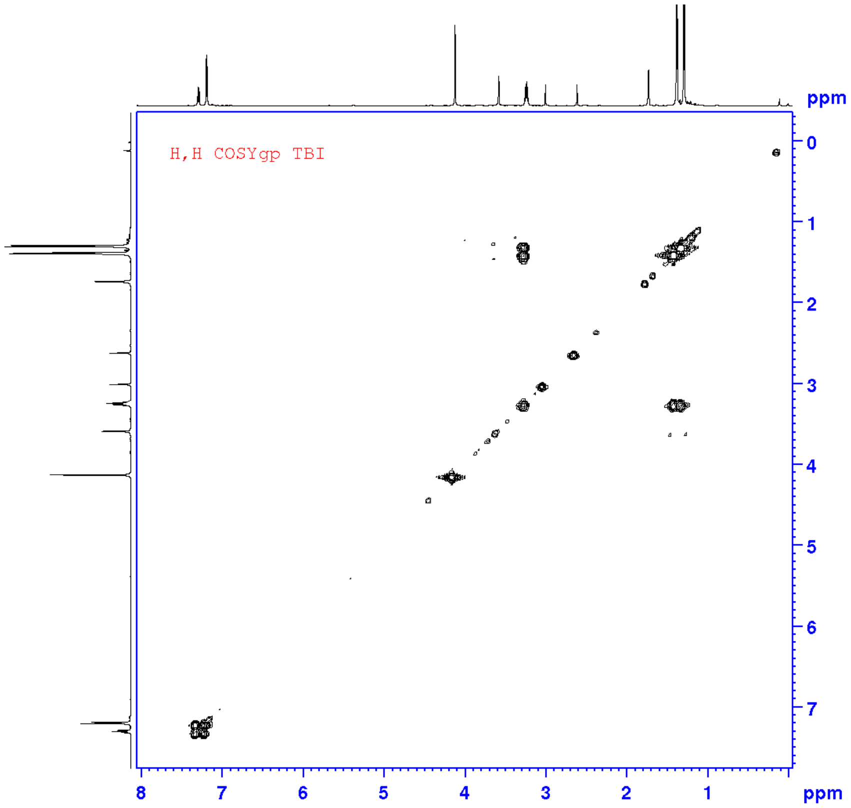

Figure S25. ^{1}H, ^{1}H(600MHz) COSY NMR spectrum of **1ae** ([D$_8$]THF, 298 K).

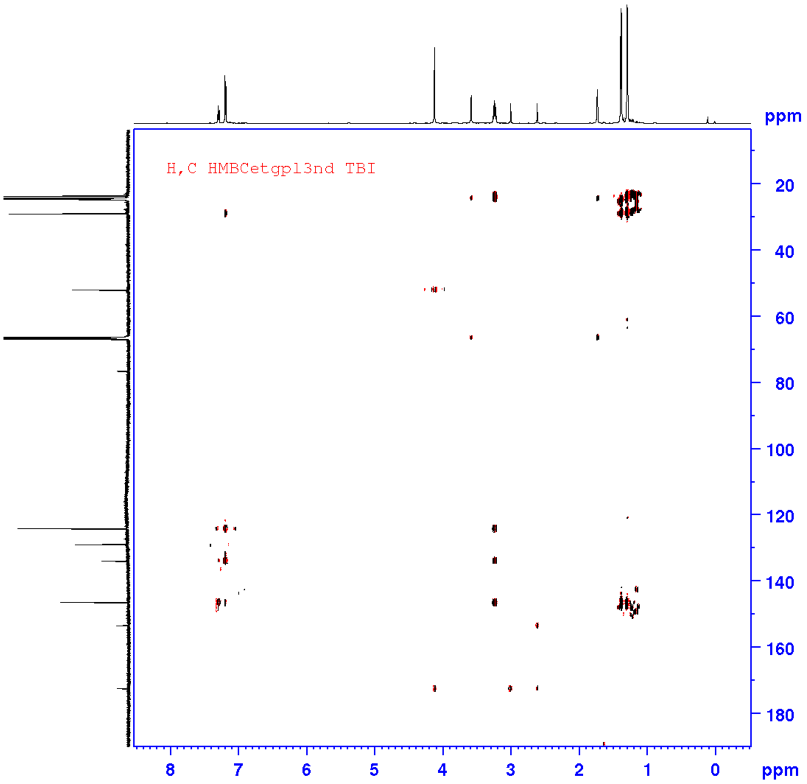

Figure S26. ^{1}H(600 MHz),^{13}C(150 MHz) HMBC NMR spectrum of **1ae** ([D8]THF, 298 K).

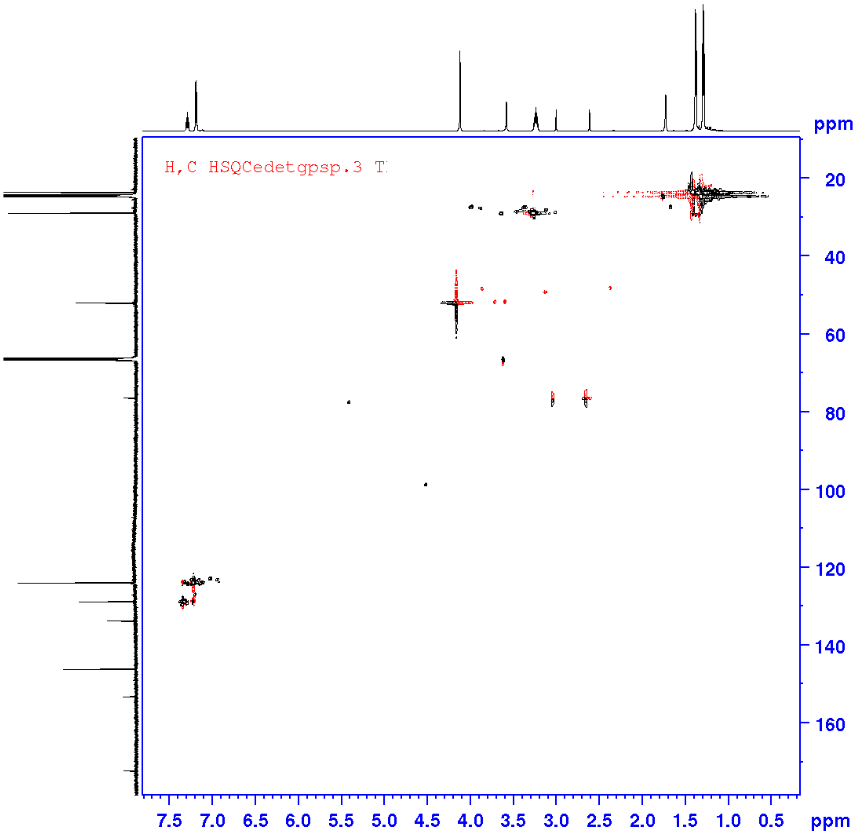

Figure S27. ^{1}H(600 MHz),^{13}C(150 MHz) HSQC NMR spectrum of **1ae** ([D8]THF, 298 K).

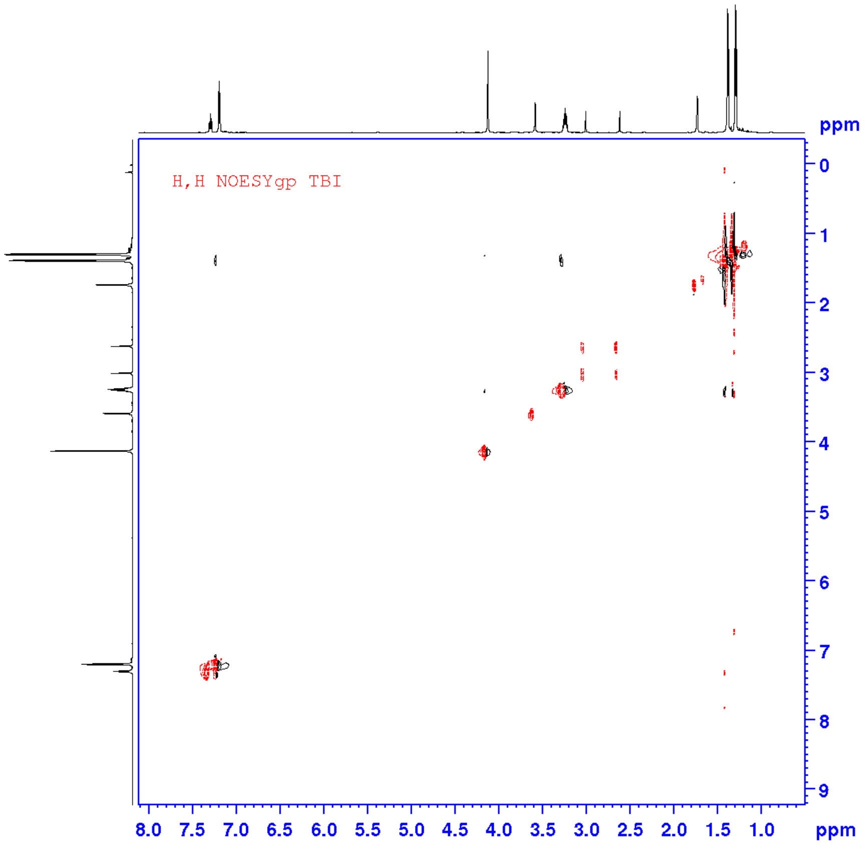

Figure S28. ^{1}H, ^{1}H(600MHz) NOESY NMR spectrum of **1ae** ([D8]THF, 298 K).

10.3 1D and 2D NMR spectra of {1,3-bis[2,6-di(propan-2-yl)phenyl]-1*H*-imidazol-3-ium-2-yl}ethenolate 2ae

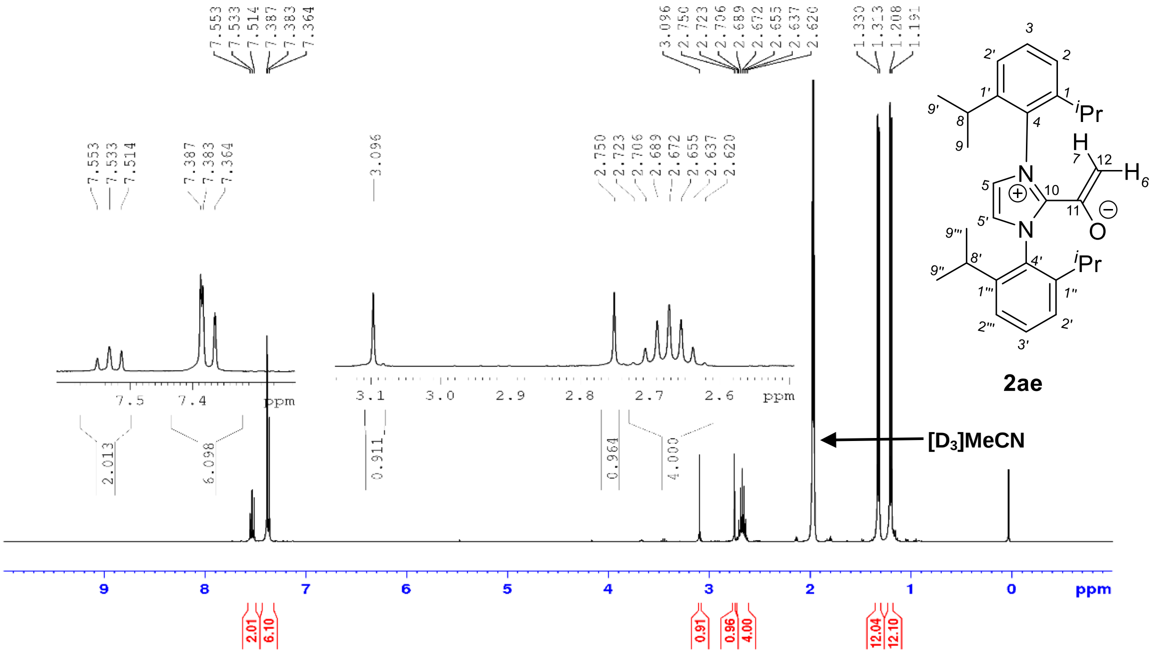

Figure S29. ^{1}H(400 MHz) NMR spectrum of **2ae** ([D3]MeCN, 298 K).

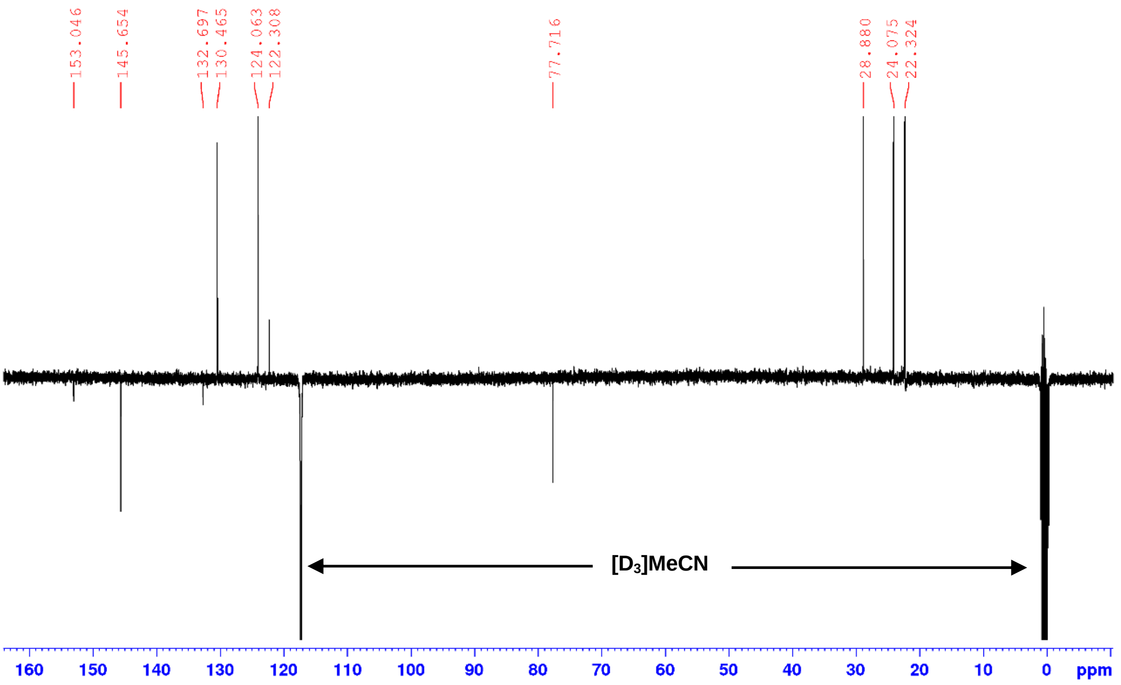

Figure S30. ^{13}C(100 MHz) DEPTQ NMR spectrum of **2ae** ([D3]MeCN, 298 K).

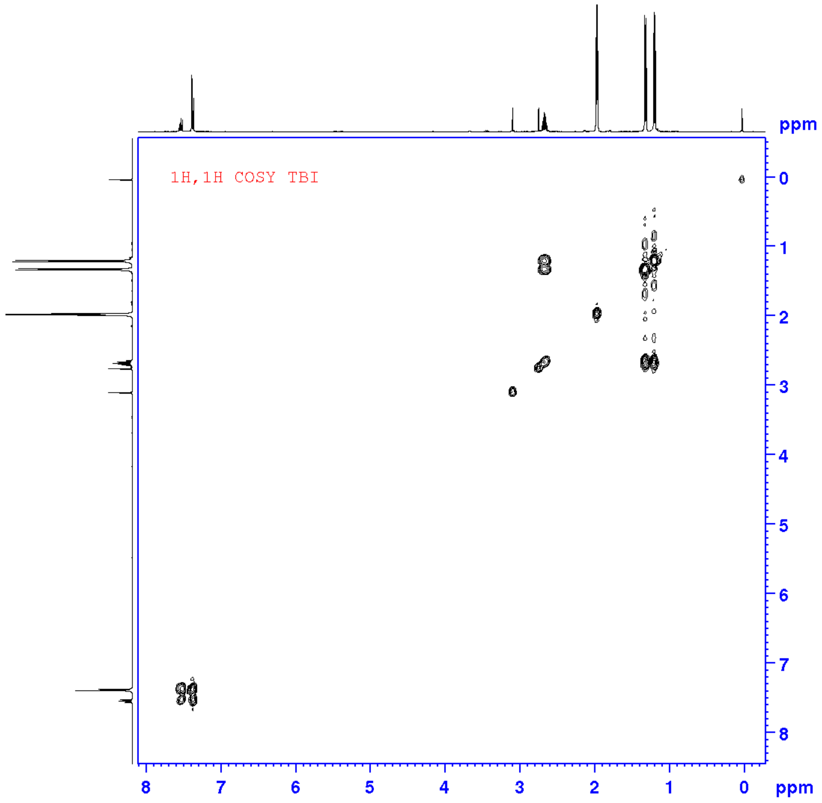

Figure S31. ^{1}H, ^{1}H(400MHz) COSY NMR spectrum of **2ae** ([D3]MeCN, 298 K).

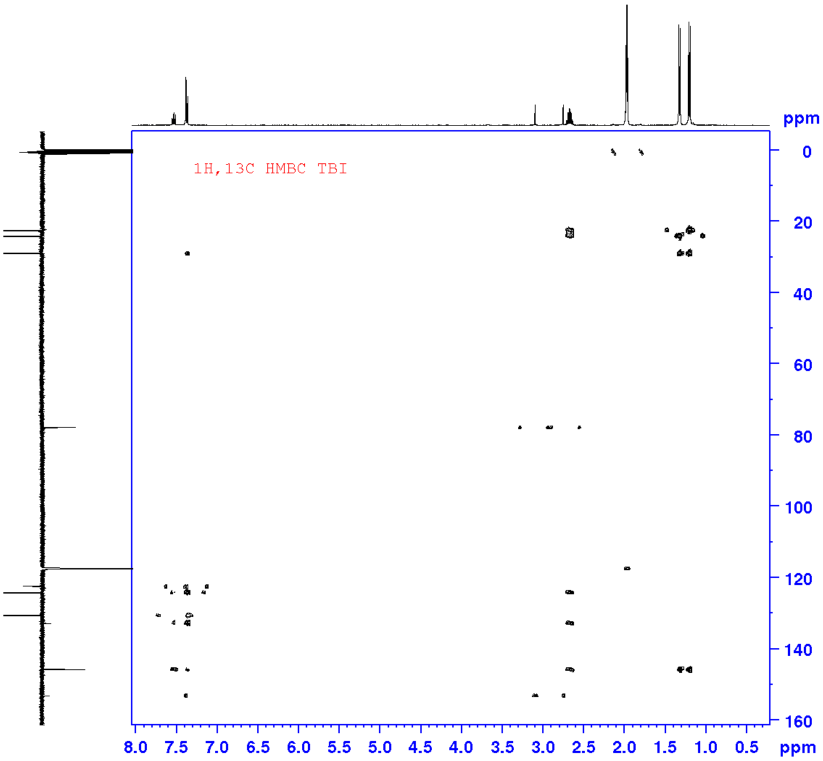

Figure S32. ^{1}H(400 MHz),^{13}C(100 MHz) HMBC NMR spectrum of **2ae** ([D3]MeCN, 298 K).

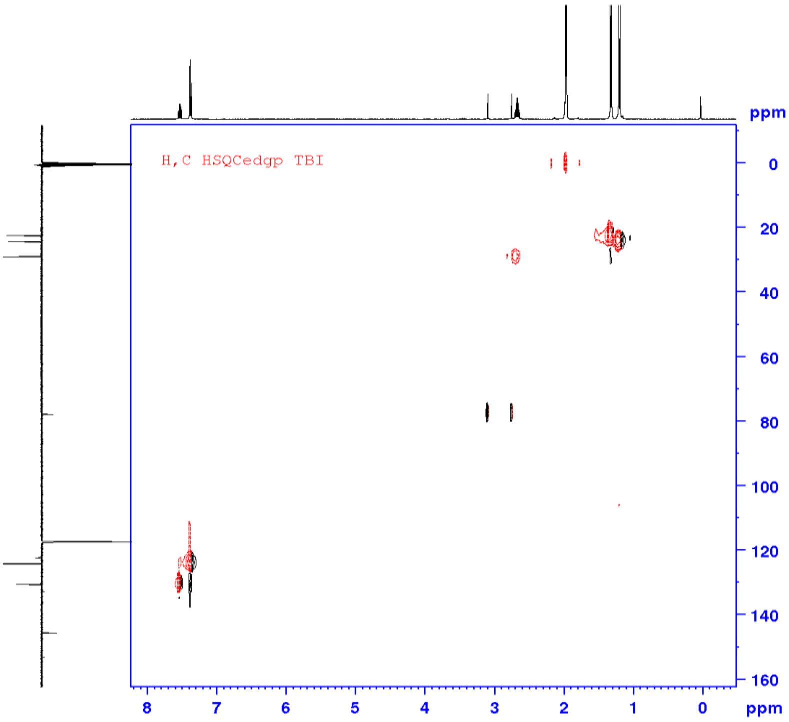

Figure S33. ^{1}H(400 MHz),^{13}C(100 MHz) HSQC NMR spectrum of **2ae** ([D3]MeCN, 298 K).

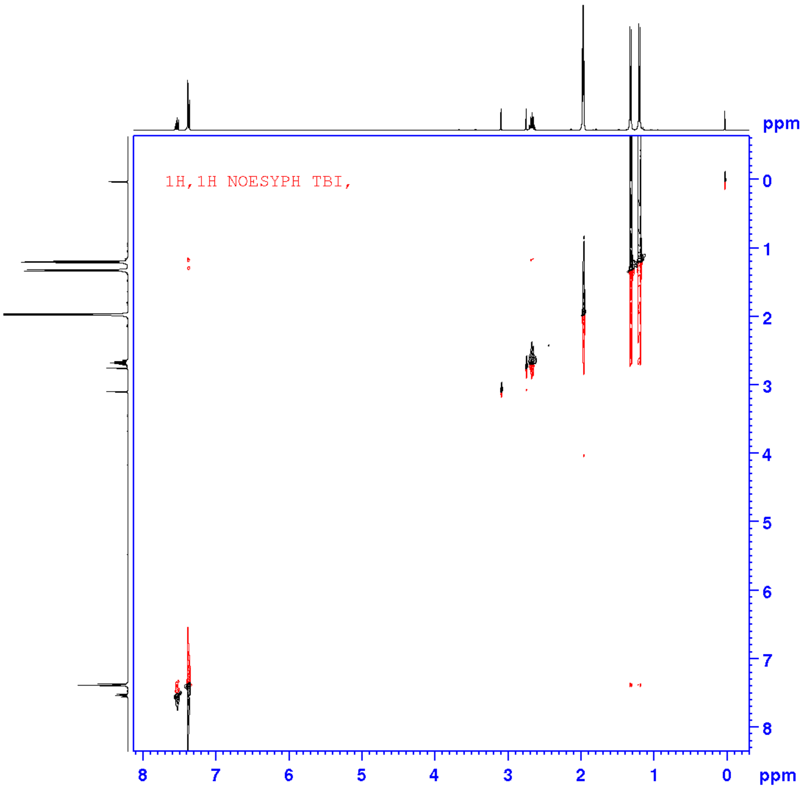

Figure S34. ^{1}H, ^{1}H(400MHz) NOESY NMR spectrum of **2ae** ([D$_3$]MeCN, 298 K).

10.4 1D and 2D NMR spectra of {1,3-bis(2,4,6-trimethylphenyl)-1*H*-imidazol-3-ium-2-yl}ethenolate 3ae

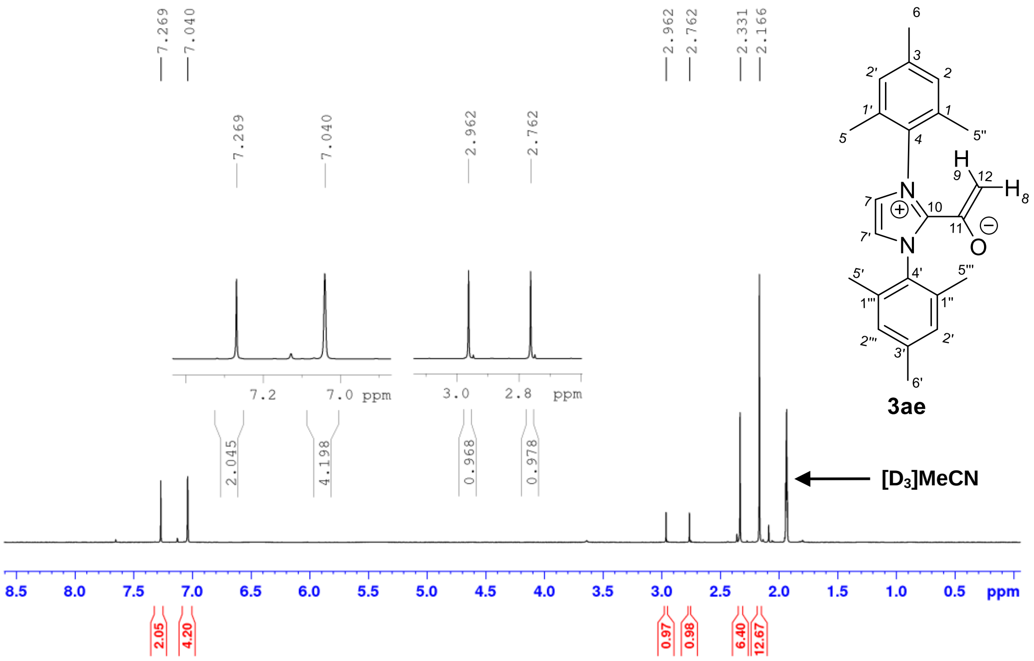

Figure S35. ^{1}H(600 MHz) NMR spectrum of **3ae** ([D$_3$]MeCN, 298 K).

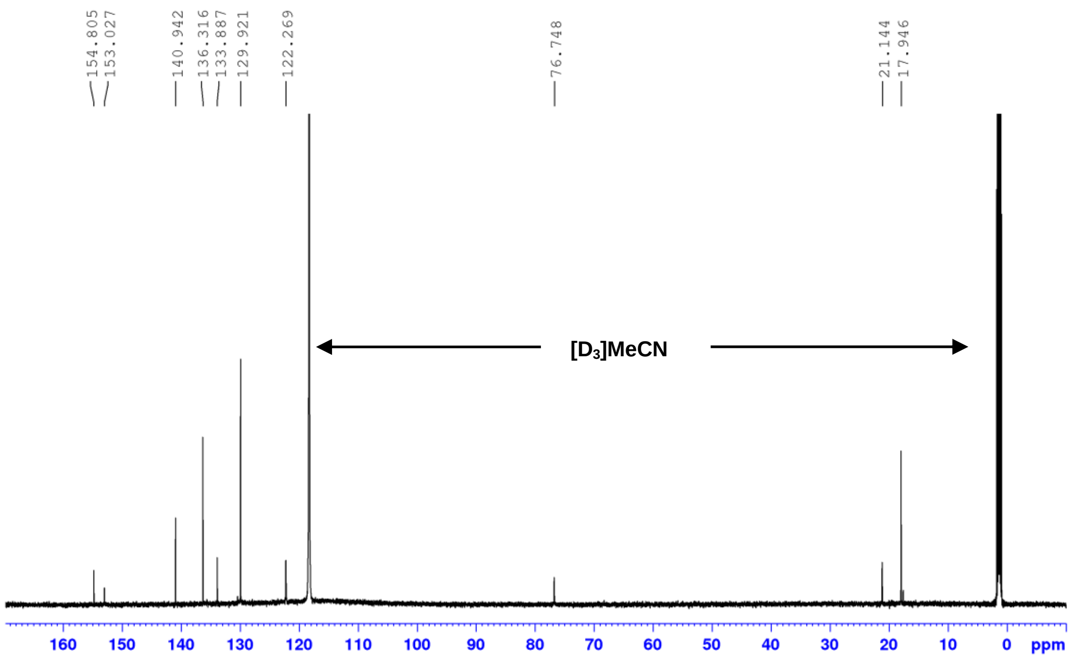

Figure S36. ^{13}C(150 MHz) NMR spectrum of **3ae** ([D3]MeCN, 298 K).

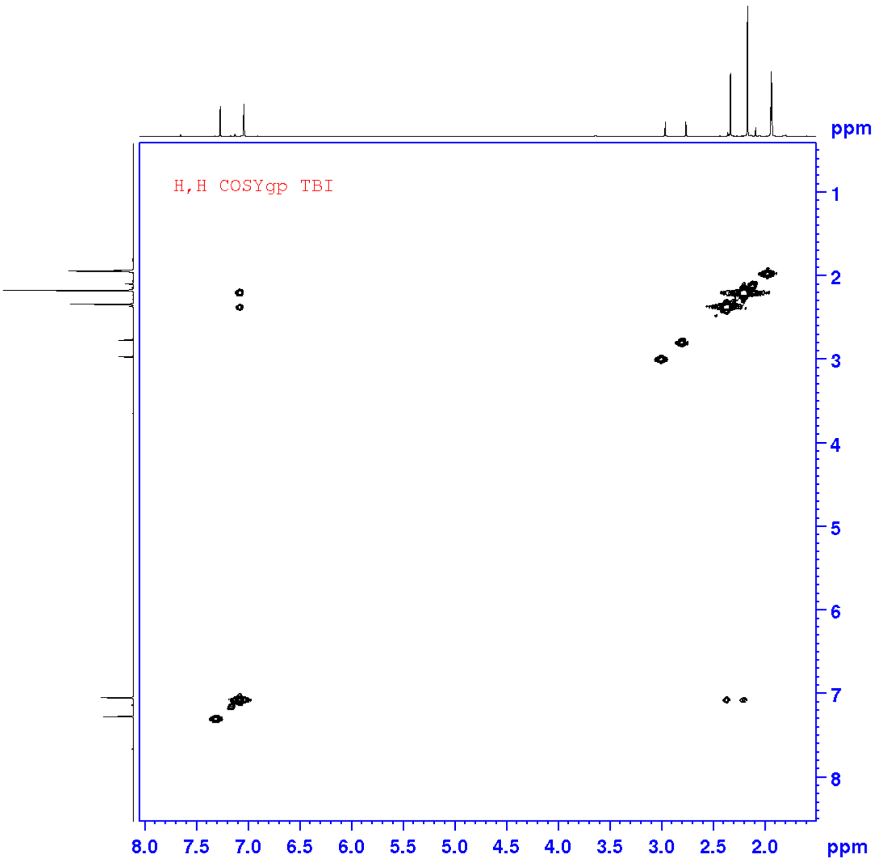

Figure S37. ^{1}H, ^{1}H(600MHz) COSY NMR spectrum of **3ae** ([D3]MeCN, 298 K).

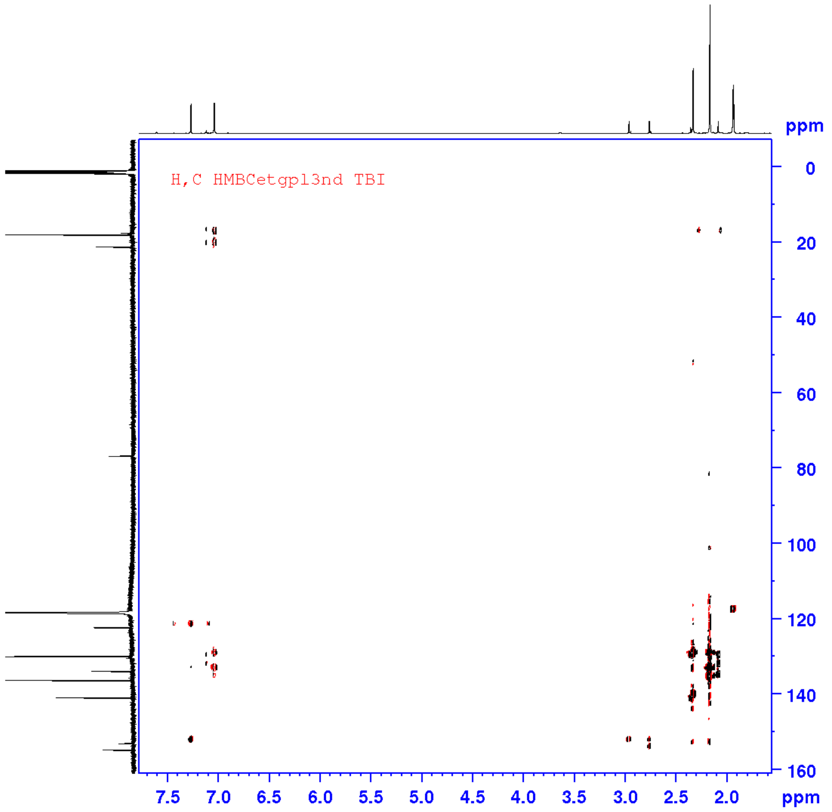

Figure S38. ^{1}H(600 MHz),^{13}C(150 MHz) HMBC NMR spectrum of **3ae** ([D3]MeCN, 298 K).

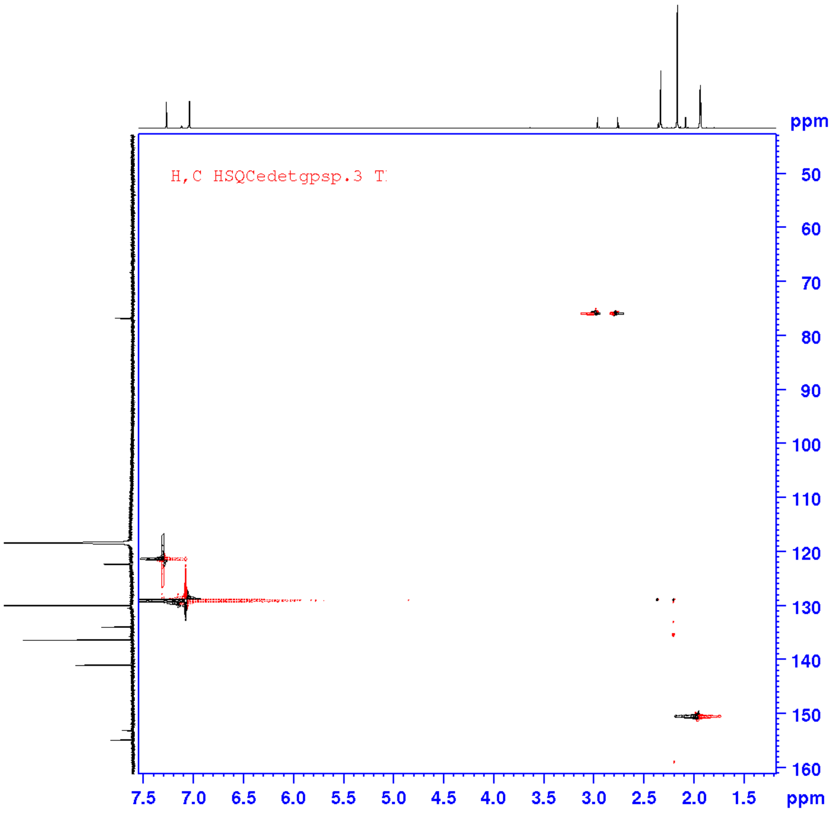

Figure S39. ^{1}H(600 MHz),^{13}C(150 MHz) HSQC NMR spectrum of **3ae** ([D3]MeCN, 298 K).

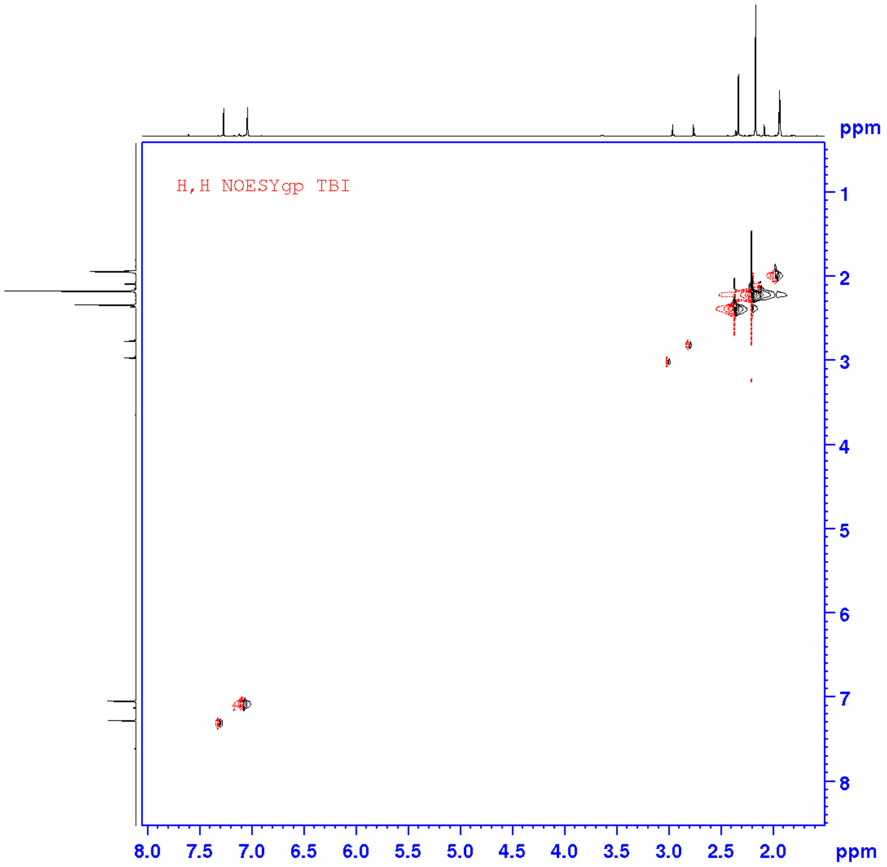

Figure S40. ^{1}H, ^{1}H(600MHz) NOESY NMR spectrum of **3ae** ([D3]MeCN, 298 K).

10.5 1D and 2D NMR spectra of 1-{1,3-bis[2,6-di(propan-2-yl)phenyl]-4,5-dihydro-1*H*-imidazol-3-ium-2-yl}-ethanone trifluoromethanesulfonate 1aa•OTf

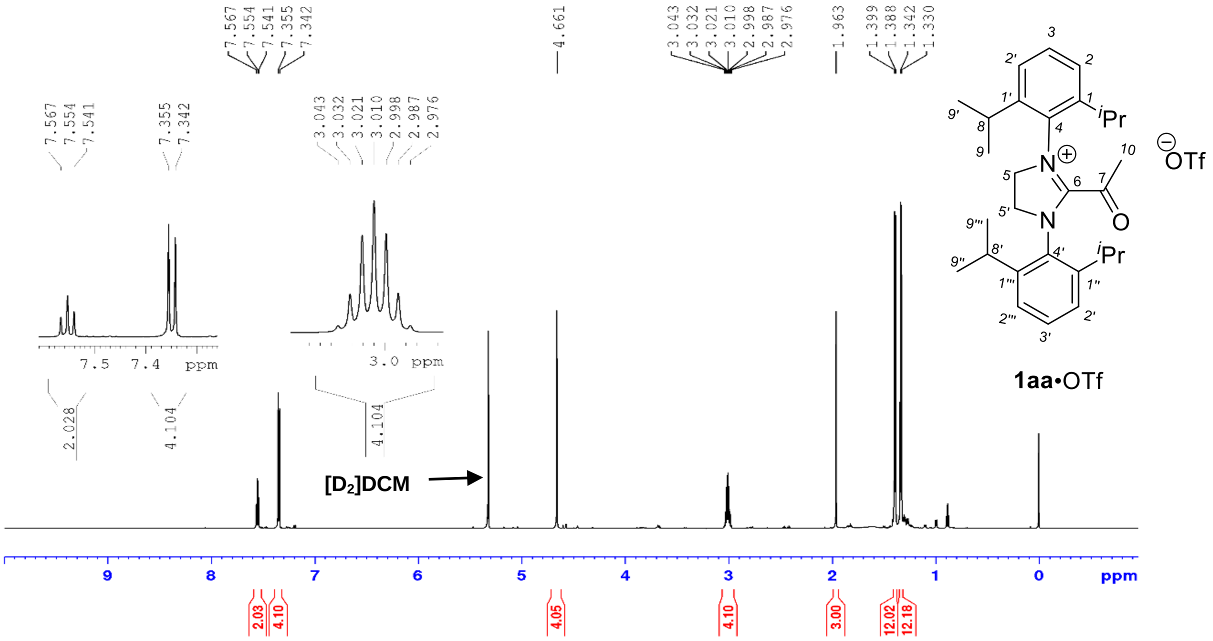

Figure S41. ^{1}H(600 MHz) NMR spectrum of **1aa**•OTf ([D2]DCM, 298 K).

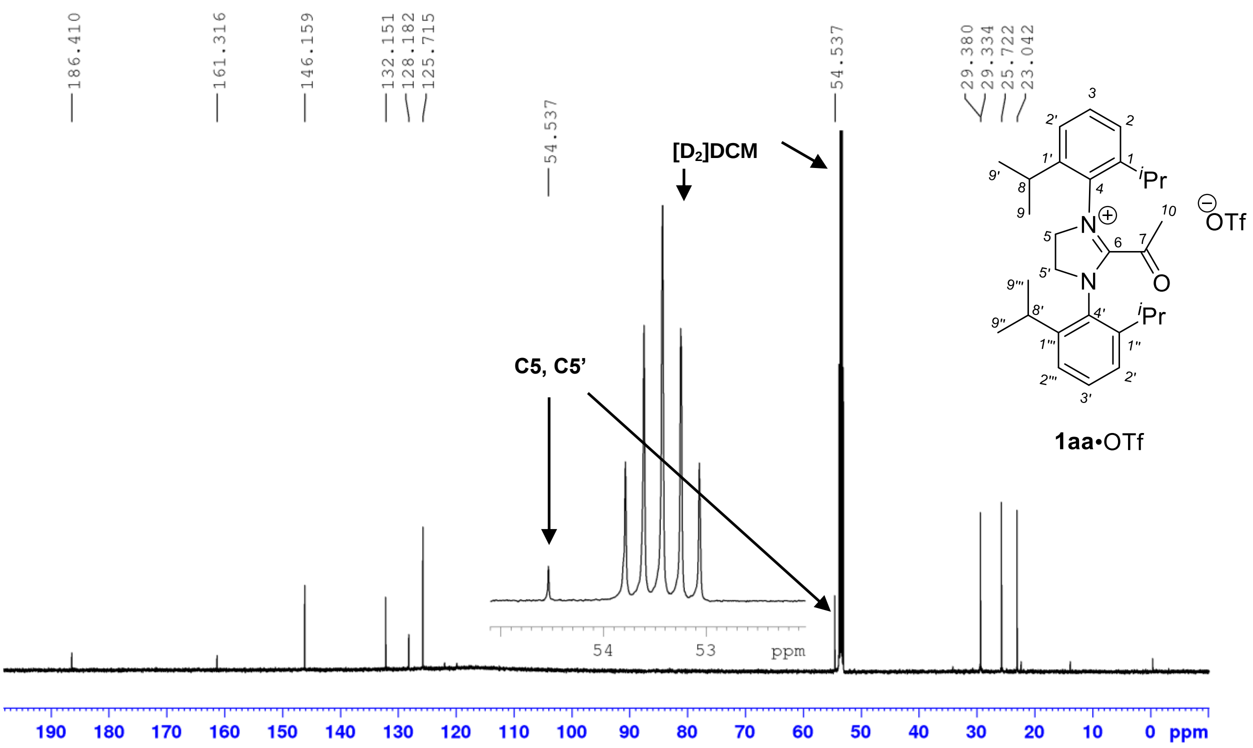

Figure S42. ^{13}C(150 MHz) NMR spectrum of **1aa•**OTf ([D$_2$]DCM, 298 K).

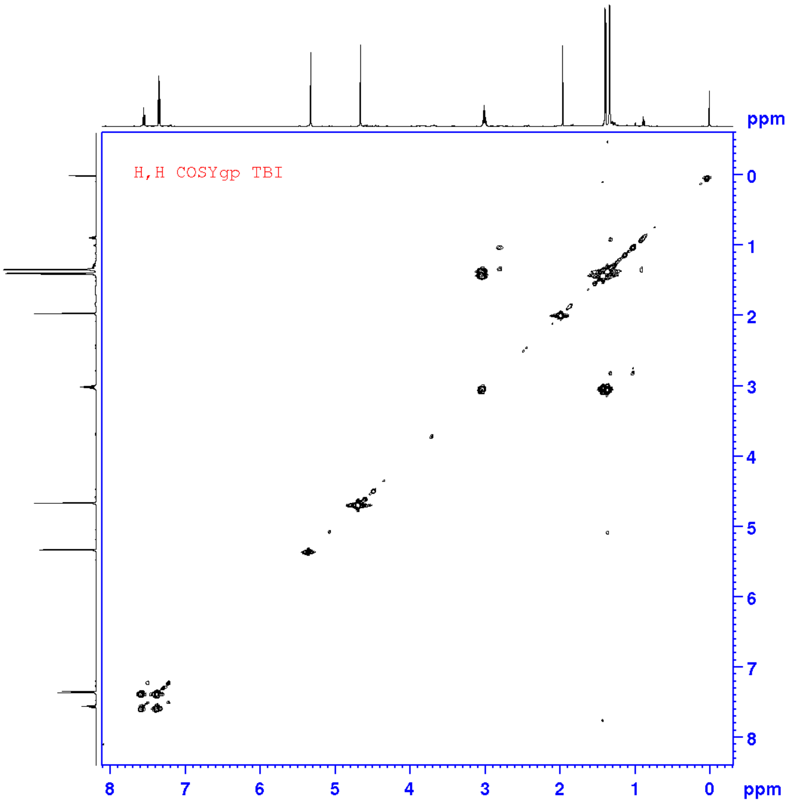

Figure S43. ^{1}H, ^{1}H(600MHz) COSY NMR spectrum of **1aa•**OTf ([D$_2$]DCM, 298 K).

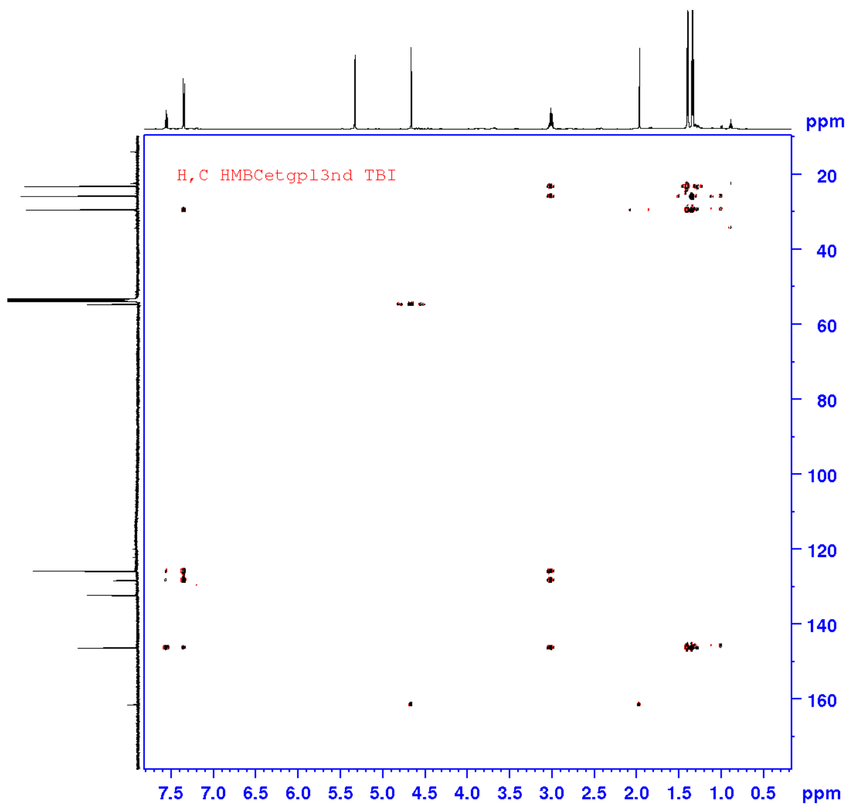

Figure S44. ^{1}H(600 MHz),^{13}C(150 MHz) HMBC NMR spectrum of **1aa**•OTf ([D2]DCM, 298 K).

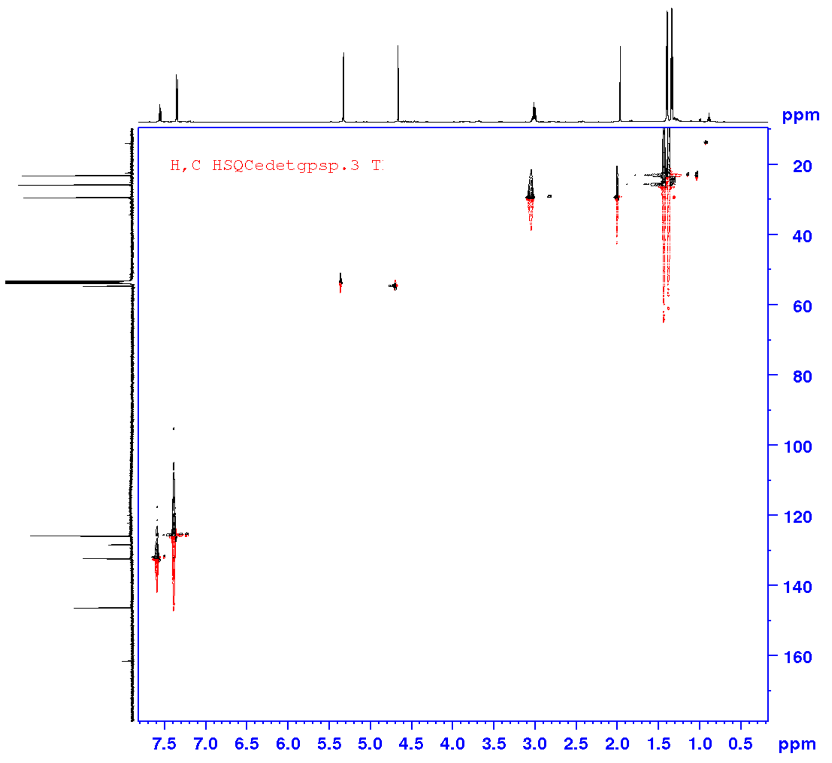

Figure S45. ^{1}H(600 MHz),^{13}C(150 MHz) HSQC NMR spectrum of **1aa**•OTf ([D2]DCM, 298 K).

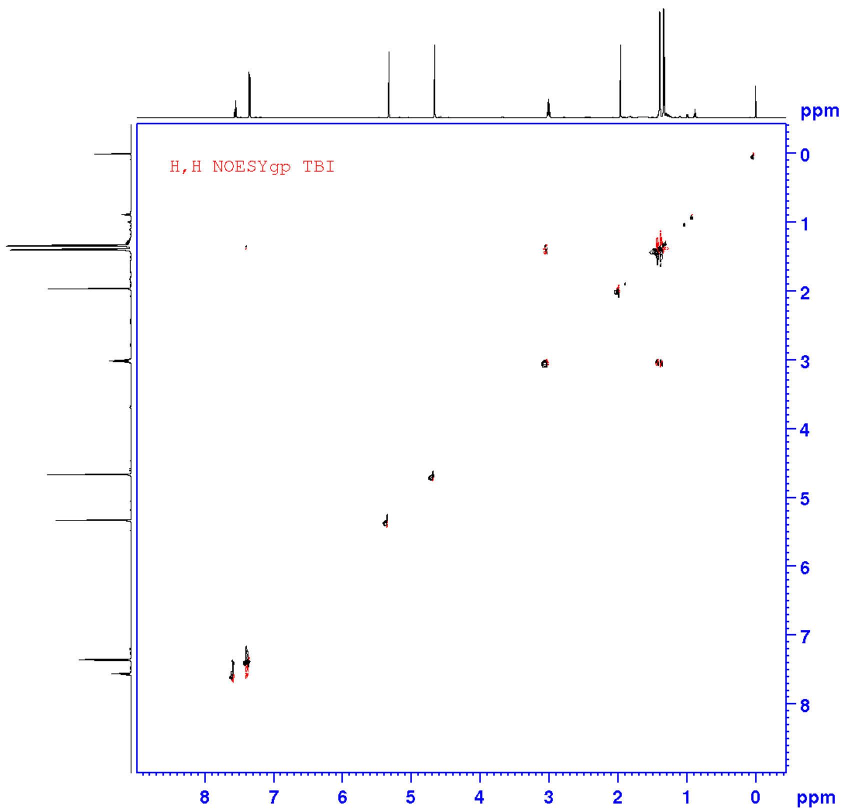

Figure S46. ^{1}H, ^{1}H(600MHz) NOESY NMR spectrum of **1aa•**OTf ([D2]DCM, 298 K).

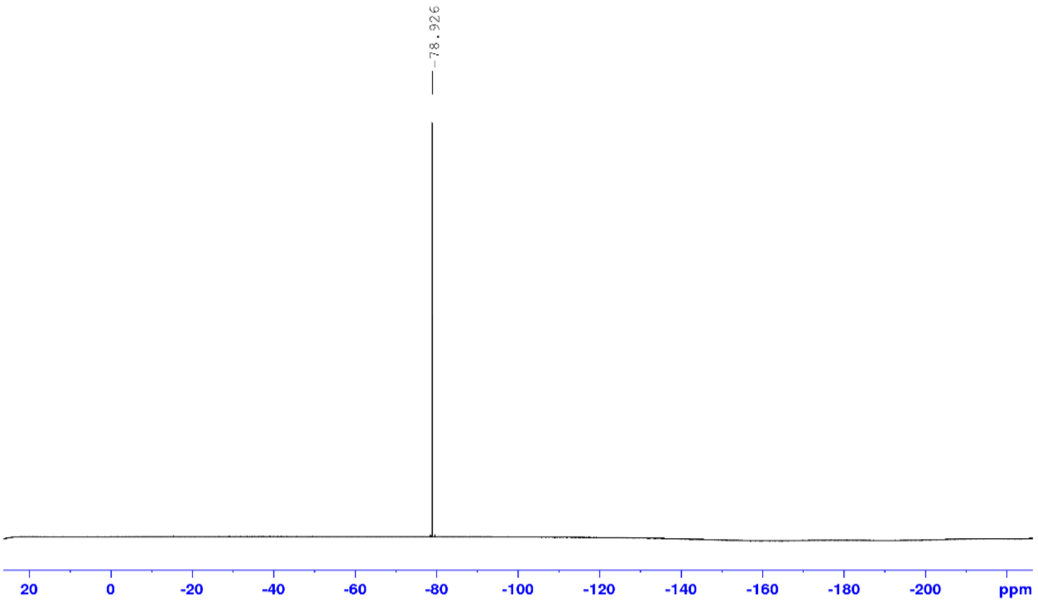

Figure S47. ^{19}F(282.231MHz) NMR spectrum of **1aa•**OTf ([D2]DCM, 298 K).

10.6 1D and 2D NMR spectra of 1-{1,3-bis[2,6-di(propan-2-yl)phenyl]-4,5-dihydro-1*H*-imidazol-3-ium-2-yl}-ethanone trifluoromethanesulfonate 2aa•OTf

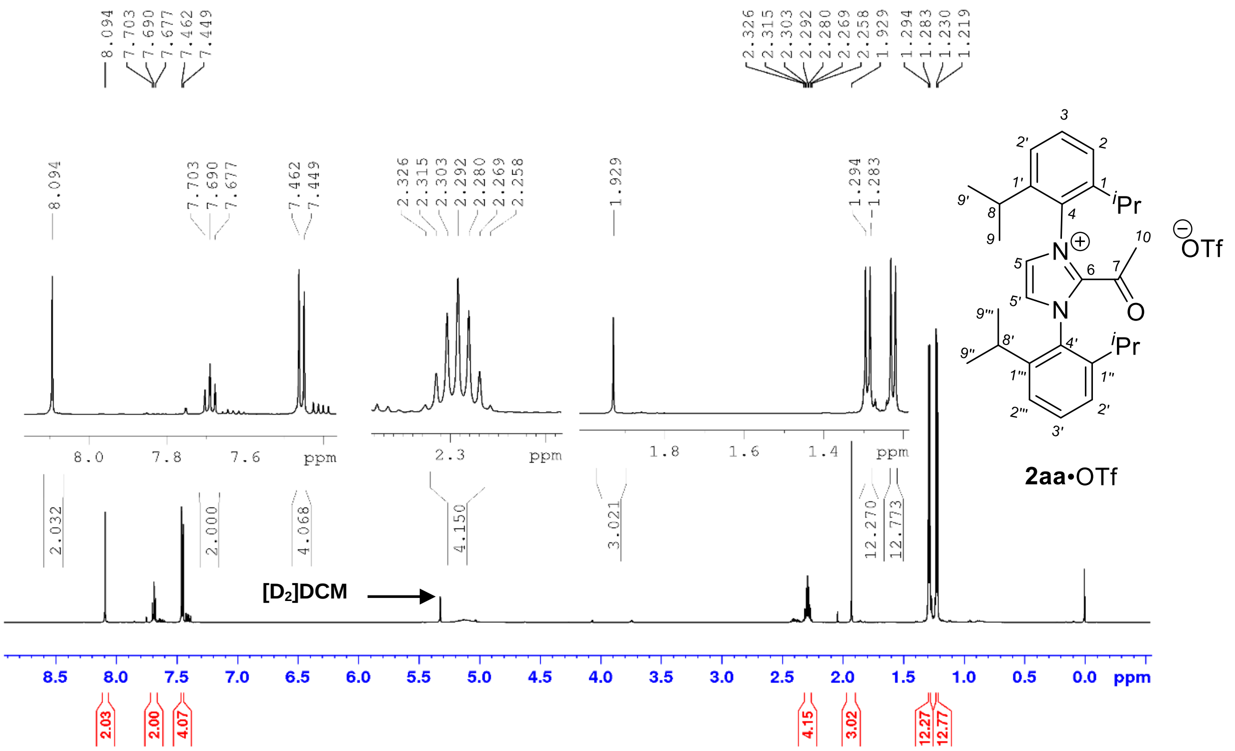

Figure S48. ^{1}H(600 MHz) NMR spectrum of **2aa**•OTf ([D$_2$]DCM, 298 K).

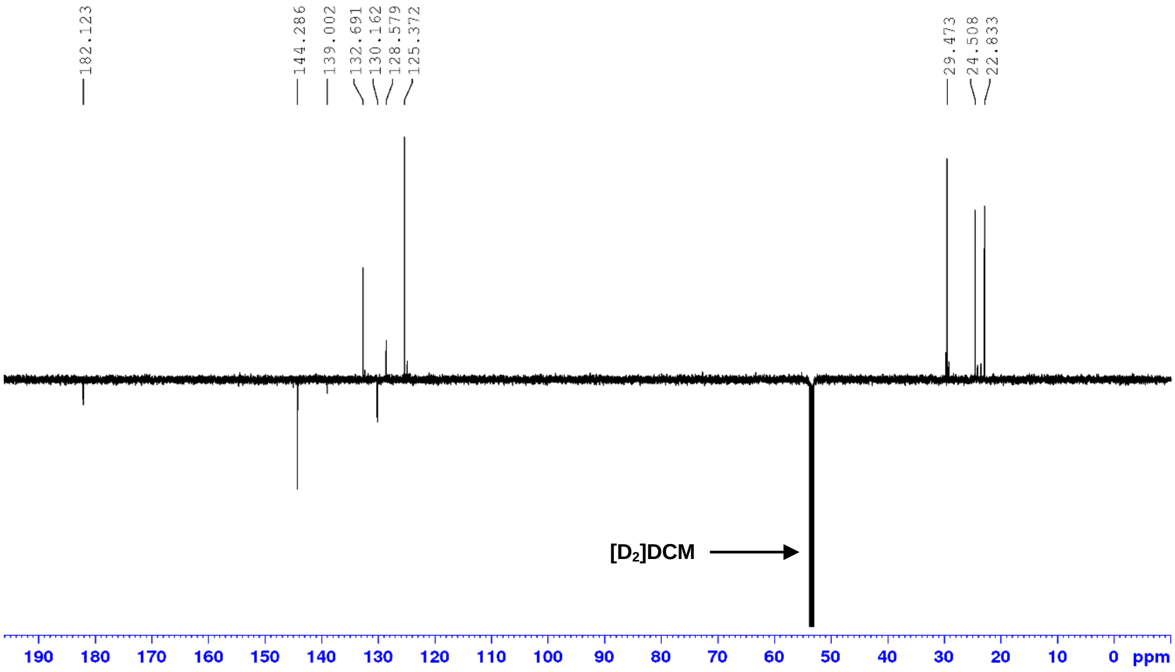

Figure S49. ^{13}C(150 MHz) DEPTQ NMR spectrum of **2aa**•OTf ([D$_2$]DCM, 298 K).

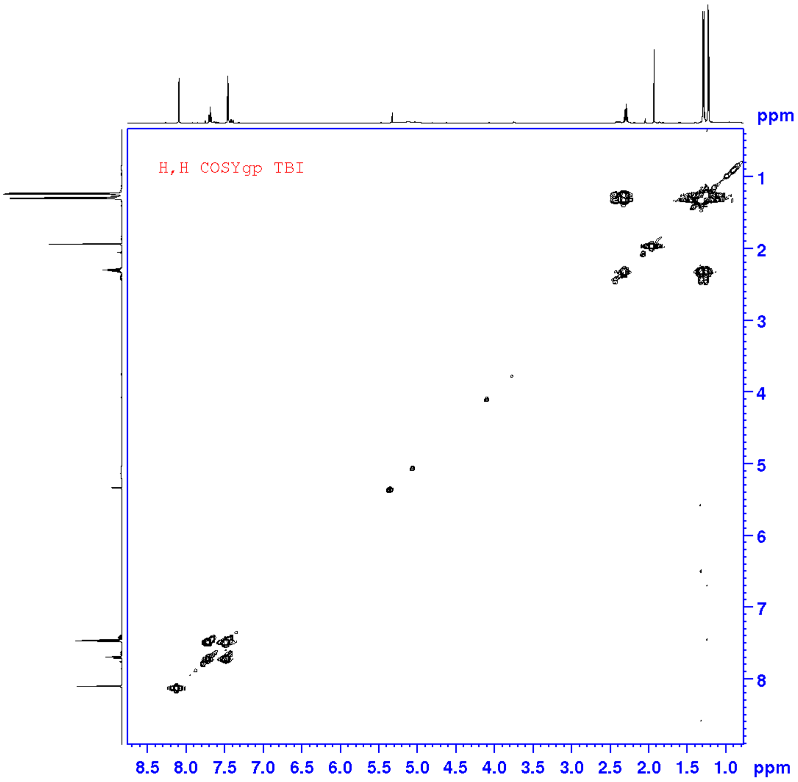

Figure S50. ^{1}H, ^{1}H(600MHz) COSY NMR spectrum of **2aa•**OTf ([D2]DCM, 298 K).

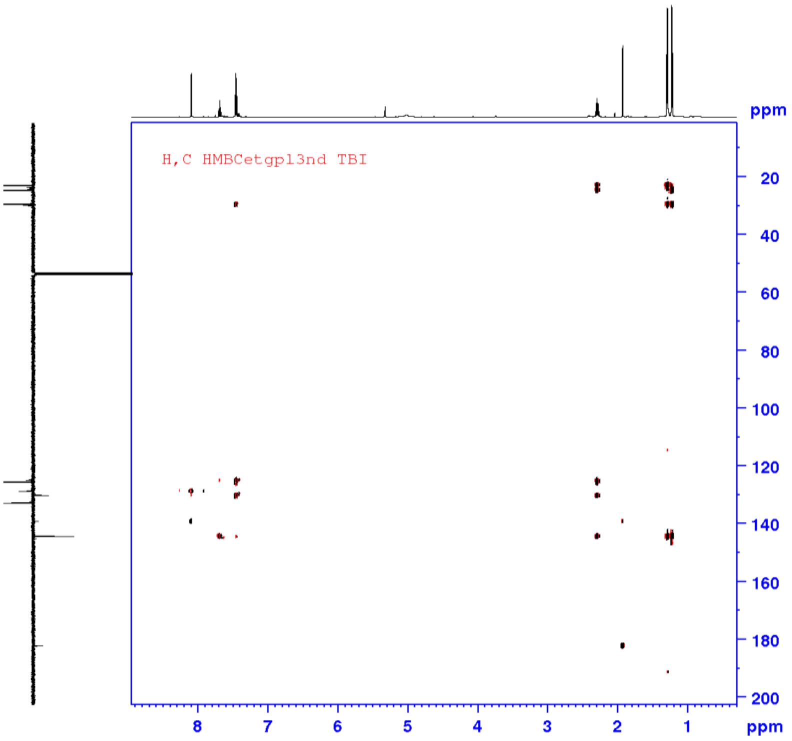

Figure S51. ^{1}H(600 MHz),^{13}C(150 MHz) HMBC NMR spectrum of **2aa•**OTf ([D2]DCM, 298 K).

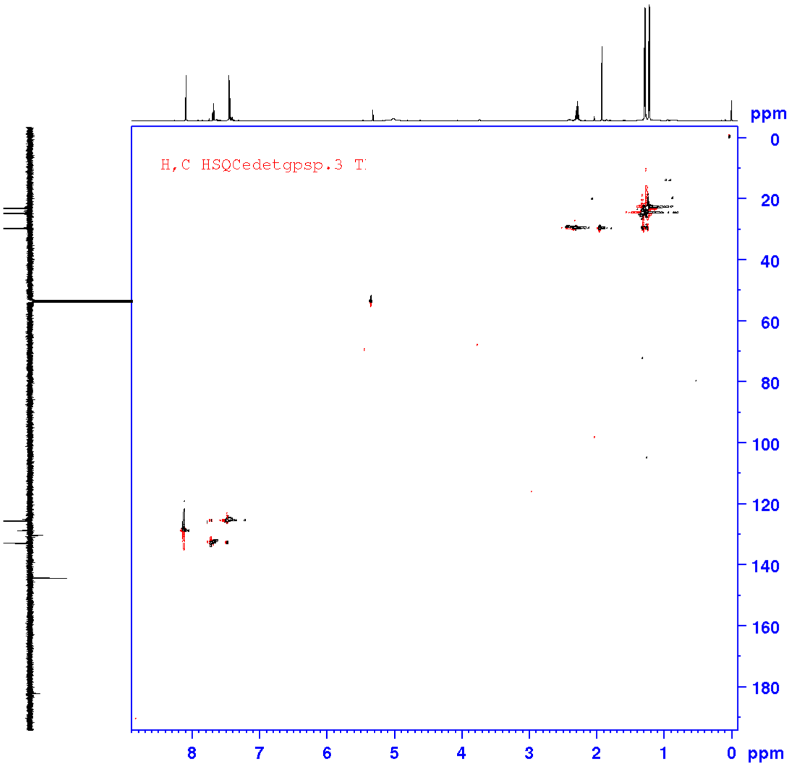

Figure S52. ^{1}H(600 MHz),^{13}C(150 MHz) HSQC NMR spectrum of **2aa•**OTf ([D2]DCM, 298 K).

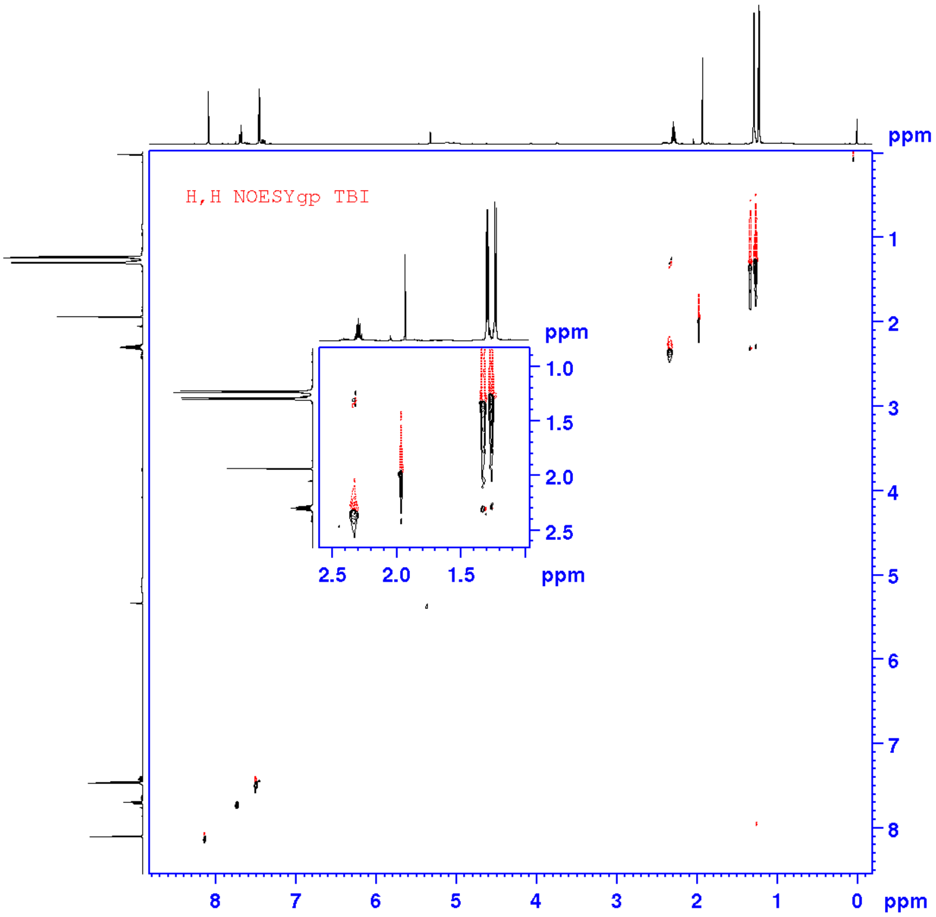

Figure S53. ^{1}H, ^{1}H(600MHz) NOESY NMR spectrum of **2aa•**OTf ([D2]DCM, 298 K).

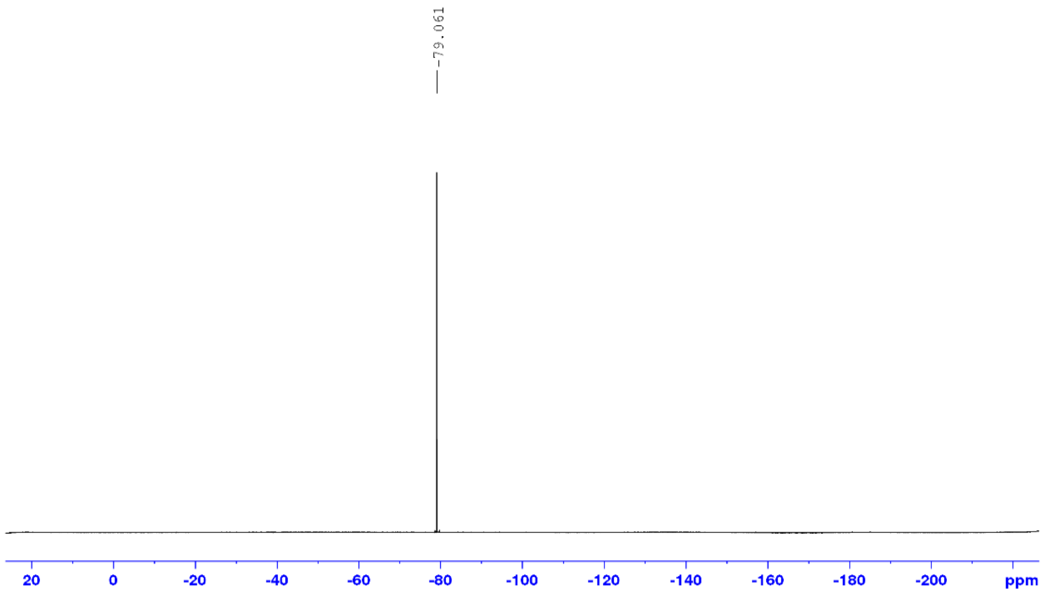

Figure S54. ^{19}F(282.231MHz) NMR spectrum of **2aa•**OTf ([D$_2$]DCM, 298 K).

10.7 ^{1}H NMR of deuterated benzyl alcohol

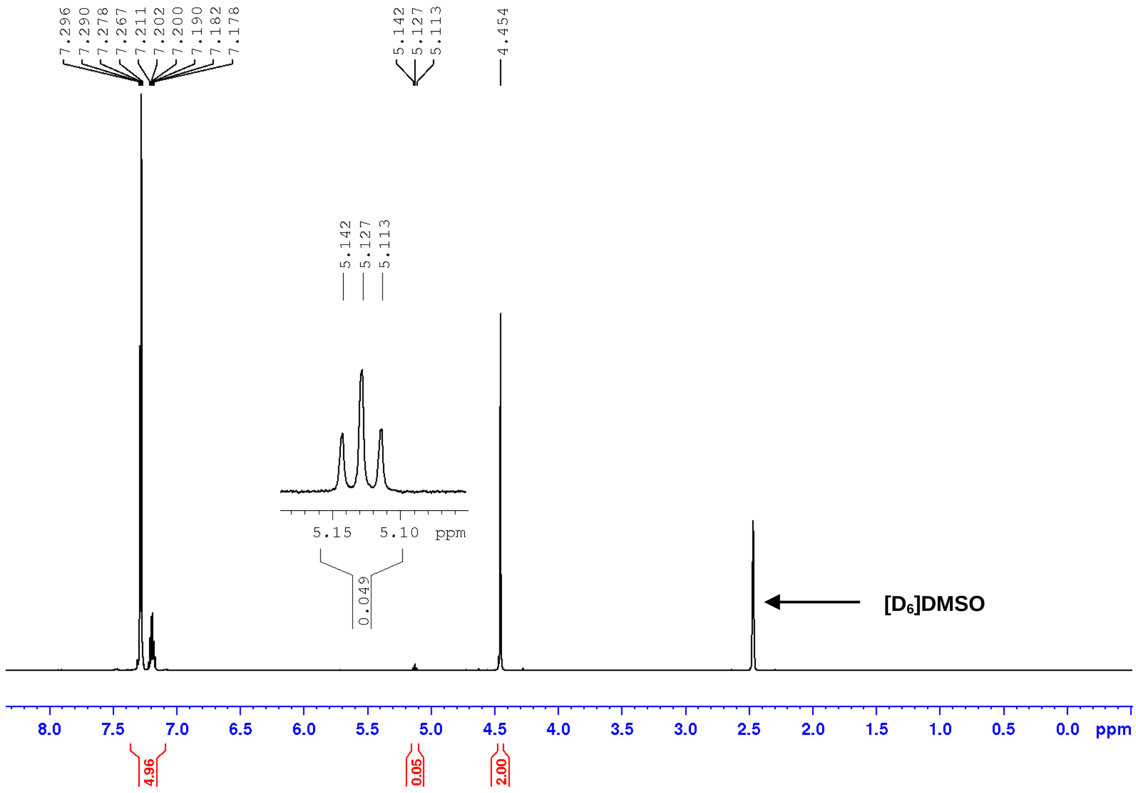

Figure S55. ^{1}H (600 MHz) NMR spectrum of **BnOD** (95% D) ([D$_6$]DMSO, 298 K).

11 X-ray data: Crystal data and structure refinement, ORTEPs.

11.1 X-ray data of {1,3-bis[2,6-di(propan-2-yl)phenyl]-4,5-dihydro-1*H*-imidazol-3-ium-2-yl}ethenolate 1ae

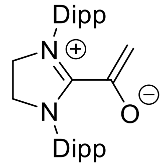

1ae

CCDC number	2008573	
Identification code	anb47_maerz2020a	
Empirical formula	$C_{29} H_{40} N_2 O$	
Moiety formula	$C_{29} H_{40} N_2 O$	
Formula weight	432.63	
Temperature	100(2) K	
Wavelength	1.54178 Å	
Crystal system	Monoclinic	
Space group	$P2_1/c$	
Unit cell dimensions	a = 21.0018(4) Å	α= 90°.
	b = 19.4069(4) Å	β= 109.2380(10)°.
	c = 27.4645(5) Å	γ = 90°.
Volume	10568.9(4) $Å^3$	
Z	16	
Density (calculated)	1.088 Mg/m^3	
Absorption coefficient	0.497 mm^{-1}	
F(000)	3776	
Crystal size	0.400 x 0.080 x 0.030 mm^3	
Theta range for data collection	2.228 to 72.283°.	
Index ranges	-25<=h<=25, -23<=k<=23, -33<=l<=30	
Reflections collected	563018	
Independent reflections	20810 [R(int) = 0.1316]	
Completeness to theta = 67.679°	100.0 %	
Absorption correction	Semi-empirical from equivalents	
Max. and min. transmission	0.7536 and 0.6822	
Refinement method	Full-matrix least-squares on F^2	
Data / restraints / parameters	20810 / 0 / 1223	
Goodness-of-fit on F^2	1.037	
Final R indices [I>2sigma(I)]	R1 = 0.0530, wR2 = 0.1346	
R indices (all data)	R1 = 0.0615, wR2 = 0.1379	
Extinction coefficient	n/a	
Largest diff. peak and hole	0.830 and -0.670 e.$Å^{-3}$	

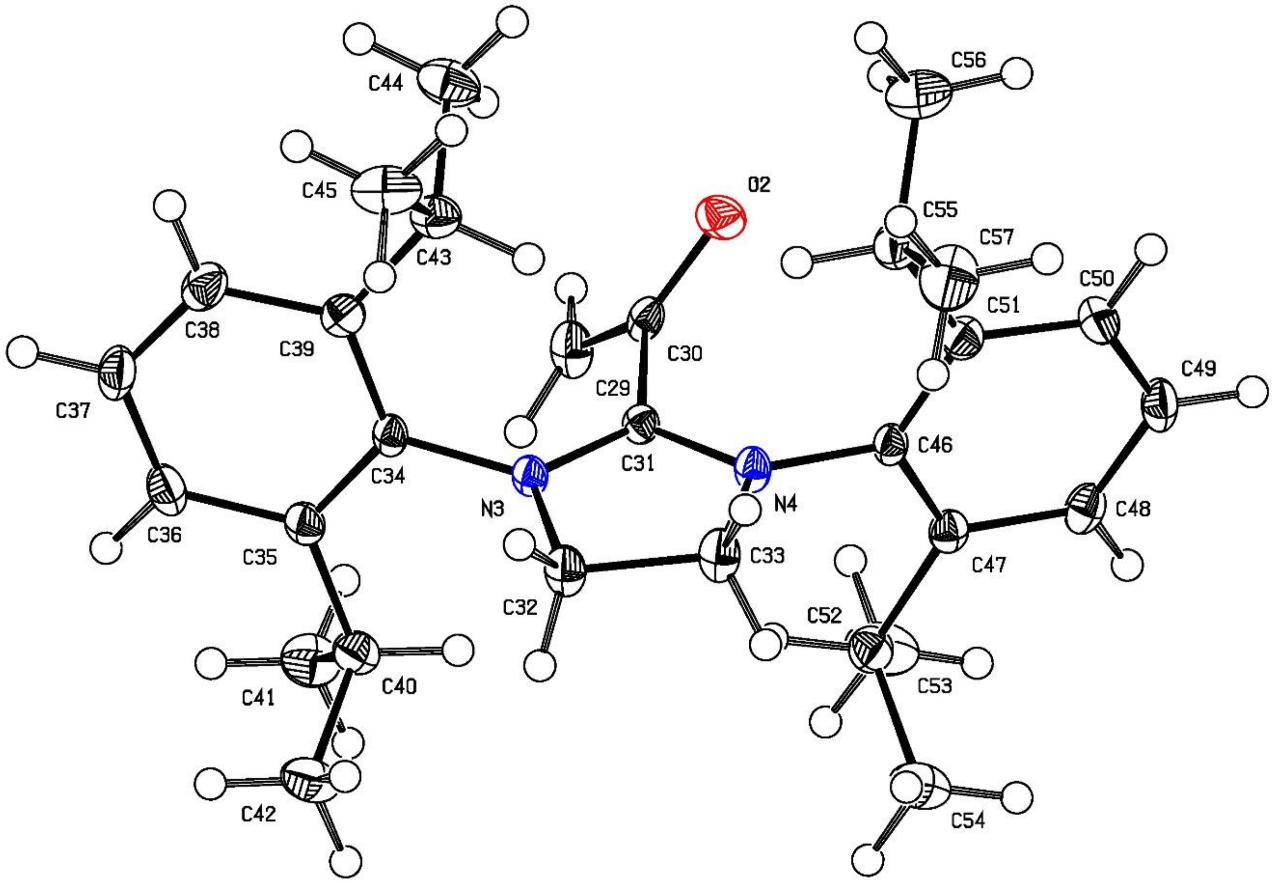

Figure S56. ORTEP of the X-ray crystal structure of **1ae**. Thermal ellipsoids are drawn at 50% probability level.

11.2 X-ray data of {1,3-bis[2,6-di(propan-2-yl)phenyl]-1*H*-imidazol-3-ium-2-yl}ethenolate 2ae

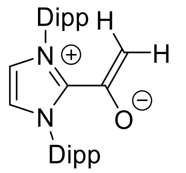

2ae

CCDC number	2008571	
Identification code	anb4069nn_tw	
Empirical formula	$C_{29} H_{38} N_2 O$	
Moiety formula	$C_{29} H_{38} N_2 O$	
Formula weight	430.61	
Temperature	100(2) K	
Wavelength	1.54178 Å	
Crystal system	Monoclinic	
Space group	$P2_1/c$	
Unit cell dimensions	a = 20.8859(7) Å	α = 90°.
	b = 19.2882(6) Å	β = 109.4330(10)°.
	c = 13.9080(4) Å	γ = 90°.
Volume	5283.7(3) Å^3	
Z	8	
Density (calculated)	1.083 Mg/m^3	
Absorption coefficient	0.497 mm^{-1}	
F(000)	1872	
Crystal size	0.250 x 0.100 x 0.060 mm^3	
Theta range for data collection	2.243 to 72.929°.	
Index ranges	-25<=h<=25, -23<=k<=23, -15<=l<=17	
Reflections collected	69185	
Independent reflections	69185 [R(int) = ?]	
Completeness to theta = 67.679°	100.0 %	
Absorption correction	Semi-empirical from equivalents	
Max. and min. transmission	0.7536 and 0.6674	
Refinement method	Full-matrix least-squares on F^2	
Data / restraints / parameters	69185 / 0 / 594	
Goodness-of-fit on F^2	1.028	
Final R indices [I>2sigma(I)]	R1 = 0.0548, wR2 = 0.1227	
R indices (all data)	R1 = 0.0821, wR2 = 0.1340	
Extinction coefficient	n/a	
Largest diff. peak and hole	0.523 and -0.353 e.Å^{-3}	

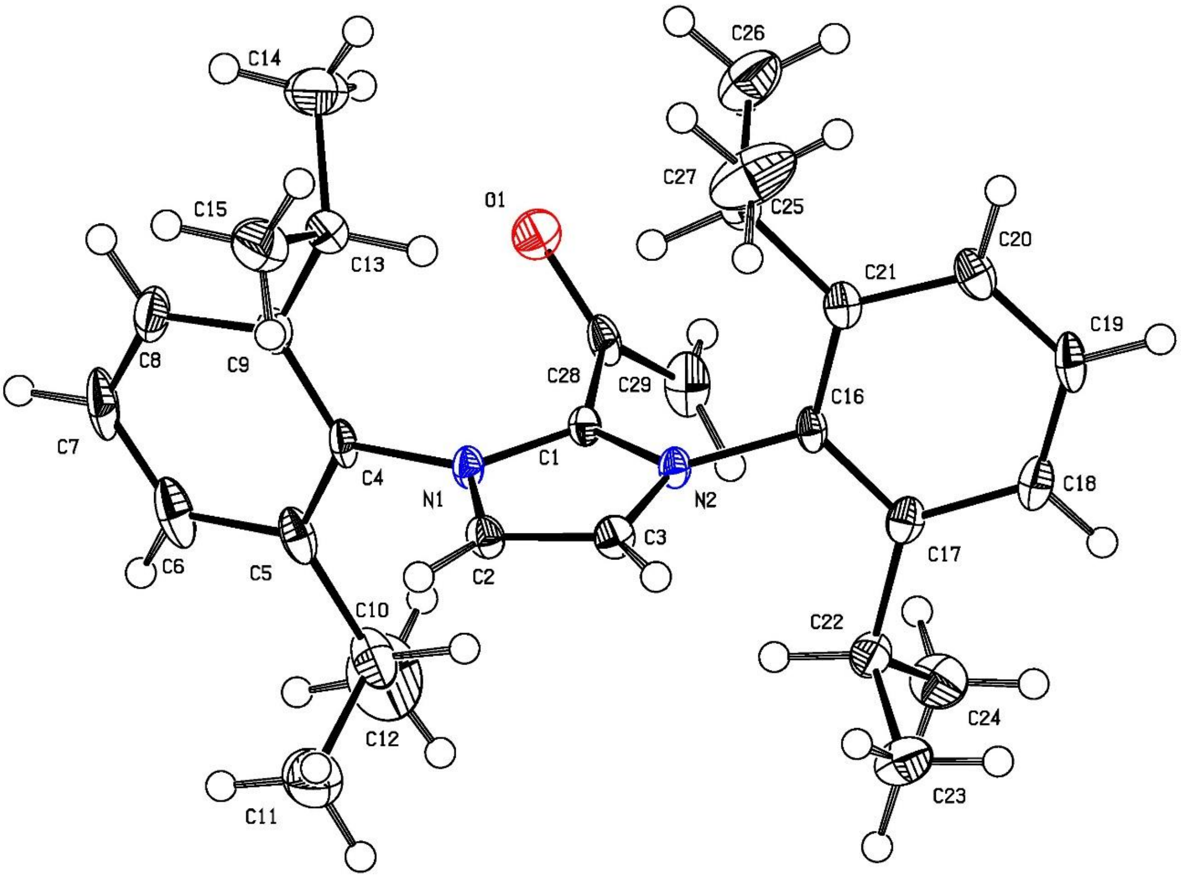

Figure S57. ORTEP of the X-ray crystal structure of **2ae**. Thermal ellipsoids are drawn at 50% probability level.

11.3 X-ray data of 1-{1,3-bis[2,6-di(propan-2-yl)phenyl]-4,5-dihydro-1*H*-imidazol-3-ium-2-yl}-ethanone trifluoromethanesulfonate 1aa•OTf

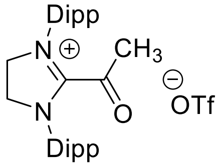

1aa•OTf

CCDC number	2008572	
Identification code	anb59	
Empirical formula	$C_{30} H_{41} F_3 N_2 O_4 S$	
Moiety formula	$C_{29} H_{41} N_2$; $S C F_3 O_4$	
Formula weight	582.71	
Temperature	100(2) K	
Wavelength	1.54178 Å	
Crystal system	Orthorhombic	
Space group	Pbca	
Unit cell dimensions	a = 15.9529(14) Å	α = 90°.
	b = 16.9145(13) Å	β = 90°.
	c = 22.4530(18) Å	γ = 90°.
Volume	6058.6(9) $Å^3$	
Z	8	
Density (calculated)	1.278 Mg/m^3	
Absorption coefficient	1.419 mm^{-1}	
F(000)	2480	
Crystal size	0.200 x 0.200 x 0.100 mm^3	
Theta range for data collection	3.937 to 72.380°.	
Index ranges	-19<=h<=19, -20<=k<=20, -26<=l<=27	
Reflections collected	53265	
Independent reflections	5824 [R(int) = 0.0567]	
Completeness to theta = 67.679°	97.3 %	
Absorption correction	Semi-empirical from equivalents	
Max. and min. transmission	0.7536 and 0.5570	
Refinement method	Full-matrix least-squares on F^2	
Data / restraints / parameters	5824 / 0 / 370	
Goodness-of-fit on F^2	1.026	
Final R indices [I>2sigma(I)]	R1 = 0.0637, wR2 = 0.1603	
R indices (all data)	R1 = 0.0732, wR2 = 0.1674	
Extinction coefficient	n/a	
Largest diff. peak and hole	2.215 and -0.890 e.$Å^{-3}$	

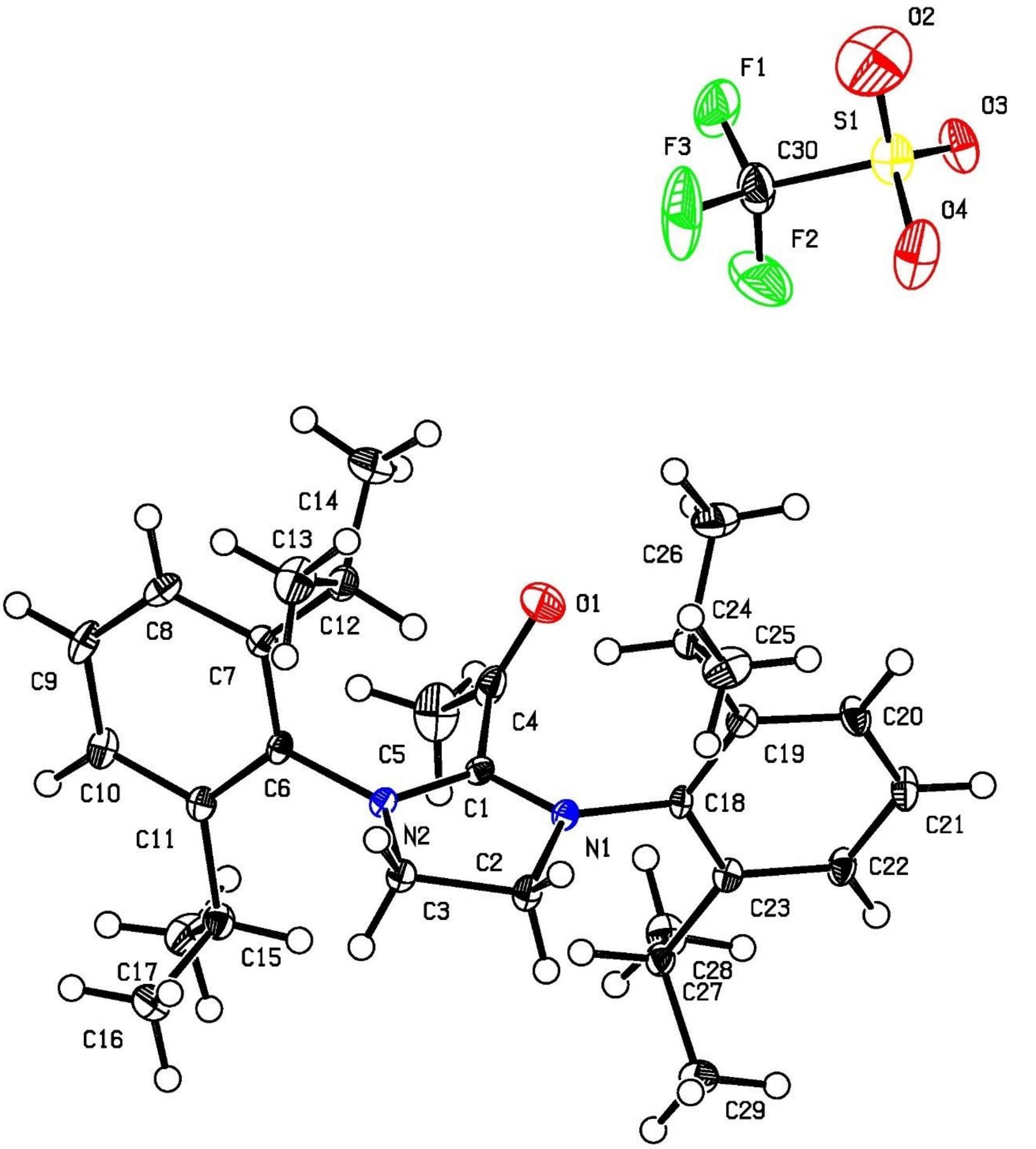

Figure S58. ORTEP of the X-ray crystal structure of **1aa•**OTf. Thermal ellipsoids are drawn at 50% probability level.

11.4 X-ray data of 1-{1,3-bis[2,6-di(propan-2-yl)phenyl]-1*H*-imidazol-3-ium-2-yl}-ethanone trifluoromethanesulfonate 2aa•OTf

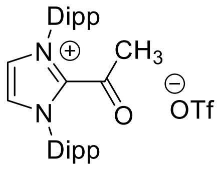

2aa•OTf

CCDC number	2008570	
Identification code	anb4072	
Empirical formula	$C_{30} H_{39} F_3 N_2 O_4 S$	
Moiety formula	$C_{29} H_{39} N_2 O; C F_3 O_3 S$	
Formula weight	580.69	
Temperature	100(2) K	
Wavelength	1.54178 Å	
Crystal system	Triclinic	
Space group	P-1	
Unit cell dimensions	a = 10.3286(3) Å	α = 73.8850(10)°.
	b = 12.4939(4) Å	β = 84.2860(10)°.
	c = 12.7263(4) Å	γ = 71.8740(10)°.
Volume	1499.28(8) $Å^3$	
Z	2	
Density (calculated)	1.286 Mg/m^3	
Absorption coefficient	1.434 mm^{-1}	
F(000)	616	
Crystal size	0.300 x 0.200 x 0.200 mm^3	
Theta range for data collection	3.615 to 72.201°.	
Index ranges	-12<=h<=12, -15<=k<=15, -15<=l<=15	
Reflections collected	34079	
Independent reflections	5879 [R(int) = 0.0354]	
Completeness to theta = 67.679°	99.8 %	
Absorption correction	Semi-empirical from equivalents	
Max. and min. transmission	0.7536 and 0.6244	
Refinement method	Full-matrix least-squares on F^2	
Data / restraints / parameters	5879 / 0 / 371	
Goodness-of-fit on F^2	1.039	
Final R indices [I>2sigma(I)]	R1 = 0.0331, wR2 = 0.0842	
R indices (all data)	R1 = 0.0346, wR2 = 0.0856	
Extinction coefficient	0.0065(4)	
Largest diff. peak and hole	0.419 and -0.386 e.$Å^{-3}$	

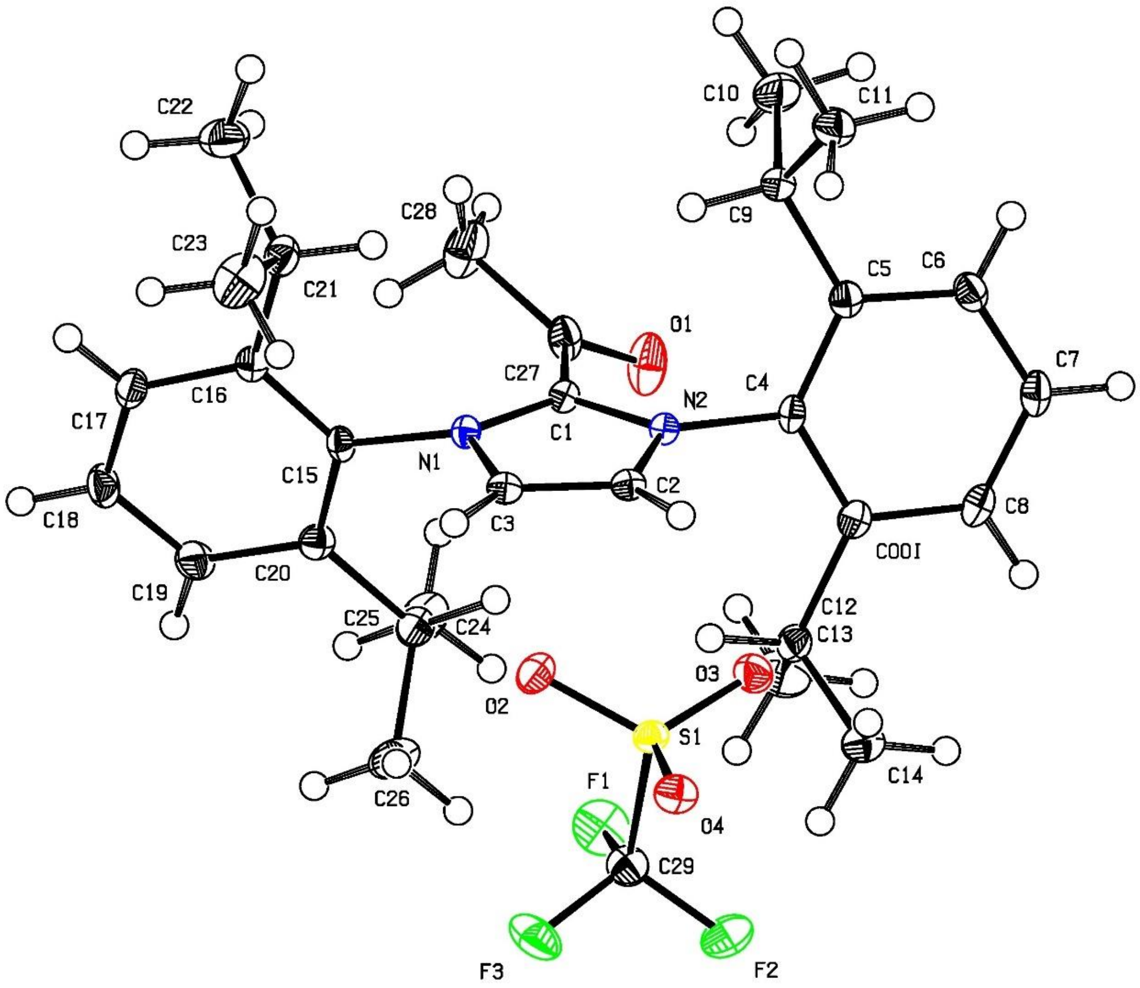

Figure S59. ORTEP of the X-ray crystal structure of **2aa**•OTf. Thermal ellipsoids are drawn at 50% probability level.

12 References

[1] H. Lundberg, F. Tinnis, J. Zhang, A. G. Algarra, F. Himo, H. Adolfsson, *J. Am. Chem. Soc.* **2017**, *139*, 2286-2295.

[2] F. Tamaddon, M. A. Amrollahi, L. Sharafat, *Tetrahedron Lett.* **2005**, *46*, 7841-7844.

[3] Q. Liu, Y. Zhao, B. Hammann, J. Eilers, Y. Lu, A. Kohen, *J. Org. Chem.* **2012**, *77*, 6825-6833.
